# Aglycosylated extracellular loop of inwardly rectifying potassium channel 4.1 (KCNJ10) provides a target for autoimmune neuroinflammation

**DOI:** 10.1093/braincomms/fcad044

**Published:** 2023-02-22

**Authors:** Arnaud B Nicot, Jean Harb, Alexandra Garcia, Flora Guillot, Hoa-Le Mai, Camille V Mathé, Jérémy Morille, Amélie Vallino, Emilie Dugast, Sita P Shah, Fabienne Lefrère, Mélinda Moyon, Sandrine Wiertlewski, Ludmilla Le Berre, Karine Renaudin, Jean-Paul Soulillou, Vincent van Pesch, Sophie Brouard, Laureline Berthelot, David-Axel Laplaud

**Affiliations:** INSERM, Nantes Université, CHU Nantes, Center for Research in Transplantation and Translational Immunology (CR2TI), UMR 1064, Nantes 44000, France; INSERM, Nantes Université, CHU Nantes, Center for Research in Transplantation and Translational Immunology (CR2TI), UMR 1064, Nantes 44000, France; INSERM, Nantes Université, CHU Nantes, Center for Research in Transplantation and Translational Immunology (CR2TI), UMR 1064, Nantes 44000, France; INSERM, Nantes Université, CHU Nantes, Center for Research in Transplantation and Translational Immunology (CR2TI), UMR 1064, Nantes 44000, France; INSERM, Nantes Université, CHU Nantes, Center for Research in Transplantation and Translational Immunology (CR2TI), UMR 1064, Nantes 44000, France; INSERM, Nantes Université, CHU Nantes, Center for Research in Transplantation and Translational Immunology (CR2TI), UMR 1064, Nantes 44000, France; INSERM, Nantes Université, CHU Nantes, Center for Research in Transplantation and Translational Immunology (CR2TI), UMR 1064, Nantes 44000, France; INSERM, Nantes Université, CHU Nantes, Center for Research in Transplantation and Translational Immunology (CR2TI), UMR 1064, Nantes 44000, France; INSERM, Nantes Université, CHU Nantes, Center for Research in Transplantation and Translational Immunology (CR2TI), UMR 1064, Nantes 44000, France; INSERM, Nantes Université, CHU Nantes, Center for Research in Transplantation and Translational Immunology (CR2TI), UMR 1064, Nantes 44000, France; Service de Neurologie, CHU Nantes, Nantes 44000, France; CIC Inserm 1413, CHU Nantes, Nantes 44000, France; Service de Neurologie, CHU Nantes, Nantes 44000, France; CIC Inserm 1413, CHU Nantes, Nantes 44000, France; INSERM, Nantes Université, CHU Nantes, Center for Research in Transplantation and Translational Immunology (CR2TI), UMR 1064, Nantes 44000, France; Service de Neurologie, CHU Nantes, Nantes 44000, France; CIC Inserm 1413, CHU Nantes, Nantes 44000, France; INSERM, Nantes Université, CHU Nantes, Center for Research in Transplantation and Translational Immunology (CR2TI), UMR 1064, Nantes 44000, France; INSERM, Nantes Université, CHU Nantes, Center for Research in Transplantation and Translational Immunology (CR2TI), UMR 1064, Nantes 44000, France; INSERM, Nantes Université, CHU Nantes, Center for Research in Transplantation and Translational Immunology (CR2TI), UMR 1064, Nantes 44000, France; Neurologie, Institute of Neuroscience, Université Catholique de Louvain, Bruxelles 1200, Belgium; INSERM, Nantes Université, CHU Nantes, Center for Research in Transplantation and Translational Immunology (CR2TI), UMR 1064, Nantes 44000, France; INSERM, Nantes Université, CHU Nantes, Center for Research in Transplantation and Translational Immunology (CR2TI), UMR 1064, Nantes 44000, France; INSERM, Nantes Université, CHU Nantes, Center for Research in Transplantation and Translational Immunology (CR2TI), UMR 1064, Nantes 44000, France; Service de Neurologie, CHU Nantes, Nantes 44000, France; CIC Inserm 1413, CHU Nantes, Nantes 44000, France

**Keywords:** autoimmunity, glia, glycosylation, antibody, Kir4.1, EAE

## Abstract

Multiple sclerosis is an autoimmune disease of the central nervous system. Yet, the autoimmune targets are still undefined. The extracellular e1 sequence of KCNJ10, the inwardly rectifying potassium channel 4.1, has been subject to fierce debate for its role as a candidate autoantigen in multiple sclerosis. Inwardly rectifying potassium channel 4.1 is expressed in the central nervous system but also in peripheral tissues, raising concerns about the central nervous system-specificity of such autoreactivity. Immunization of C57Bl6/J female mice with the e1 peptide (amino acids 83–120 of Kir4.1) induced anti-e1 immunoglobulin G- and T-cell responses and promoted demyelinating encephalomyelitis with B cell central nervous system enrichment in leptomeninges and T cells/macrophages in central nervous system parenchyma from forebrain to spinal cord, mostly in the white matter. Within our cohort of multiple sclerosis patients (*n* = 252), 6% exhibited high anti-e1 immunoglobulin G levels in serum as compared to 0.7% in the control cohort (*n* = 127; *P* = 0.015). Immunolabelling of inwardly rectifying potassium channel 4.1-expressing white matter glia with the anti-e1 serum from immunized mice increased during murine autoimmune neuroinflammation and in multiple sclerosis white matter as compared with controls. Strikingly, the mouse and human anti-e1 sera labelled astrocytoma cells when *N*-glycosylation was blocked with tunicamycin. Western blot confirmed that neuroinflammation induces Kir4.1 expression, including its shorter aglycosylated form in murine experimental autoencephalomyelitis and multiple sclerosis. In addition, recognition of inwardly rectifying potassium channel 4.1 using mouse anti-e1 serum in Western blot experiments under unreduced conditions or in cells transfected with the *N*-glycosylation defective N104Q mutant as compared to the wild type further suggests that autoantibodies target an e1 conformational epitope in its aglycosylated form. These data highlight the e1 sequence of inwardly rectifying potassium channel 4.1 as a valid central nervous system autoantigen with a disease/tissue-specific post-translational antigen modification as potential contributor to autoimmunity in some multiple sclerosis patients.

## Teaser

Multiple sclerosis likely implies different CNS autoimmune targets. Nicot *et al.* show that immunizing mice with the aglycosylated e1 loop of the glial Kir4.1 can foster encephalomyelitis development and that anti-e1 reactivity may be specifically involved in CNS autoimmunity for a subset of multiple sclerosis patients.

## Graphical Abstract

**Graphical abstract:**
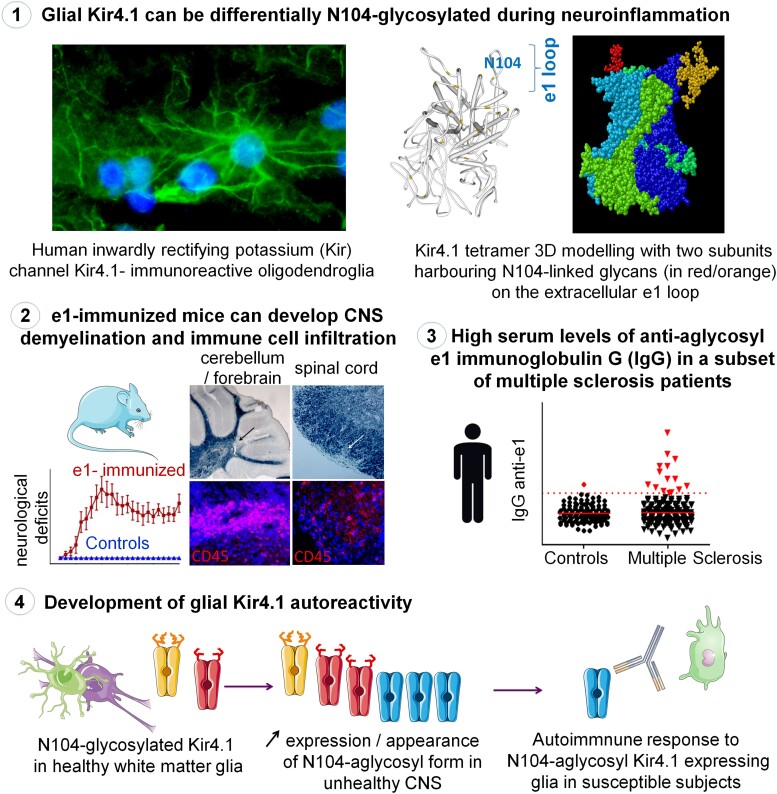

## Introduction

Multiple sclerosis is an inflammatory disease of the CNS where T and B cells directed against myelin are thought to play a deleterious role.^1,2^ Autoantibodies targeting the first extracellular domain (e1) of inwardly rectifying potassium (Kir) channel 4.1 (Kir4.1) had been initially reported in half of multiple sclerosis patients, suggesting Kir4.1 as a major candidate target for CNS autoimmunity.^3^ Variable lower frequencies have since been reported, raising concerns about Kir4.1 as a common autoantigen candidate for multiple sclerosis.^4,5^ While identification of few autoreactivity markers in multiple sclerosis would be a breakthrough, as in the case of *myasthenia gravis*,^6^ multiple sclerosis is increasingly viewed as a syndrome with various patterns of inflammation and demyelination that could involve different autoimmune CNS targets from neurons or glia.^7–12^ Initially, a small subset of patients^13,14^ were described as having high levels of autoantibodies against a conformational epitope of myelin oligodendrocyte glycoprotein (MOG), the most studied antigen candidate. Now, MOG–IgG-associated encephalomyelitis is considered a disease entity on its own.^15^ Thus, high Kir4.1 IgG-positive patients could also belong to a specific nosological entity within a larger multiple sclerosis spectrum disorder. However, the absence of an *in vivo* model showing deleterious effects of e1 autoreactivity in the CNS has impeded the consideration of Kir4.1 as a valid autoantigen candidate.

Inwardly rectifying potassium channel 4.1 (encoded from *KCNJ10 gene*) is expressed by astrocytes and oligodendroglia^16–20^ and plays a role in K + spatial buffering and myelination.^21–23^ The loss-of-function mutations in human KCNJ10 lead to SeSAME (seizures, sensorineural deafness, ataxia, mental impairment and renal electrolyte imbalance) syndrome.^24,25^ Glial Kir4.1 deficiency in mice results in ataxia, seizures, hindlimb paralysis, white matter (WM) vacuolization, growth retardation and premature death^26^ while Kir4.1 loss in kidney tubules accounts for the salt retention defect.^27^ In the context of multiple sclerosis, it is likely that e1 autoreactivity, if present, does not affect peripheral cells such as renal tubular cells. Increasing molecular insights into tissue-specificity and epitope-specific targeting is a key issue for understanding autoimmune diseases. Interestingly, the e1 sequence contains an asparagine at position 104, offering a potential *N*-glycosylation site,^28^ and molecular weight (MW) of Kir4.1 monomer in the brain has been reported lower than in the kidney.^29^ Post-translational modifications are increasingly recognized as determinant for antibody–antigen interactions by favouring either antigen recognition^11^ or immune camouflage via *N*-glycosylation.^30^ The use of recombinant Kir4.1 protein with high levels of *N*-glycosylation may partly explain the difficulty in determining Kir4.1 IgG ‘high responders’ in some of the previous studies.^31^ How anti-e1 autoreactivity could target CNS cells while sparing other cells that express high levels of Kir4.1 remains undefined. Here, we present *in vivo* and *in vitro* data that support the aglycosylated e1 loop of Kir4.1 as a valid CNS WM-specific antigen candidate, notably for a subset of multiple sclerosis patients that exhibit high anti-e1 IgG autoreactivity.

## Materials and methods

### Murine model of inwardly rectifying potassium channel 4.1 (e1)-induced autoimmune encephalomyelitis

C57BL6/J female mice were immunized *s.c.* with 100 µg e1 peptide (sequence 83–120 of Kir4.1) in Phosphate Buffer Saline (PBS)/IFA emulsion into the flanks (100 µl divided equally), followed by two more weekly e1 (50 and 25 µg) injections under ketamine/xylasine anaesthesia. For controls, the peptide solution was replaced with saline. One month after, all mice were challenged *s.c.* with 100 µl of PBS/CFA emulsion, followed immediately and 48 h after by 300 ng *i.p.* pertussis toxin injections. Mice were scored daily for typical experimental autoimmune encephalomyelitis (EAE) signs according to the standard 0–5 paralysis (typical EAE) scale with an additional score +1 added when atypical EAE was observed (see extended methods for details in Supplementary material). At the end of the experiment, mice were anesthetized and perfused with 4% paraformaldehyde in sodium phosphate buffer. The spinal cords and brains were post-fixed and sucrose-protected at 4°C, frozen in dry ice and stored at −80°C until cutting. In order to measure anti-e1 serum IgG, blood sampling was performed at the sinus at 1 and 2 months after the first boot. A pool of sera from five e1-immunized mice was kept for immunofluorescence and Western blots. Mouse experimentation was done in accordance with the French legislation and European Treaty ETS 123 after approval by the regional Animal Ethics Committee (CEEA.2012.205).

### Histoneuropathology in e1-immunized mice

Histological evaluation was done on 14µm-thick sagittal sections of the brain and coronal sections of the cervical, thoracic and lumbar spinal cord. Sections were stained with 4′,6-diamidino-2-phenylindole (DAPI) nuclear staining and Black Sudan to evidence demyelinated foci of inflammation in four to six serial sections per CNS region per mouse. For immunofluorescence, sections were subjected to heat antigen retrieval (HIER) and processed with primary rat or rabbit antibodies to immune cell markers (Supplementary Table 2) and corresponding secondary antibodies (donkey AF594- or AF488-coupled F(ab’)2 anti-mouse, anti-rat or anti-rabbit IgG). NeuroTrace™ red fluorescent Nissl stain was used to examine potential alterations in the Purkinje layer. Sections were stained with DAPI and coverslipped with anti-fading mounting medium before analysis.

### Mouse T-cell proliferation assay and enzyme-linked immunosorbent spot

Two weeks after the last immunization boot of e1 or PBS in IFA, mice were sacrificed with thiopental, and blood was collected by cardiac puncture for seric anti-e1 IgG assay. Spleens were harvested and digested with collagenase D for 20 min at 37°C (Roche). To evaluate T-cell proliferation in response to e1 peptide, dissociated splenocytes were labelled with cell proliferation dye (CPD) eFluor670 and cultured for 3 days in the presence of e1 (20 µM) or vehicle (diméthylsulfoxyde)) only. Cells were then stained with fixable viability dye (FVD) eFluor450 and with fluorescein isothiocyanate-conjugated anti-CD3 and PE-Cy7-conjugated anti-CD4 antibodies, and analysed for the percentages of CPD-low cells/total CD3 + CD4 + cells. Amplified e1-specific memory T cells were assessed for IFN-γ release using the mouse IFN-γ ELISPOT Set (BD Biosciences) according to the manufacturer’s instructions.

### Sera from patients with multiple sclerosis or clinically isolated syndrome

A first cohort of patients with multiple sclerosis diagnosed at the multiple sclerosis centre of the Nantes University Hospital consisted of 179 multiple sclerosis patients with disease duration of 8.8 ± 0.5 years (range: 0.1–28) including 149 with a relapsing–remitting form and 30 with a secondary/primary progressive form. The gender ratio was 115F/64M and the average age of 42 ± 1 years (range: 19–73). Four patients were treated with glatiramer acetate, four with dimethylfumarate, 9 with natalizumab, 9 with an S1P modulator, 13 with mycophenolate mofetil, 13 with teriflunomide and 17 with interferon beta. The sera from 86 healthy volunteers (HV gender ratio, 65F/21M; age, 36 ± 1 years, range: 20–65) collected at the same time were processed simultaneously. The second cohort consisted of patients that presented, at time of blood sampling, a clinically isolated syndrome (CIS), defined as a first mono- or multifocal neurological deficit lasting for more than 24 h and not associated with fever or infection, thus compatible with a first presentation of multiple sclerosis.^32,33^ Clinically isolated syndrome patients from UCLouvain^34^ and from the Nantes Hospital were used. The gender ratio was 70F/15M and the average age of 41 ± 1 years (range: 17–78). None were under treatment at blood sampling. Sera from 41 HV (gender ratio, 34F/7M; age, 38 ± 2 years, range: 25–62) received at the same time were processed simultaneously. All sera were coded, alternating HV and patients to allow an appropriate repartition for serum testing in each ELISA plate. Sera were coded and kept at −20°C until use. All donors from the Nantes Hospital provided informed consent in compliance with the Declaration of Helsinki and our local hospital ethical committee. Samples from the Cliniques Universitaires Saint-Luc (UCLouvain) were obtained in conformity with local regulatory and ethical requirements for the residual use of serum samples collected for routine diagnostic procedures or patient care, without additional requirement for informed consent (ethical approval 2007/10SEP/233).

### Anti-e1 immunoglobulin G enzyme-linked immunosorbent assay

After coating 96-well NuncMaxiSorp™ plates overnight at 4°C with e1 peptide diluted at 10 µg/ml in 25 mM borate buffer (pH 9), wells were blocked with PBS Tween-20 0.05% (PBST) and 5% BSA for 2 h at 37°C, incubated with mouse (1:500–1:2000) or human (1:2000) sera in PBST and 0.1% BSA for 2 h at 37°C. After three PBST washes, the corresponding secondary antibody coupled to HRP (1:2000; Jackson ImmunoResearch) was incubated for 1 h at 37°C. TMB substrate (Sigma Aldrich) was added, and the reaction was stopped by adding 50 µl H_2_SO_4_ 0.5 M. Optical density was read at 450 nm using Spark10M multimode microplate reader (Tecan). For human samples, sera were processed in duplicate with 2–3 plates per series. In each plate, eight serial dilutions of a pooled serum internal control from e1-immunized mice and of a human serum internal control (pooled from three multiple sclerosis samples) served as positive day-to-day and plate-to-plate controls. Human specimens were considered high responders (HR) when the optical density exceeded the cutoff value, set at 2.5 SD above the HV mean.

### Inwardly rectifying potassium channel 4.1 immunohistofluorescence

Cryostat sections prepared from 4% paraformaldehyde-perfused control or MOG35-55-induced EAE female mice were subjected to heat-induced epitope retrieval (HIER) and incubated overnight at 4°C with anti-e1 serum and rabbit anti-Cter Kir4.1_356-375_ antibody, revealed by AF594-coupled and AF488-coupled secondary antibodies, respectively. Sections were stained with DAPI and coverslipped with anti-fading mounting medium, and pictures were taken at fixed fluorescence exposure. Peptide-*N*-glycosidase F (PNGase F, New England BioLabs) was used to evaluate the effect of N-linked glycosylation on the anti-e1 reactivities. For this, HIER-treated sections were incubated with 5 U/µl of PNGase F in 10 mM PBS, 10 mM EDTA at pH 7.6 at 37°C overnight, before being processed for immunohistofluorescence.

For fresh-frozen human tissues, 12µm-thick cryostat sections enriched in subcortical WM (Supplementary Table 3) were prepared from selected blocks containing inflamed subcortical WM or NAWM,^35^ defined from CD68 and Luxol Fast Blue stainings (Supplementary Table 4). Acetone-fixed sections were processed for Kir4.1 immunostaining as described above, and incubated with 0.1% Black Soudan to stain the white matter and hide lipofuscin-driven autofluorescence before covering with anti-fade mounting medium. For quantification, three fields at ×40 objective of subcortical WM per sample were acquired at fixed fluorescence exposure time, and the average level of Kir4.1 immunofluorescence was measured with ImageJ software. Sections from four human renal fresh-frozen biopsies were also used for Kir4.1 immunofluorescence: control cortical pre-implant biopsies from two donors and cortical kidney biopsies with chronic inflammation from two patients with interstitial fibrosis/tubular atrophy.

### Cell cultures and tunicamycin treatment

The human U-251 astrocytoma cell line (U-251MG, RRID:CVCL 0021) was cultured in DMEM high glucose medium with 10% heat-inactivated foetal bovine serum (FBS) and penicillin–streptomycin. Cells seeded on Nunc Lab-TekII Chamber Slide™ were treated overnight with 1 µg/ml tunicamycin (Sigma) or vehicle before being fixed 10 min with 2% paraformaldehyde in PBS and further processed for immunohistofluorescence with rabbit anti-Cter Kir4.1_356-375_ and mouse or human HR anti-e1 serum followed by the donkey AF488-coupled F(ab’)2 anti-rabbit IgG and AF594-coupled F(ab’)2 anti-mouse or anti-human IgGs. For competition experiments (with e1-preadsorbed sera), mouse (1:100) or HR (1:50) anti-e1 sera were incubated 36 h at 4°C with e1 peptide at 20 µg/ml in PBS supplemented with 2% BSA, centrifuged (14 000 g) for 30 min to remove antibody complexes before being applied to tissue sections at desired concentration. Slides were coverslipped with Prolong Gold DAPI mounting medium before taking pictures at ×40 objective under the fluorescent microscope at fixed exposure time.

### Western blot

For CNS protein extracts, 30–60 mg of tissue per sample or cultured cells were processed for crude membrane extraction as previously described.^36^ For mouse samples, archival MOG_35-35_ EAE (score 3; scale 0–5) or control mouse spinal cords kept at −80°C were used.^37^ For human brain, crude membrane preparations from enriched WM samples (Supplementary Table 5) were lysed in RIPA buffer. For PNGase treatment, samples (15 µg aliquots) were subjected to *N*-deglycosylation following the provider’s instructions. RIPA lysates were heated in LDS buffer (ThermoFisher) at 70°C for 10 min in the presence or absence of 1 mM DTT before loading a total of 10–15 µg protein in each lane with a lane for the dual colour molecular marker (BioRad). Samples were subjected to electrophoresis using 4–12% NuPage BisTris gels, electrotransferred to polyvinylidene difluoride membranes. Western blotting was performed with the following antibodies revealed using the SuperSignal Pico (or Femto if specified) West reagents (Pierce): rabbit anti-Kir4.1_356-375_ or anti-Kir4.1_93-106_ antibodies (1:2500, Alomone labs); mouse anti-e1 serum (1:800) or human HR serum (1:200); rabbit anti-human ATP1A1 (1:5000, 55187-1-AP, Peprotech) and corresponding peroxidase-conjugated donkey anti-mouse, anti-human or anti-rabbit IgGs (JIR, 1:20 000). Bands were quantified when needed using Image J.

### Homology 3D modelling

Human Kir4.1 tetramer 3D model was generated by the Swiss-Model protein structure homology-modelling server (https://swissmodel.expasy.org; licence available at https://creativecommons.org/licenses/by-sa/4.0/legalcode), using the crystal structure of the prokaryotic Kir3.1 (SMTL ID: 2WLL) and cytoplasmic domain of Kir3.2 (SMTL ID: 3AUW) as templates. Refinement of the Kir4.1 e1 sequence 3D model was generated using the crystal structure of Kir2.2 (SMTL ID: 3SPC) as template; the calculated percentage of identity on the aligned sequences was 40%. Molecular visualization was performed with RasMol and Raster3D softwares.

### Immunofluorescence experiments with transfected Chinese hamster ovary cells

Chinese hamster ovary cells (CHO-K1, RRID:CVCL_0214) were cultured in DMEM high glucose, 10% FBS and antibiotics. Cells were transiently transfected using lipofectamine 2000 and pcDNA3.1 containing the human Kir4.1 sequence under control of the human cytomegalovirus promoter (gift from Dr. J. Devaux, IGF CNRS-Université de Montpellier, France). N104Q Kir4.1 mutant plasmid was generated with Quikchange II XL site-directed mutation kit (Agilent), and Kir4.1 sequences were checked by Eurofins Genomics. After 36–48 h transfection, EDTA-dissociated cells were fixed with 2% formaldehyde in PBS for 10 min. After dissociation in PES buffer (PBS, 2 mM EDTA, 2% FBS), cells were incubated in a 96-well plate (3–4 × 10^5^/well) overnight at 4°C with PES containing 0.002% Triton-X100, rabbit anti-Kir4.1_356-375_ (1:2000) and mouse anti-e1 serum (1:400) or no primary antibody (background control), followed by washes and incubation with donkey AF488-coupled anti-rabbit and AF594-coupled anti-mouse IgGs (1:800). After washing, cells were cytospined with Shandon Cytocentrifuge (Thermo Scientific) on Superfrost slides and coverslipped with Prolong Gold DAPI mounting medium before analysis under the fluorescent microscope. For each group (non-transfected, WT or N104Q Kir4.1 transfected), slides were coded and DAPI + cells (1700, 2136 and 1569 in total, respectively) were analysed by counting positive cells for Cter- and e1-immunofluorescence with the minimal fixed exposure time, set to avoid detection of lower fluorescent signals in the non-transfected group.

### Statistical analysis

Statistical analysis was performed with GraphPad Prism 8. The Fisher’s exact test was used to compare the proportion of patients with high anti-Kir4.1 IgG levels with those of control subjects. Normality tests were performed on all other samples with Shapiro–Wilk test. If normality was assumed, parametric analysis was used (ANOVA followed by Dunn *post hoc* analysis, or two-tailed Student’s *t*-test with Welsh correction when required). When normality could not be assumed (for data presented in Supplementary figures), we used non-parametric tests (repeated measures of Friedman test and Kruskal–Wallis followed by Dunn *post hoc* test). In all tests, *P (for overall ANOVA)* and *P* (for *post hoc* or *t*-test analysis) values of <0.05 were considered statistically significant. All data are presented as mean ± SEM.

## Results

### Immunization with e1 peptide promotes encephalomyelitis after blood brain barrier challenge

**Figure 1:**
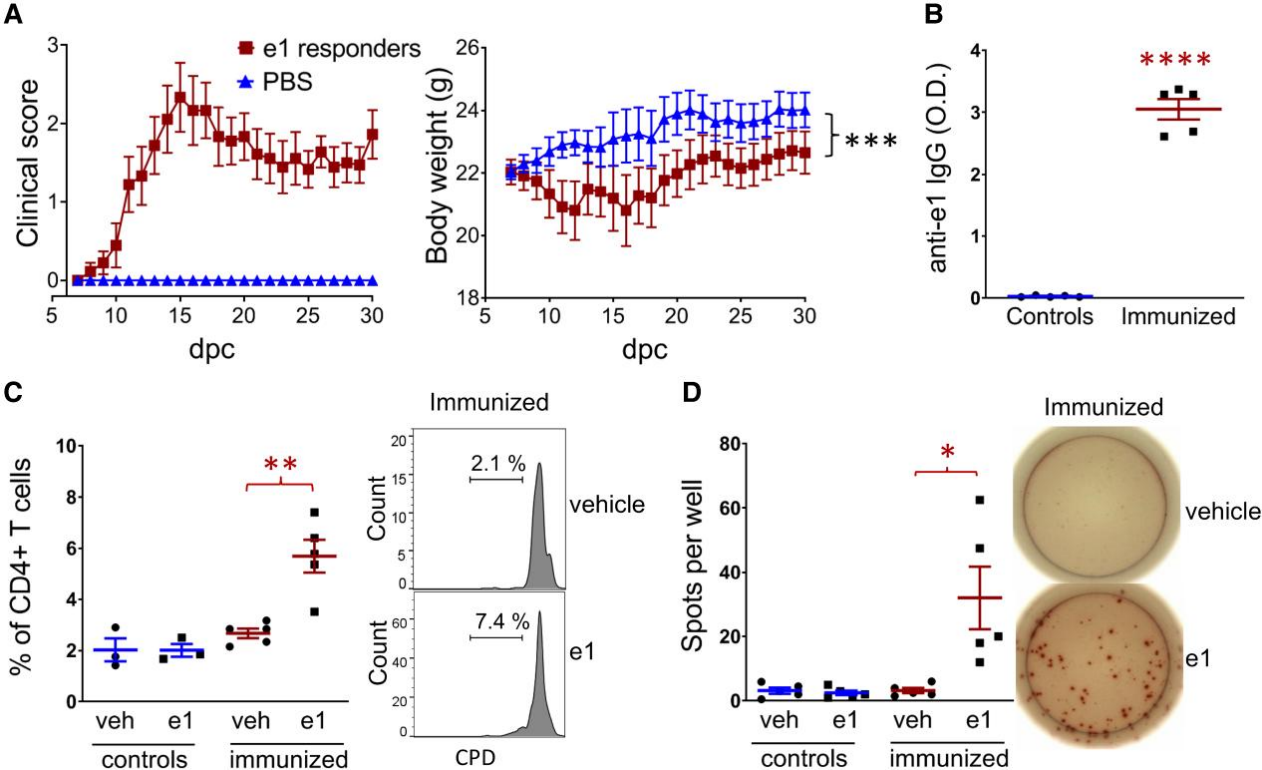
**Immunization with Kir4.1 e1 peptide promotes EAE after BBB challenge.** (**A**) Clinical score of typical/atypical EAE that developed in nine of 23 female e1(aa 83–120)-immunized mice after CFA/PTX challenge and corresponding body weight as compared to 13 PBS control female mice. Repeated measured ANOVA (group effect, *F* = 125.3, *P* < 0.0001; Dunnet’s *post hoc* analysis, overall difference between the nine e1-responders and PBS control mice: ***, *P* < 0.001. dpc, day post-challenge. (**B**) Representative serum anti-e1 IgG levels (mice used for the splenocyte assays, *n* = 5/group). Unpaired *t*-test with Welch’s correction: ****, *P* < 0.0001 (*t* = 18.35, *df* = 4.012). **(C)** CD4+ T-cell proliferation assay from splenocytes of three controls or five e1-immunized mice and representative flow-cytometry histograms showing the increased percentage of proliferating CD4+ T cells after *in vitro* stimulation with e1 peptide versus vehicle (veh) from five e1-immunized mice. Paired *t*-test: **, *P* < 0.01 (*t* = 4.889, *df* = 4). Peptide stimulation was ineffective in control mice (*n* = 3). CPD, cell proliferation dye. **(D)** IFN-γ assay and representative ELISPOT images showing the increased number of IFN-γ producing cells per well after *in vitro* stimulation with e1 peptide from five e1-immunized mice. Paired *t*-test: *, *P* < 0.05 (*t* = 3,131, *df* = 4). Peptide stimulation was ineffective in control mice (*n* = 5).

To establish a model of CNS autoimmmune attack by Kir4.1 autoreactivity, we initially tested a single immunization with a pool of seven Kir4.1-specific peptides (including e1_83-120_) with complete Freund Adjuvant (CFA) in C57Bl/6 mice. This was insufficient to trigger EAE, but when the recipients of the Kir4.1 peptide pool were co-immunized with 50 µg MOG_35-55_ that induces mild relapsing–remitting EAE, we observed that these animals later developed significantly more severe symptoms between 1 and 2 months post-immunization as compared to animals immunized with MOG_35-55_ alone (Supplementary Fig. 1). This suggested that a delayed Kir4.1 autoantibody response was responsible for the increased deficits. To increase anti-e1 antibody production, mice were vaccinated with three boluses of e1 peptide in PBS/IFA. The e1-immunized mice did not develop neurologic symptoms. One month after the first bolus, the CFA/pertussis toxin (PTX) challenge was used to alter the blood brain barrier (BBB) permeability, as previously reported.^38,39^ In this context, one-third of the e1-immunized mice develop clinical signs of encephalomyelitis (Fig. 1A), starting with well-known symptoms of typical EAE (7/23 mice, mean day of onset: 10.3 ± 0.5) and further signs of atypical EAE (9/23 mice, mean day of onset: 22.6 ± 2.4). No epileptic symptoms (e.g. jerky movements and convulsive seizures) were noted. Analysis of proteinuria with a dipstick test on urine collected immediately before sacrifice showed normal levels below 0.3 g/l (five symptomatic e1-immunized mice), indicating the absence of major kidney damage. All e1-immunized mice studied developed anti-e1 IgG responses (Fig. 1B, Supplementary Fig. 2). Analysis of splenocytes from five control and five e1-immunized mice revealed the development of antigen-specific CD4 T-cell responses to e1 peptide in e1-immunized mice (Fig. 1C and D).

### Central nervous system neuroinflammation in symptomatic e1-immunized mice

**Figure 2:**
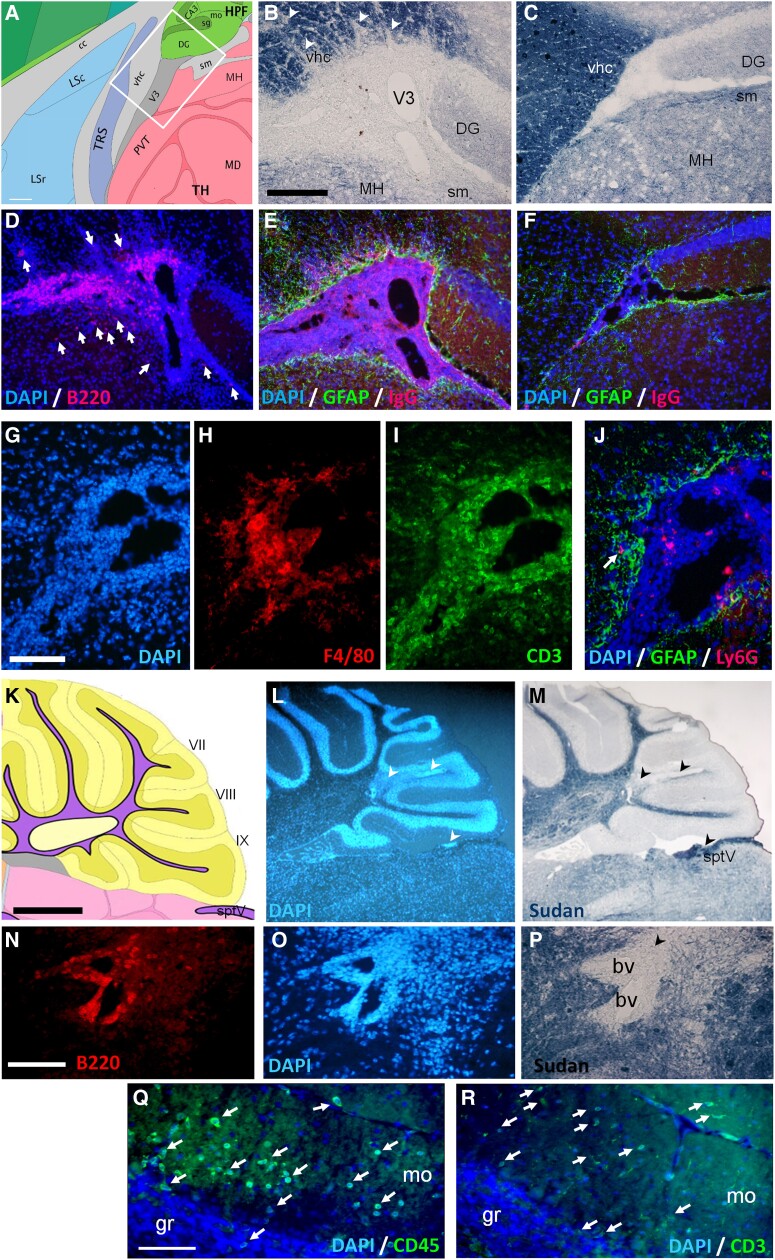
**Demyelination and inflammation in the brain of e1-immunized female mice.** (**A**) Schematic forebrain area (white square) used for **B**–**J**. Mouse atlas reference image 19, P56, sagittal (obtained from the Allen Institute website at www.alleninstitute.org); scale bar, 180 µm. (**B**–**C**) Black Sudan myelin stain showing patches of demyelination (arrowheads) of a symptomatic mouse (**B**) versus a control mouse (**C**). **(D)** DAPI stain (blue) with B220-immunoreactivity (red) showing periventricular B cell enrichment in the leptomeninges of a symptomatic mouse. Few B220 + cells also invade the bordering thalamus and stria medullaris (arrows). (**E**–**F**) DAPI stain with GFAP-immunoreactivity (green) and IgG-immunoreactivity (red) from a symptomatic mouse (**E**) versus limited IgG detection in a control mouse (**F**). Scale bar **B**–**F**, 180 µm. (**G**–**I**) DAPI stain (**G**) with F4/80-immunoreactivity (**H**) and CD3-immunoreactivity (**I**) from a symptomatic mouse in the V3 area. (**J)** DAPI stain and Ly6G-immunoreactivity (red) showing limited granulocyte infiltration (arrow) in the GFAP-immunoreactive (green) parenchyma. Scale bar **G**–**J**, 80 µm. (**K**) Schematic cerebellar area used for **L**–**R**. Reference Atlas image 16, P56, sagittal. (**L**–**M**) DAPI (**L**) and myelin (**M**) stains showing lobule demyelination (arrowheads) of a symptomatic mouse. Scale bar **K**–**M**, 800 µm. (**N**–**P)** White matter perivascular infiltration, magnification fields with B220-immunoreactivity (**N**), DAPI (**O**) and myelin (**P**) stains. Scale bar **N**–**P**, 80 µm. (**Q**–**R**) Immune cell infiltration (arrows) in cerebellar molecular layer revealed by (**Q**) DAPI/CD45-immunoreactivity (green) or (**R**) DAPI/CD3-immunoreactivity (green). Scale bar **Q**–**R**, 80 µm. Legends: bv, blood vessels; DG, dentate gyrus; gr, granule layer; lfu, lateral funiculus; m, meninges; MH, medial habenula; mo, molecular layer; pcl, Purkinje neuron layer; sm, stria medullaris; sptV, spinal trigeminal tract; vhc, ventral hippocampal commissure; V3, third ventricle. See Supplementary material file for corresponding separate channel images when required.

**Figure 3:**
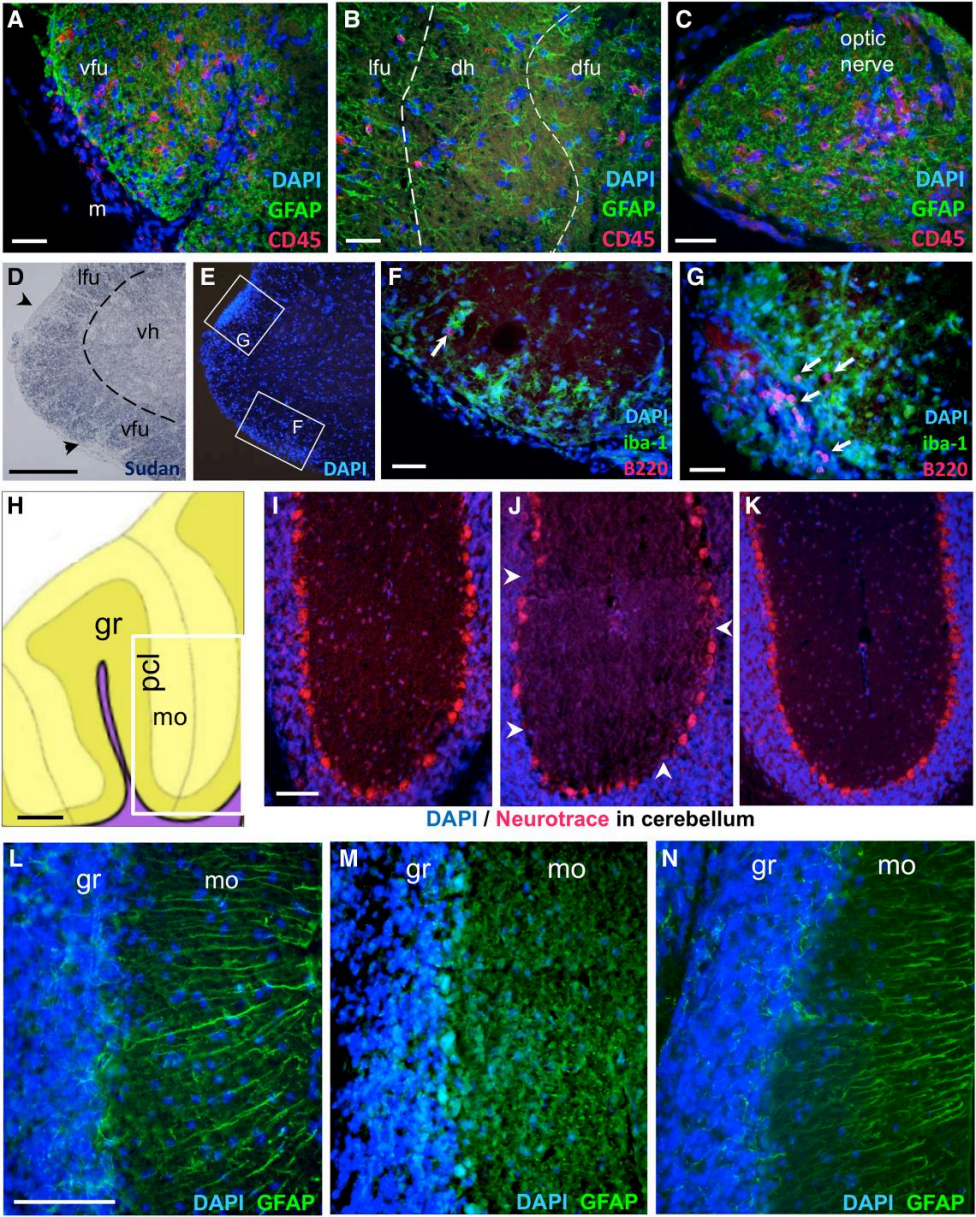
**Neuroinflammation in the spinal cord, optic nerve and cerebellum in e1-immunized female mice.** (**A**–**G**): Immune cell infiltration in the spinal cord parenchyma (**A**–**B**) or optic nerve (**C**) revealed by CD45-immunoreactivity (red), GFAP-immunoreactivity (green) and DAPI (blue). The dashed lines separate the grey and white matter in B. Scale bar **A**–**C**, 40 µm. (**D**–**E**) Myelin (**D**) and DAPI (**E**) stains showing spots (arrowheads) of perimeningeal demyelination and cell infiltration. Scale bar **D**–**E**, 200 µm. (**F-G**) B220-immunoreactivity (red) and iba-1-immunoreactivity (green) showing limited B cell (arrows) and wider macrophage parenchymal infiltration. Scale bar **F**–**G**, 40 µm. Legends: dfu, dorsal funiculus; dh, dorsal horn; lfu, lateral funiculus; m, meninges; vfu, ventral funiculus; vh, ventral horn. **(H**–**N):** Alterations in Purkinje cell layer and Bergman fibre integrity in symptomatic e1-immunized mice: **(H**) Schematic view of a sagittal section of the cerebellum (yellow) used for photograph fields (white box) in **I**–**K**. Scale bar, 200 µm. Legends: gr, granule layer; mo, molecular layer; pcl, Purkinje neuron layer; VIII, pyramus lobule. **(I**–**K)** DAPI (blue)/Neurotrace (red) staining from a control mouse (**I**), closed to a dense immune cell infiltrate in the white matter from a symptomatic mouse (**J**), or from a symptomatic mouse without neuroinflammation in this area (**K**). Arrowheads indicate Purkinje cell layer disruption. Scale bar for **I**–**K**, 80 µm. **(L**–**N)** Bergman fibres stained for GFAP (green) and DAPI stain (blue) from a control mouse (**L**), close to a dense immune cell infiltrate in the white matter from a symptomatic mouse (**M**), or from the same mouse but within an unaffected lobe (**N**). Scale bar for **L**–**N**, 80 µm. See Supplementary material file for corresponding separate channel images when required.

Antibodies against immune cell markers and GFAP with Soudan Black stain were used to assess immune cell infiltration in the CNS parenchyma and demyelination in five e1-immunized mice showing typical and atypical EAE signs. CD45-immunoreactivity (IR) confirmed the lack of immune cell parenchymal infiltrates in two non-symptomatic e1-immunized mice and three IFA/CFA/PBS control mice (not shown). In symptomatic mice, the most striking observation was the presence of CNS B cells in forebrain leptomeninges at the border of demyelinated WM tracts and in perivascular cuffs in demyelinated cerebellar WM; these B cells, as Ly6G + cells (granulocytes) did not invade deeply the parenchyma (Fig. 2A–J). Parenchymal inflammatory foci were however enriched in CD3+ T cells and macrophages/activated microglia (F4/80 + or Iba+). Scarce T cells were also found in the WM tracts such as corpus callosum, fimbria, stria terminalis, ventral hippocampal commissure or spinal trigeminal tract in the brainstem. Moreover, some small foci of perivascular T cells were observed in the grey matter such as in the striatum, and isolated T cells could be observed in the cortical grey matter, such as the somatomotor and occipital cortices (Supplementary Fig. 3B, D–F). Close to demyelinated *arbor vitae cerebelli* (Fig. 2K–R), isolated T cells were found in the molecular layer of the cerebellum (mo) with scarce immune cells in the granule layer (gr, Fig. 2R). In the spinal cord (Fig. 3A and B, D–G), perimeningeal patches of demyelination (Fig. 3D) or immune cell infiltration (Fig. 3E–G) were observed all along the rostro–caudal axis in the five symptomatic mice tested, with B-cell clusters mostly restricted to leptomeninges and—as in the brain—with only few B cells invading the nearby WM. The optic nerves presented variable immune cell infiltration, ranging from 1 to 50 CD45 + immune cells per section (Fig. 3C) with scarce parenchymal B cells. Moreover, as numerous T cells infiltrated the molecular layer of two mice that exhibited severe cerebellar demyelination, we examined the integrity of the underlaying Purkinje cell monolayer using Neurotrace (Fig. 3H–K) and of Kir4.1-expressing Bergman glia, that form a strictly arranged system of parallel GFAP-labelled fibres (Fig. 3L) important for Purkinje cell function and survival.^16^ Just above the inflammatory cuffs in the *arbor vitae*, the Purkinje layer was indeed discontinued (Fig. 3J) with an estimated 30% reduction in cell number, and the Bergman glial fibres were disrupted with GFAP patchy-like staining (Fig. 3M), in contrast to lobules from control mice (Fig. 3I and L) or to distant lobules from the same mice (Fig. 3K and N).

### A subset of multiple sclerosis patients exhibits high anti-e1 seric immunoglobulin G levels

**Figure 4:**
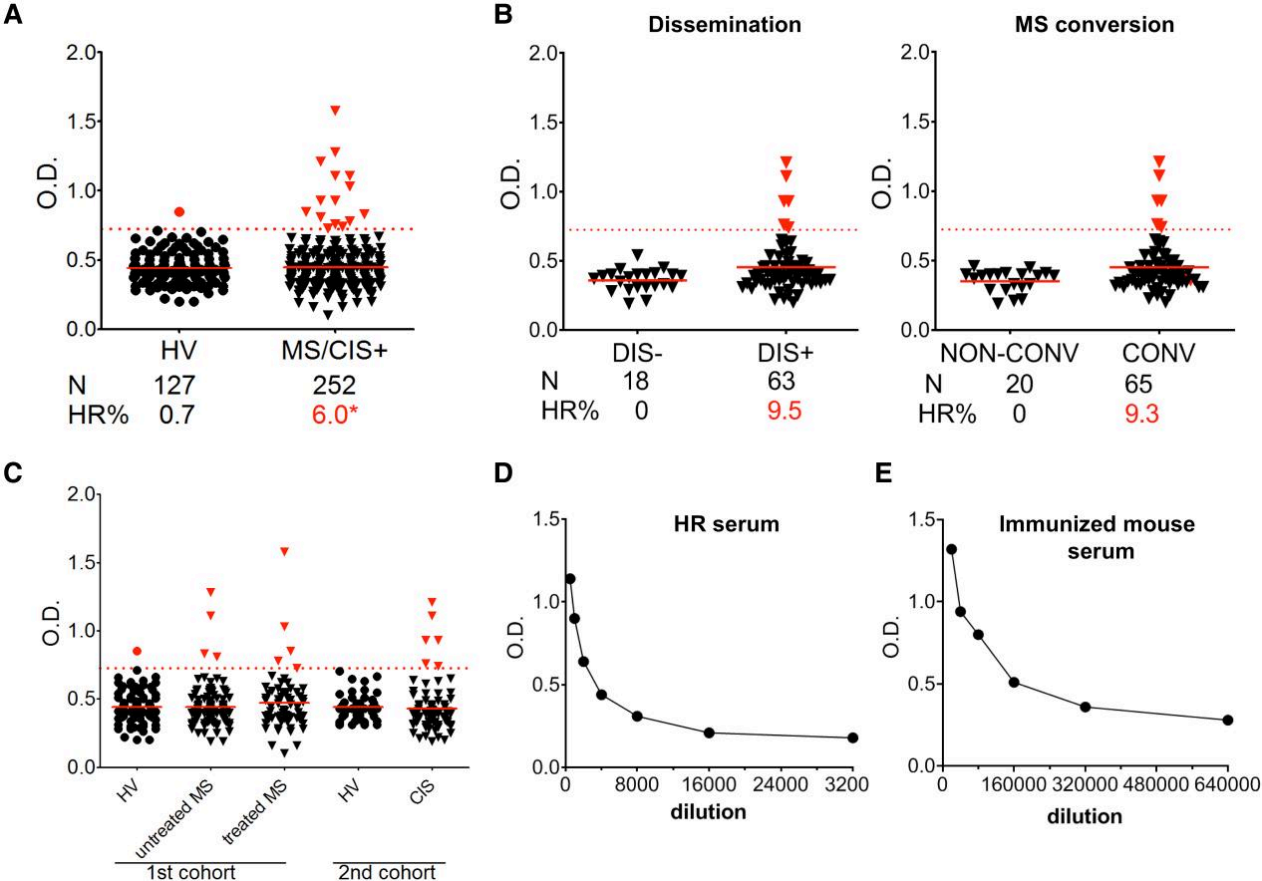
**High Kir4.1 anti-e1 IgG reactivity in a subset of multiple sclerosis/CIS patients.** According to the new criteria,^40^ we examined whether CIS patients were presenting lesion dissemination in space at time of blood sampling (group DIS+) or not (group DIS-), or whether they developed a second clinical attack within 6 months (multiple sclerosis conversion, CONV group) or not (non-CONV group). Information for dissemination was not available for four patients. The CIS patients that were DIS + and/or CONV (CIS + group, *n* = 73), representing a first episode of multiple sclerosis (MS), were added to the first multiple sclerosis cohort to calculate the % of HR patients in multiple sclerosis. (**A**) Comparison of anti-e1 IgG reactivity in sera from multiple sclerosis/CIS + patients versus healthy volunteers (HV). Dashed red line, threshold level corresponding to the HV mean value plus 2.5 SD. Red points/triangles, values above threshold. Red full lines, mean values. One-sided Fisher’s exact test: *, *P* = 0.015. (**B**) Distribution of HR patients according to the CIS subgroup category. (**C**) Distribution of anti-e1 IgG levels among the different healthy volunteers, multiple sclerosis or CIS groups used in this study. (**D**–**E)** Anti-e1 IgG ELISA detection using serial dilutions with a pool of three HR sera (**D**) or with a pool of serum from 8 weeks e1-immunized mice (**E**) processed simultaneously.

Applying a threshold value corresponding to the HV mean value plus 2.5 SD, we revealed that 6% (*n* = 15) of multiple sclerosis and CIS + patients (HR) and only 0.7% (*n* = 1) of HV controls exhibited high levels of anti-Kir4.1(e1) IgGs (Fig. 4A). Of note, three-quarters of the clinically isolated syndrome patients presented lesion dissemination in space (DIS+) at the initial event or developed multiple sclerosis clinical signs within 6 months after (CONV); strikingly, all 6 HR clinically isolated syndrome patients were distributed within these subgroups (Fig. 4B; Supplementary Table 1). Even for the CIS + group, representing early multiple sclerosis, we found a moderate proportion (9.3–9.5%) of patients exhibiting high anti-e1 IgGs. High responders patients were found within untreated as treated multiple sclerosis patients (Fig. 4C). The pool of HR sera, constituted for immunoassays, exhibit anti-e1 IgG titre estimated 20× lower than in the pooled serum from e1-immunized mice (Fig. 4D and E).

### Increased e1-immunoreactivity in mouse central nervous system during neuroinflammation

**Figure 5:**
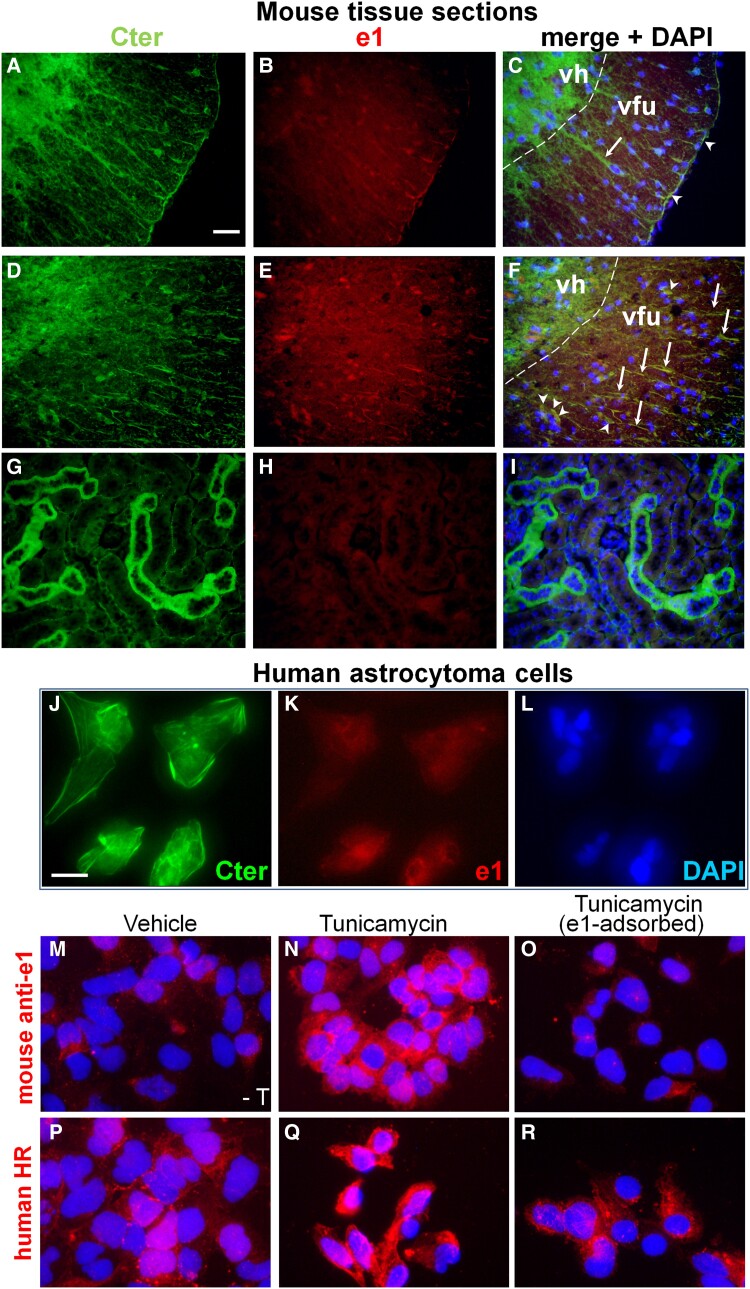
**Increased e1 immunoreactivity in MOG_35-55_-induced EAE and astrocytoma cells. (A**–**F):** Spinal cord sections from control or MOG_35-55_-induced EAE paraformaldehyde-perfused female mice. First column, anti-Cter Kir4.1 immunoreactivity. Second column, immunoreactivity with the mouse anti-e1 serum. Third column, corresponding merge fields with DAPI stain. (**A**–**C**) In control mouse spinal cord, few astrocytic radial fibres were immunoreactive with the commercial antibody but not labelled with anti-e1 serum (arrows) whereas few pial fibres were stained with both anti-Cter and anti-e1 serum (arrowheads). (**D**–**F**) In the WM of the inflamed spinal cord of female mice with MOG_35-55_-induced EAE, numerous radial fibres immunoreactive with anti-Cter (arrows) and cells with oligodendrial morphology (arrowheads) are immunoreactive with the anti-e1 serum. Dashed line indicates the boundary between ventral horn (vh) and ventral funiculus (vfu). In the grey matter, diffuse Cter immunoreactivity corresponding to astroglial network is observed while only a non-specific staining is observed on few neuronal nuclei with the anti-e1 serum. When primary rabbit or mouse antibodies were omitted, no green or red signal was seen in white matter at the exposure time used (not shown). **(G**–**I**) On kidney sections from a control paraformaldehyde-perfused mouse, tubules are highly labelled with the Cter antibody (**G**) but not with the mouse anti-e1 serum (**H**). Scale bar for **A**–**I**, 40 µm. **(J**–**R**): Kir4.1 immunoreactivities in U-251 astrocytoma cells. **(J**–**L)** Representative immunostainings of untreated cells showing anti-Cter Kir4.1 immunoreactivity (**J**) versus anti-e1 reactivity (**K**) and corresponding DAPI stain (**L**). **(M**–**O)** Immunoreactivity obtained with mouse anti-e1 serum (red) and DAPI stain in untreated (**M**) versus tunicamycin-treated (**N)** cells, and with e1-preadsorbed mouse antiserum on tunicamycin-treated cells (**O**). **(P**–**R)** Immunoreactivity obtained with human HR serum (red) and DAPI stain in untreated (**P**) or tunicamycin-treated (**Q**) Cells, and with e1-preadsorbed mouse antiserum on tunicamycin-treated cells (**R**). Scale bars **A**–**I**, **J**–**R**: 50 µm.

We compared Kir4.1-immunoreactivity obtained with the Cter (Kir4.1_356- 375_) antibody versus our mouse anti-e1 serum on archival sections from paraformaldehyde-fixed spinal cords of control and classic MOG_35–55_-induced EAE mice.^41^ In controls, a strong diffuse Cter-immunoreactivity was detected over the entire grey matter as reported previously^42^ but not with the mouse anti-e1 serum; astrocytic radial fibres were also detected with the C-ter antibody but not with the anti-e1 serum, whereas few pial endfeet could be detected with both the rabbit anti-Cter and the mouse anti-e1 serum (Fig. 5A–C). In white matter of the inflamed (EAE) spinal cord, numerous Cter-IR radial fibres were also labelled using the anti-e1 serum, as cells with oligodendroglial morphology (Fig. 5D–F). In mouse kidney, cortical tubules were highly labelled with the Cter antibody but not with the mouse anti-e1 serum (Fig. 5G–I), suggesting that posttranslational modifications such as the high *N*-glycosylation of Kir4.1 reported in this tissue^28,29^ prevents recognition by the anti-e1 serum. In the CNS, e1-immunoreactivity was also barely detected on Bergman glial fibres, astrocytes or oligodendrocytes of WM tracts, layer I cortical astrocytes (data not shown) and cortical perivascular endfeet or hippocampal stellate astrocytes in contrast to the strong Cter-immunoreactivity. However, treatment of sections with PNGase F resulted in increased e1-immunoreactivity on cortical microvessel astrocytic endfeet and hippocampal stellate astrocytes (Supplementary Fig. 4) suggesting that a higher e1 glycosylation status in these cell populations hampered detection by the mouse anti-e1 serum.

### Increased e1-immunoreactity in astrocytoma cells after tunicamycin treatment

Western blot analysis of crude membrane preparations from U-251 astrocytoma cells revealed major bands between 42–45 and 50 kDa with the rabbit anti-Cter antibody, whereas the human HR serum and the mouse anti-e1 serum failed to give significant signal (data not shown) suggesting that anti-e1 antibodies contained in these sera poorly recognize the linear (denatured and reduced) forms (glycosylated or not) of Kir4.1. We thus examined e1-immunoreactivity on fixed U-251 astrocytoma cells that were treated or not with tunicamycin, an inhibitor of *N*-glycosylation. Using the Cter antibody, we confirmed that this cell line highly expresses Kir4.1 at the cell surface and intracellularly, whereas the mouse anti-e1 serum only gave a faint signal around the nuclei of these cells (Fig. 5J–L). Tunicamycin treatment resulted in increased e1-immunoreactivity that was abolished by preadsorbtion of the mouse serum with e1 peptide (Fig. 5M–O). Similarly, the HR serum gave only punctate signals in the cytoplasm of untreated U-251 cells, and tunicamycin treatment resulted in increased intracellular immunoreactivity that was inhibited by e1-preadsorbtion of the serum (Fig. 5P–R). Taken together, these data showed that the anti-e1 mouse and HR sera preferentially bind to the aglycosylated e1 domain of Kir4.1.

### Increased glial e1 inwardly rectifying potassium channel 4.1 immunoreactivity in multiple sclerosis white matter

**Figure 6:**
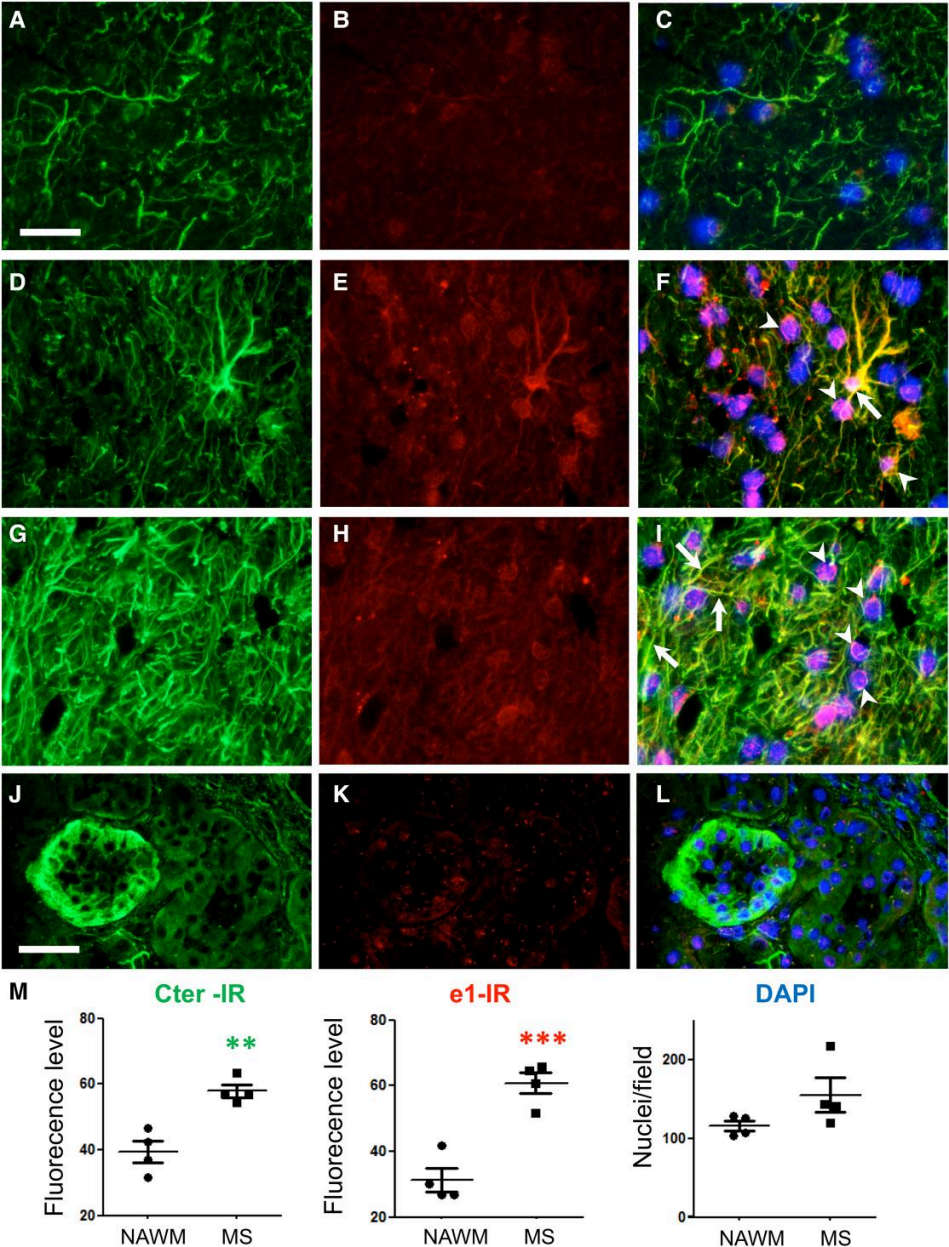
**Increased Kir4.1 immunoreactivity in multiple sclerosis brain.** First column, Cter Kir4-1 immunoreactivity (green). Second column, immunoreactivity with mouse anti-e1 serum (red). Representative photographs obtained from the different samples in the green or red channel were taken at the same exposure time. Third column, merged signals with DAPI stain and with enhanced red channel contrast to better visualize co-localization. (**A**–**C**) Subcortical white matter from a control brain. Kir4.1 is mainly detected on glial fibres using anti-Cter antibody whereas almost no signal is detected with the mouse anti-e1 serum. (**D**–**F**) Subcortical white matter active lesion with increased Kir4.1 immunoreactivity in glial fibres. An immunoreactive signal on glial cells with astrocytic morphology (arrow) and around the nuclei of oligodendrocyte-like cells (arrowheads) is also detected with the mouse anti-e1 serum. (**G**–**I)** Another subcortical white matter active lesion with high Kir4.1 immunoreactivity on glial fibres. Immunoreactive signals on glial fibres (arrows) and around the nuclei of oligodendrocyte-like cells (arrowheads) are also shown with mouse anti-e1 serum. Scale bar **A**–**I**, 40 µm. (**J**–**L**) Cortical kidney from a patient with chronic tubule interstitial nephritis. Kir4.1 expression by a distal tubule is well detected by the Cter antibody whereas the anti-e1 mouse serum gives barely detectable signal in the same tubule. Scale bar **J**–**L**, 40 µm. (**M**) Quantification of immunofluorescence signals from four different NAWM samples (circles) versus four different active white matter lesions (squares) from postmortem subcortical tissues. Two-tailed unpaired *t*-test: **, *P* < 0.01 (*t* = 4.896, *df* = 6); ***, *P* < 0.001 (*t* = 6.134, *df* = 6).

We further determined whether e1-immunoreactivity differs from Cter-immunoreactivity in human control and multiple sclerosis brains. In control samples, Cter-labelling was well identified and homogenously distributed on glial fibres. However, the immunolabelling of the glial fibres with the mouse anti-e1 serum was faint, and only few oligodendrocyte-like cells were weakly labelled (with a ring-shaped pattern above the nucleus) by this antibody as for scarce astrocytes (Fig. 6A–C, Supplementary Fig. 5). In multiple sclerosis (Fig. 6D–I), increased Cter-immunoreactivity was observed on glial fibres in active WM lesions as compared to normal appearing white matter (NAWM). The mouse anti-e1 serum-stained oligodendrocyte-like cells and revealed ring-shaped and fibre-like labelling. Several strongly labelled WM astrocytes were also evidenced. The quantification of Cter or anti-e1 fluorescence intensities in multiple sclerosis lesions versus controls indicated a 1.5-fold and a 2-fold increase, respectively (Fig. 6M). To determine whether inflammation in the periphery increases similarly anti-e1 reactivity, we analysed two specimens of kidney cortices obtained by biopsy from humans affected by chronic tubule interstitial nephritis versus two healthy preimplant controls. Anti-Cter Kir4.1 antibodies clearly labelled a subset of tubules, whereas the mouse anti-e1 serum did not stain these structures (Fig. 6J–L). Taken together, these results suggest that inflammation increases anti-e1 reactivity in the CNS but not in the peripheral tissues such as kidney.

### Central nervous system inwardly rectifying potassium channel 4.1, including its shorter form, is upregulated during neuroinflammation

**Figure 7:**
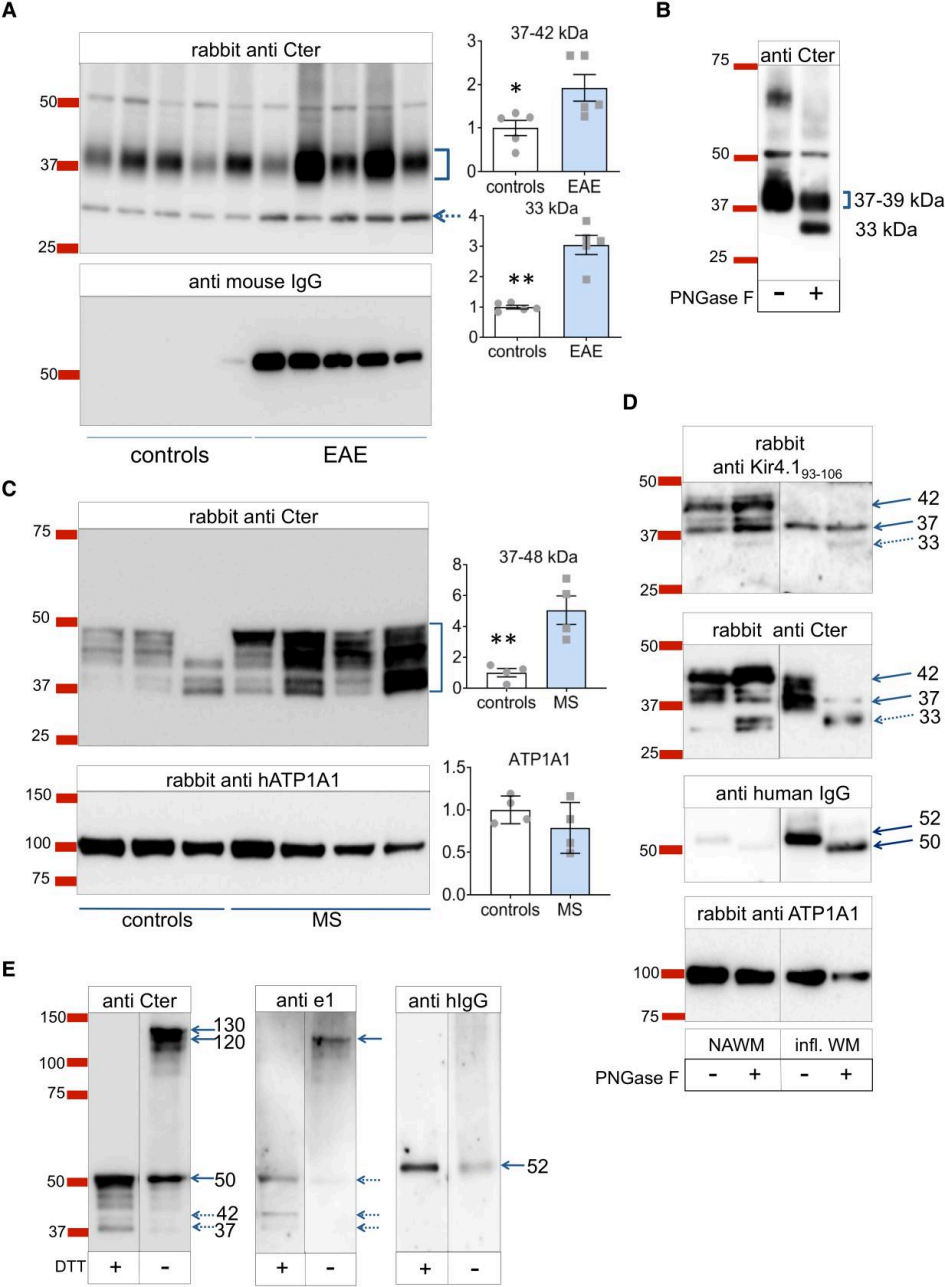
**Kir4.1 molecular forms revealed by Western blot.** (**A**) Kir4.1 detection in reduced denaturing conditions with spinal cord membrane extracts from MOG_35-55_ EAE mice or their controls. Top: anti-Cter detection (overnight primary antibody incubation, 15 s exposure) with densitometry relative to controls. Two-tailed unpaired *t*-tests, controls (PBS/CFA treated, *n* = 5 mice) versus MOG35-55 induced EAE (*n* = 5 mice): *, *P* < 0.05 (*t* = 2.618, *df* = 8) ; **, *P* < 0.01 (*t* = 6.363, *df* = 4.293 with Welsh correction); Bottom, mouse IgG detection revealing the glycosylated heavy chain subunit (52 kDa, ) in EAE samples. Ea (**B**) WB (reduced denaturing conditions) with mouse spinal cord membrane extracts subjected or not to overnight PNGase digestion (anti-Cter detection after 1 h primary incubation). **(C)** Kir4.1 detection in reduced denaturing conditions using human white matter membranes from controls (*n* = 4 individuals) and WM active multiple sclerosis lesions (MS, *n* = 4 individuals). Top, rabbit anti-Cter. Controls versus MS, two-tailed unpaired *t*-test: **, *P* < 0.01 (*t* = 4.195, *df* = 6); Bottom, rabbit anti-human ATP1A1. (**D**) WB from white matter membrane extracts of a control NAWM versus a highly inflamed sample processed or not for overnight PNGase digestion. Detection with rabbit anti-Kir4.1_93-106_ (femto detection), rabbit anti-Cter Kir4.1_356-375_, anti-human IgG, and rabbit anti-human ATP1A1. Western blot for human IgGs confirmed the high IgG content of the inflamed WM and the deglycosylation effectiveness of the heavy chain from 52 to 50 kDa by PNGase whereas the size of the ATP1A1 band was unaffected. (**E**) Comparison of Kir4.1 detection using reduced (+ DTT) versus unreduced (− DTT) denaturing conditions from a multiple sclerosis sample. The membrane was probed with mouse anti-e1 serum, rabbit anti-Cter antibody, and anti-human IgG. See Supplementary material file for corresponding uncropped blot images.

We first evaluate Kir4.1 expression and molecular weight forms by Western blot using archival fresh-frozen EAE spinal cords from five MOG_35-55_-immunized mice (classical EAE, mean score 3, days post-immunization 16–24) and their corresponding CFA/PBS-treated controls. Using crude membrane extracts, a major band between 37 and 45 kDa was evidenced with the Cter antibody (Fig. 7A, top). Relative quantification indicates a 2-fold increase of Kir4.1 expression at 37–42 kDa and a 3-fold increase at ∼30 kDa in EAE samples. As expected, mouse IgG was highly detected in EAE samples (Fig. 7A, bottom). Total protein labelling on the PVDF membrane with DB71 stain ensured homogeneity of samples (data not shown). Altogether, this suggests an increase in Kir4.1 expression in the inflamed spinal cord, including a smaller ∼30 kDa Kir4.1 form, representing about 10% of Cter Kir4.1 signal. Overnight PNGase treatment of a mouse spinal cord sample decreased the intensity of the bands above 39 kDa and favoured the appearance of the 33 kDa form, likely corresponding to a cleavage fragment of the deglycosylated Kir4.1 monomer (Fig. 7B). Reprobing with the mouse anti-e1 and the HR serum failed to give any significant signal above background (data not shown).

We then examined Kir4.1 expression in human WM active lesions and controls. Kir4.1 detection using the anti-Cter antibody revealed molecular forms between 37 and 48 kDa, that were strongly increased in multiple sclerosis samples (Fig. 7C). We next compared samples from a control subject and from a patient affected by severe acute neuroinflammation, after or without overnight PNGase treatment. Since our mouse anti-e1 serum failed to detect significant Kir4.1 signal in these conditions, we tested a commercial antibody against a shorter (linear sequence 93–106) epitope of the e1 domain. This antibody identified Kir4.1 at 37 and 42 kDa in the control sample, whereas only a 37–38 kDa band was detected in the inflamed white matter which is highly enriched in human IgGs (Fig. 7D). PNGase digestion resulted in the appearance of a 30–33 kDa form at the expense of the 37–42 kDa forms; this was even more noticeable with the inflamed WM. Since the 30–33 kDa band was not detected by the anti-Kir4.1_93-106_, we conclude that it is a Cter cleavage fragment of Kir4.1 missing its ∼100 first amino acids. Thus, the lowest molecular weight form of intact linear Kir4.1 monomer corresponds to the 37 kDa band. In conclusion, Kir4.1 expression is not only increased but also exhibits a shorter form in inflamed white matter.

### Recognition of the conformational aglycosylated e1 domain

As our mouse and HR anti-e1 sera poorly detected the linear forms of Kir4.1 in classical Western blots, we also used unreduced conditions (without DTT) using crude membrane extracts of a multiple sclerosis WM-enriched sample with Kir4.1 bands between 37 and 50 kDa. We hypothesized that keeping disulphide bonds including Cys108–Cys140^43^ would help maintain some e1 secondary structure, favouring its recognition by anti-conformational e1 antibodies. As shown in Fig. 7E, the Kir4.1 bands revealed by anti-Cter in reduced conditions were again barely detected with the mouse anti-e1 serum. The unreduced condition delayed Kir4.1 monomer migration as expected, with new signals detected between 130 and 120 kDa with the anti-Cter. Only, this latter band was detected with the mouse anti-e1 serum, suggesting recognition of the unreduced Kir4.1 monomer with its aglycosylated e1 domain.

**Figure 8:**
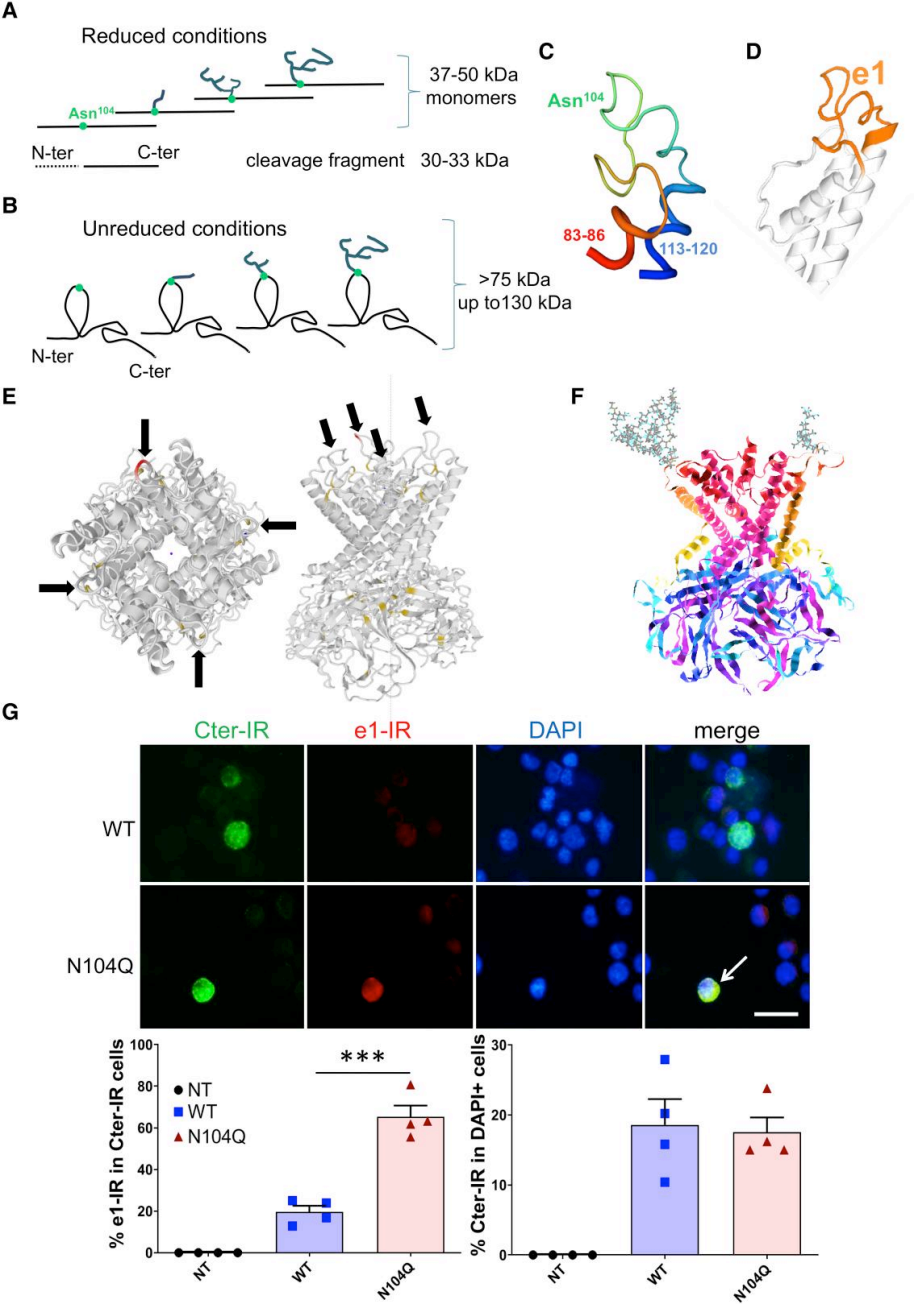
**Recognition of e1 sequence in relation to the 3D structure, N104 glycosylation and antibody accessibility. (A–F)** Schematic representation of CNS Kir4.1 forms with different glycosylation chains attached to the Asn104 and Kir4.1 3D model structure. **(A)** Under SDS-PAGE with reduced conditions, major Kir4.1 monomers were detected in the CNS between 37 and 50 kDa. A Cter fragment may be evidenced in diseased brain or after harsh extraction procedures or prolonged PNGase digestion. **(B)** Under non-reduced conditions, aglycosylated and e1-glycosylated Kir4.4 monomers migrate above 75 kDa, up to 130 kDa. **(C)** 3D modelization of e1 domain (amino acids 83–120) showing e1 loop (amino acids 87–114) maintained by hydrophobic amino acids 83–86 and 113–120 belonging to the transmembrane helices, and Asn104 exposed at the extracellular surface. Cartoon representations were obtained from the Swiss-Model 3D structure.^44^**(D)** e1 loop with the neighbouring transmembrane helices. (**E)** Localization of Asn104-containing e1 domain in Kir4.1 tetramer in two different plans, showing that the aglycosylated e1 sequence is well accessible to suspected anti-e1 autoantibodies. (**F)** Predicted Kir4.1 tetramer 3D structure with one complex glycan attached to one Asn104 (left subunit) and one simple glycan attached to another Asn104 (right subunit); obtained with GlyProt and Raster3D bioinformatic tools. **(G)** Upper panels: anti-Cter immunoreactivity (IR) versus anti-e1 IR obtained with anti-e1 serum and corresponding DAPI stains in wild-type Kir4.1 transfected cells (WT) versus N104Q Kir4.1 transfected cells. The arrow points to a cell highly labelled both with anti-Cter and anti-e1. Scale bar, 25 µm. Lower panels: quantification of the proportion of double-labelled cells (left) and proportion of transfected cells revealed by the anti-Cter in WT and N104Q transfected versus non-transfected (NT) cells. Two-tailed unpaired *t*-test: ***, *P* < 0.001 (*n* = 4 independent wells/group; *t* = 7.466, *df* = 6).

Taken together, Western blot data with immunocytochemical experiments suggest that the mouse or HR anti-e1 IgGs better recognize the aglycosylated conformational e1 domain (Fig. 8A and B). In order to better visualize epitope accessibility, we used free-available bioinformatics tools. As shown in Fig. 8C–E, the peptide sequence around Asn104 appears well accessible to putative antibodies when Asn is not glycosylated. Using the GlycoEP prediction tool,^45^ we checked that the sequence flanking Asn104 indeed fulfilled the characteristics of a prototypic *N*-glycosylation site (score: + 0.81 for Kir4.1). In contrast, Asn104 is not in a favorable *N*-glycosylation site for Kir5.1 (score: −0.78). The GlyProt tool^46^ was used to visualize *in silico* a simple or complex *N*-glycans attached to Asn104 on the 3D protein structure of Kir4.1 tetramer, showing that glycosylation of the e1 epitope can hinder accessibility to conformational e1 autoantibodies (Fig. 8F).

Finally, to support the importance of the glycosylation state in the e1 loop for antibody recognition, we used CHO cells that were transfected with a wild-type human Kir4.1 construct versus its mutant with defective *N*-glycosylation at amino acid 104 (N104Q). Indeed, while Cter antibody detected equally WT and N104Q transfected cells, anti-e1 serum recognized 3-fold better N104Q Kir4.1 transfected cells than the WT version (Fig. 8G).

## Discussion

Our work provides evidence that the development of anti-Kir4.1(e1) autoreactivity promotes encephalomyelitis in mice with neurological symptoms that are associated with CNS demyelinating lesions. All the immunized mice developed high levels of serum anti-e1 IgGs, suggesting that tolerance to this sequence can be broken despite peripheral Kir4.1 expression. This is the first report showing that the immunization with the e1 sequence promotes *in vivo* CNS autoimmune damage, supporting Kir4.1 as a valid antigen candidate that could favour CNS autoimmunity. We indeed found that a subset of multiple sclerosis patients exhibits high serum levels of anti-e1 IgG. Moreover, our data point that the peculiar autoreactivity against the conformational aglycosylated e1 domain explain why previous cell-based assays or classical Western blot were not suitable to accurately detect this specific autoreactivity.^5,47^

### Anti-e1 autoreactivity promotes central nervous system immune cell infiltration and demyelinating encephalomyelitis

A specific feature of e1-immunized mice is the development of atypical EAE associated with typical EAE. Accordingly, neuroinflammation and patches of demyelination occurred widely in the CNS of these mice, from spinal cord to cerebellum and forebrain with immune cell infiltration mainly concentrated in the WM tracts. Another specific observation is the unexpected presence of B cells in WM perivascular regions and periventricular or spinal cord leptomeninges. Moreover, in the cerebellum of mice severely affected by atypical EAE, demyelinated foci with high inflammatory cuffs were associated with adjacent alterations in the Purkinje cell layer and Bergman glia integrity. Symptoms of cerebellar dysfunction (ataxia, dysarthria or tremor) are commonly observed in chronic progressive multiple sclerosis, and in few cases Purkinje cell loss adjacent to focal demyelinated folia has been indeed reported.^48,49^ Whether such focal leukocortical lesions associated with adjacent grey matter lesions can be a feature of deleterious Kir4.1 autoreactivity in humans—as observed in e1-immunized mice—will require further investigation. Besides cerebellum, most grey matter and WM astrocytes were preserved in e1-immunized mice since there was no cavitation such as observed in prototypic astrocytopathies (e.g. Alexander disease). Moreover, high glycosylated Kir4.1 may protect most cell populations from autoantibody recognition. It is striking that the aglycosylated e1-immunoreactivity was not detected in grey matter Kir4.1-positive astrocytes. Accordingly, in our HR patients, no neurological signs associated with grey matter astrocytic Kir4.1-linked dysfunctions were reported, such as those reported in autism-associated disorders^50^ or Huntington’s disease model.^51^ Similarly, we did not see anti-conformational e1-immunoreactivity on Cter-immunoreactive distal tubules in human kidney biopsies. KCNJ10 mutations are often associated with SeSAME syndrome including salt retention deficits in tubules that express Kir4.1.^24,25^ However, the HR patients had no reported signs of kidney dysfunction as in multiple sclerosis in general. This suggests that the e1-autoimmunity develops in the inflamed CNS but not in periphery. Indeed, Kir4.1 expression is weak in normal white matter but increases in EAE and multiple sclerosis. The aglycosylated e1-immunoreactivity follows this pattern supporting the notion that preferential targets of e1 autoreactivity reside in oligodendroglia and some astrocytes, especially during neuroinflammation. Interestingly, KCNJ10 mutations are not necessarily associated with renal tubulopathy^52^ and have been recently associated with WM oedema, supporting an important role of Kir4.1 for CNS glia,^53^ restricted slowly progressive ataxia and cerebellar atrophy^53,54^ or progressive spasticity,^55^ thus giving importance of the phenotypic heterogeneity and localized role of Kir4.1 dysfunction.

### Importance of detecting anti-conformational e1 epitope autoreactivity in multiple sclerosis

Since the initial report indicating that half of multiple sclerosis patients exhibit anti-Kir4.1(e1) IgG, low reliability of anti-e1 IgG reactivity in multiple sclerosis has been claimed by others. Transfected cells are often used to assess IgG reactivity towards MOG or other candidate antigens in their most natural conformational state.^14,56^ However, these cells can have high glycosylation activity and thus may not be suitable to reproduce aglycosylated or low glycosylated candidate CNS autoantigens. Our data imply that standard cell lines may not be adequate to screen anti-e1 antibodies. Indeed, the high level or complexity of Kir4.1 glycosylation by cell lines such as the U-251 line does not correspond with the endogenous protein expressed by glial cells *in situ*, especially during neuroinflammation, where the e1-glycosylation status is reduced. Another issue is that glycosylation generally results in protein stabilization.^57^ The reduced glycosylation state of Kir4.1 during inflammation may favour protein degradation and help antigen processing by immune cells. We indeed observed more Kir4.1 cleavage product when the CNS extracts were subjected to prolonged deglycosylation *in vitro*. The presence of antibody against myelin antigens and e1 from Kir4.1, associated with the reduced glycosylation state during neuroinflammation, may synergistically favour disease progression. Moreover, we noticed in immunized mice that B cells were present around blood vessels or in leptomeninges but had not infiltrated deeply the parenchyma, in line with the few cells of the B lineage reported in the neuroparenchyma in multiple sclerosis. Previous work using MOG-derived models has uncovered various modes of actions to explain how B cells and autoantibodies mediate autoimmune demyelination, including the deleterious role of autoantibodies,^58,59^ the priming of T cells by peripheral antigen-specific B cells^60^ and the reactivation of T cells in the CNS by B cell-derived cytokines^61^ or via secreted autoantibodies which concentrate and increase antigen presenting capacity.^62^ These different scenarios may apply to several antigens including Kir4.1.

In conclusion, the glycosylated status of Kir4.1 e1 sequence likely prevents deleterious autoreactivity in the periphery and normal CNS; however, decreased glycosylation during autoimmune neuroinflammation and high levels of anti-conformational e1 antibodies may favour a more neuropathogenic process. Our findings imply that lower e1 glycosylation expose a conformational neoepitope to the immune system. In line with this, it has been shown that decreasing general maturation of *N*-glycans in mice can lead to spontaneous autoimmunity^63^ with glycosylated myelin proteins normally contributing to the maintenance of immune tolerance.^64^ Similarly, *N*-glycosylation can favour immune evasion for non-self antigens.^30^ Strikingly, dysregulation of *N*-glycosylation due to combined genetic and environmental factors may increase the risk of multiple sclerosis.^65^ The link between dysregulated protein glycosylation in autoimmune diseases needs to be further addressed to open new avenues of specific biomarkers. Moreover, improving the knowledge of the various antigen reactivities is important since possible induction of specific Tregs using antigenic peptide-coated nanoparticules^66^ or of antigen-specific B regulatory cells^67^ may be used in the future to control the autoimmunity process.^68^

## Supplementary Material

fcad044_Supplementary_Data

## Data Availability

*All data are available from the corresponding author, upon request by a qualified researcher.*

## Abbreviations

BBB =: blood brain barrier
CFA =: complete Freund adjuvant
CIS =: clinically isolated syndrome
DAPI =: 4′,6-diamidino-2-phenylindole
DDT =: 1,4-dithiothreitol
EAE =: experimental autoimmune encephalomyelitis
HRP =: horseradish peroxidase
HR =: high responders
HV =: healthy volunteers
IFA =: incomplete Freund adjuvant
IgG =: immunoglobulin G
IR =: immunoreactive
Kir4.1 =: inwardly rectifying potassium (Kir) channel 4.1
MOG =: myelin oligodendrocyte glycoprotein
MW =: molecular weight
NAWM =: normal appearing white matter
PTX =: pertussis toxin; WM = white matter
