# Supplementary material for: Aglycosylated extracellular loop of inwardly rectifying potassium channel 4.1 (KCNJ10) provides a target for autoimmune neuroinflammation
Source: Brain Commun. 2023 Feb 22;5(2):fcad044. doi: 10.1093/braincomms/fcad044 (PMC9994600; doi:10.1093/braincomms/fcad044)
Supplement: fcad044_Supplementary_Data [file fcad044_supplementary_data.zip › Supplementary_Material2.pdf]

# Additional separate channel images

## Figure 2 D to F

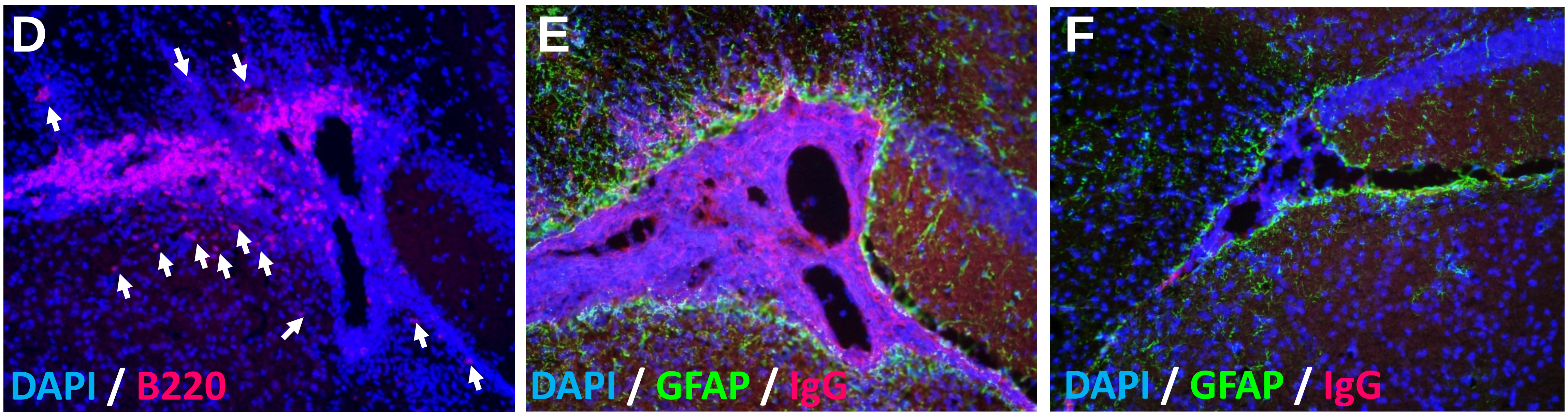


Corresponding separate channel images

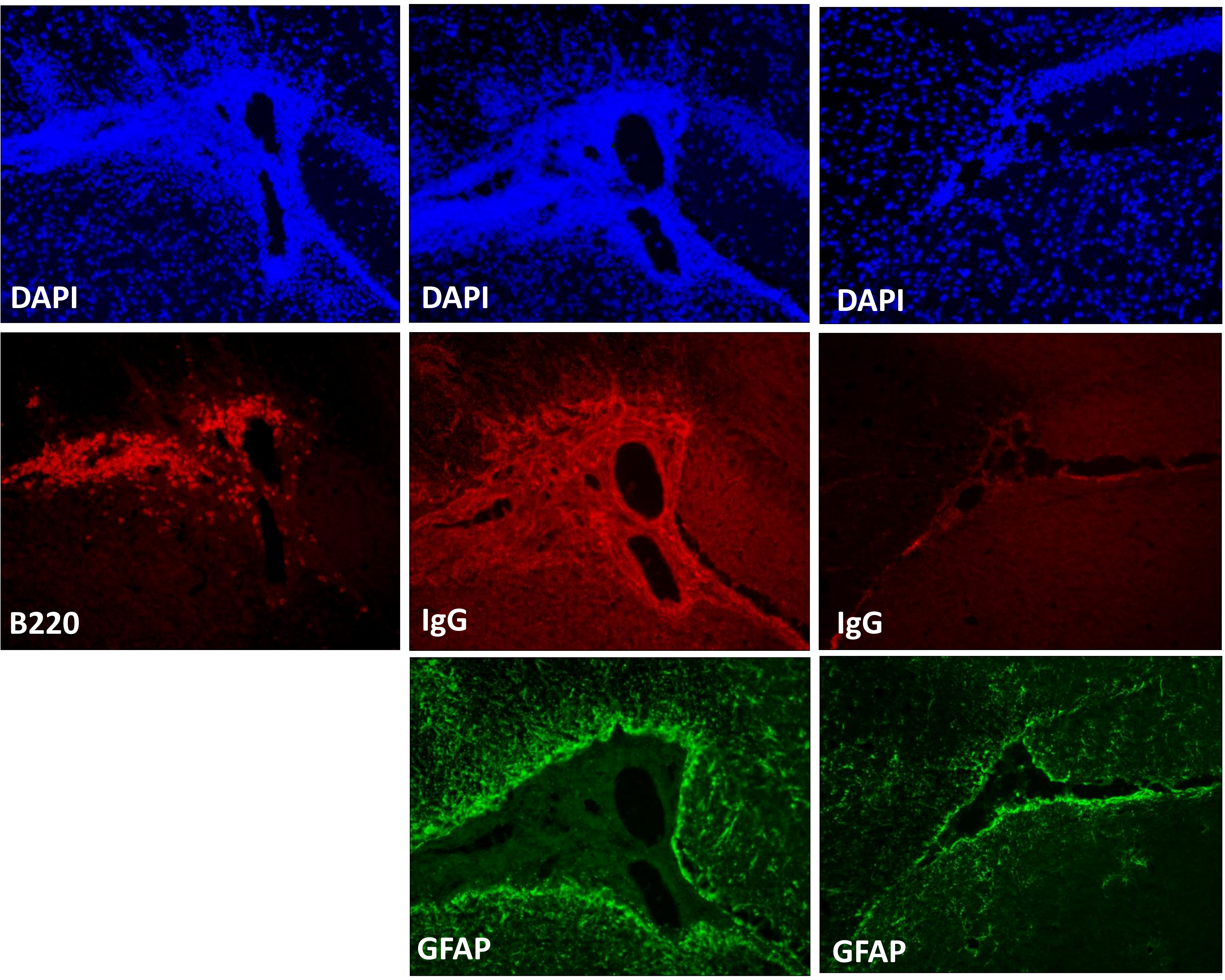

**Figure 2J**

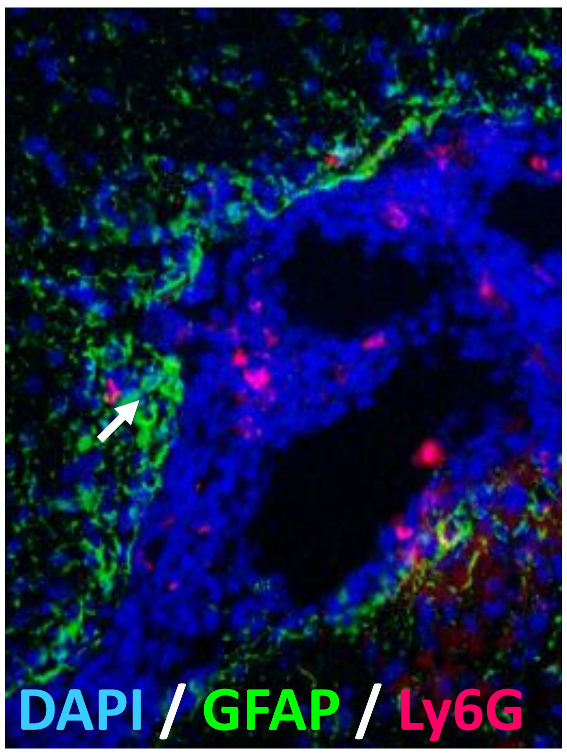


Corresponding separate channel images

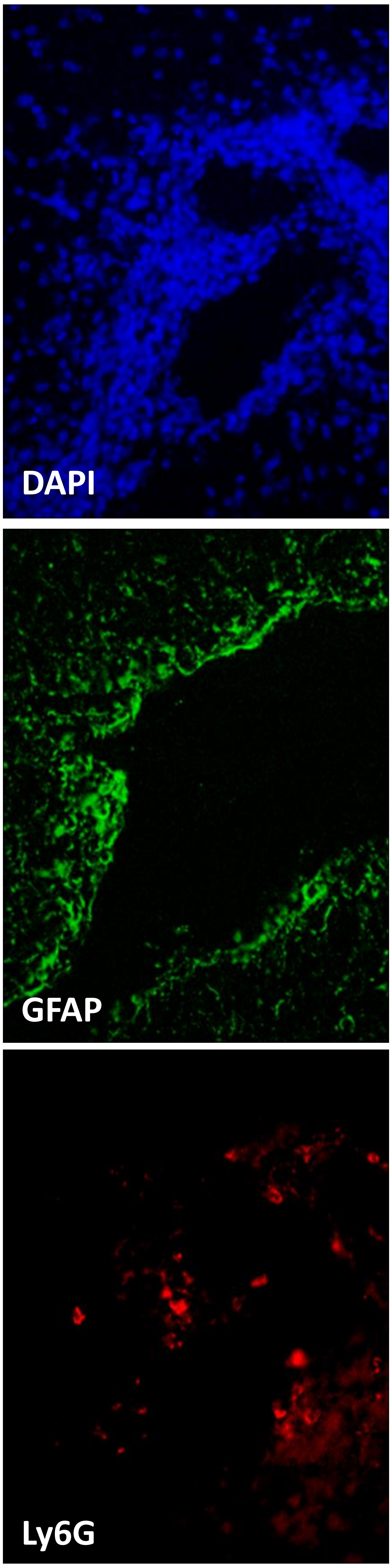

# Figure 2 Q-R

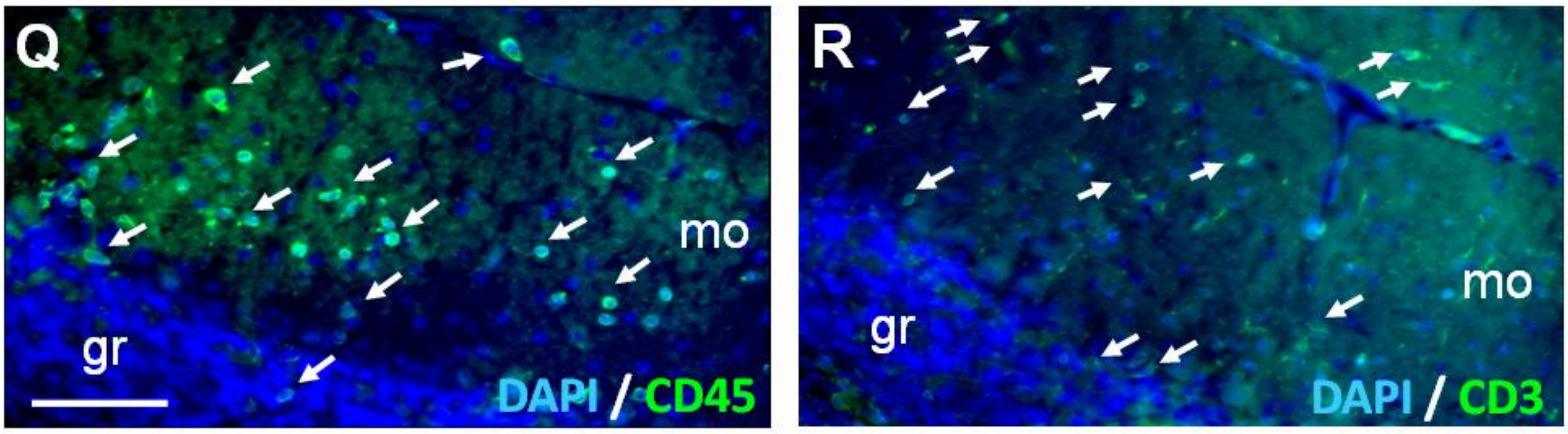


Corresponding separate channel images

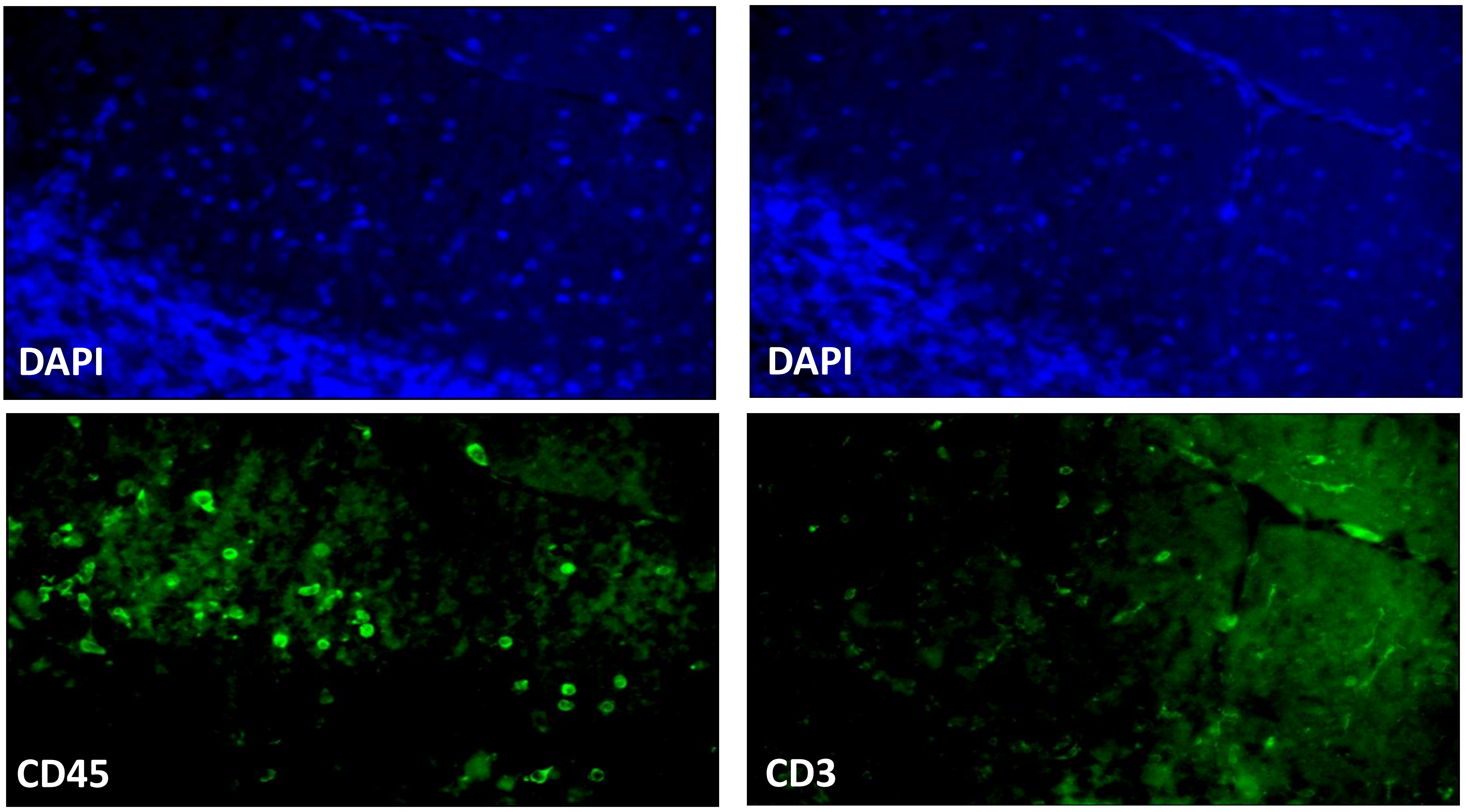

**Figure 3A**

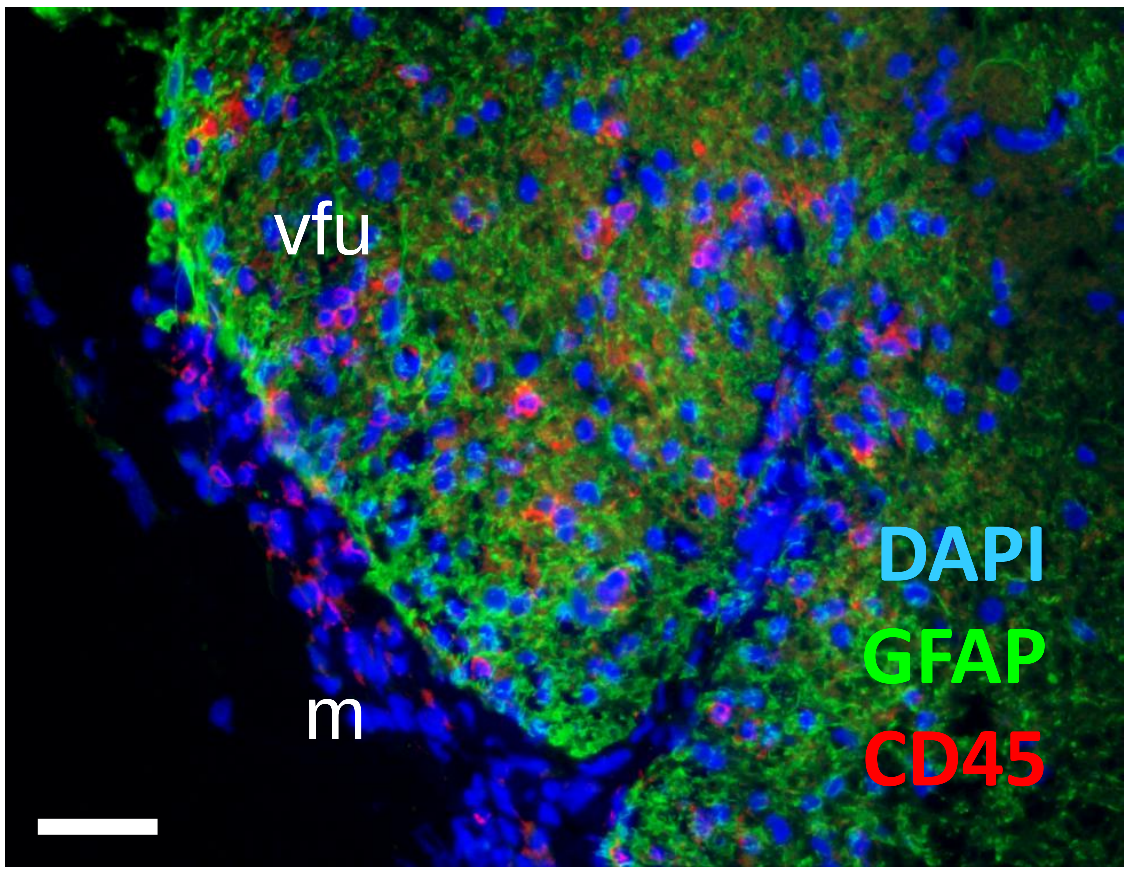


Corresponding separate channel images

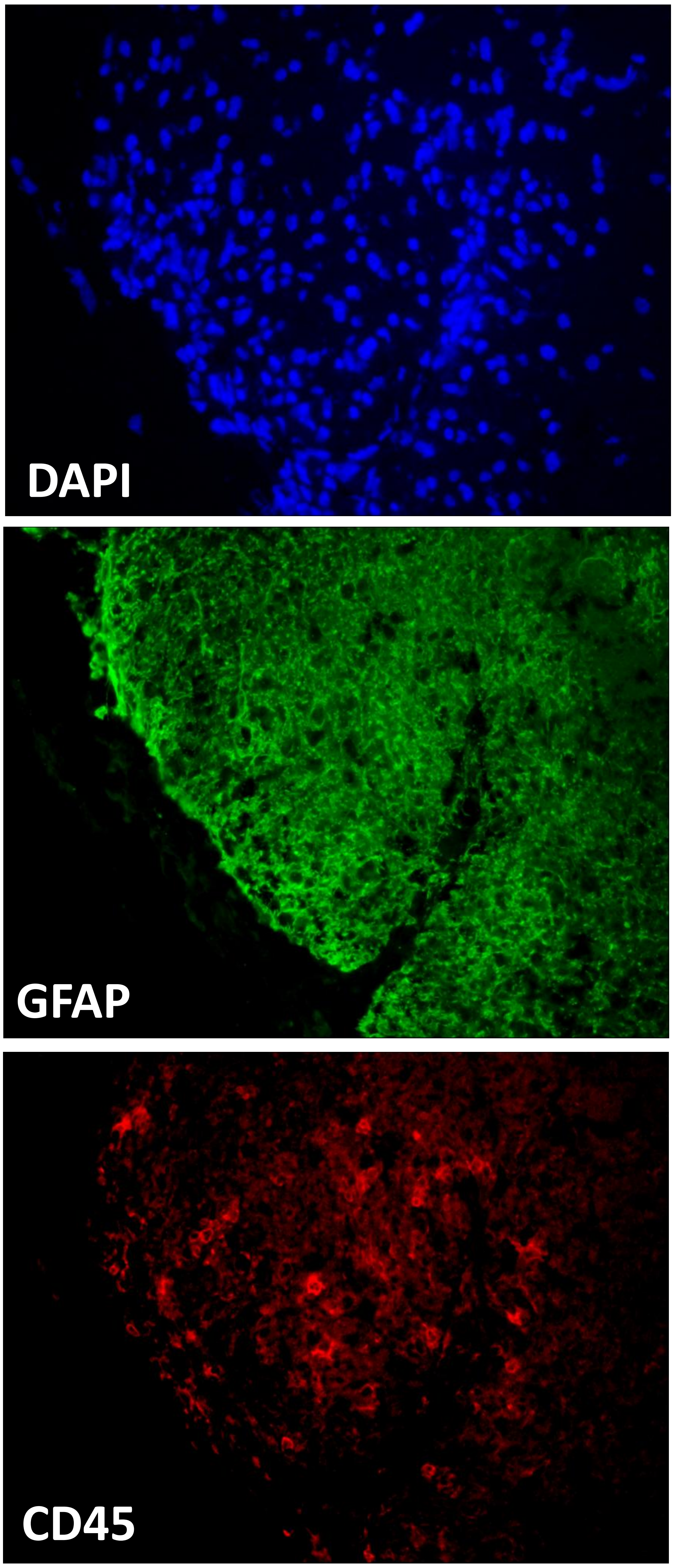

**Figure 3B**

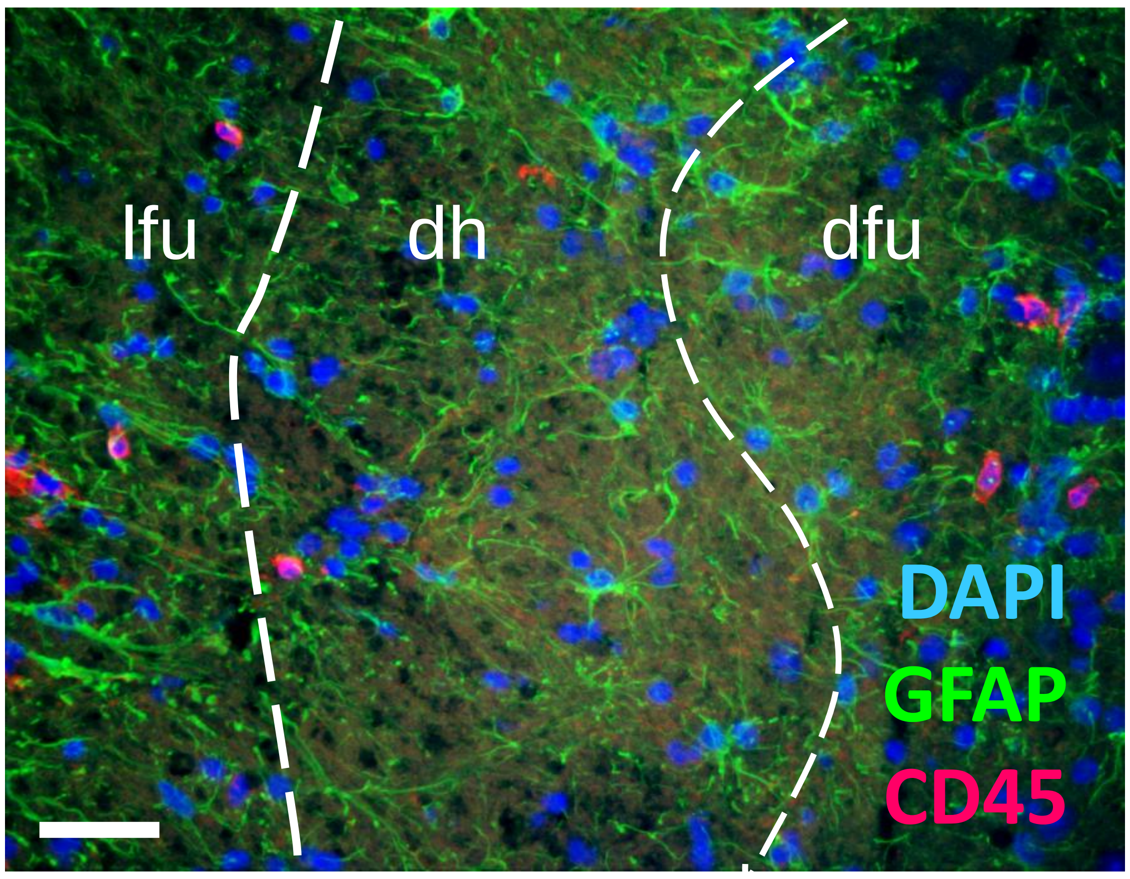


Corresponding separate channel images

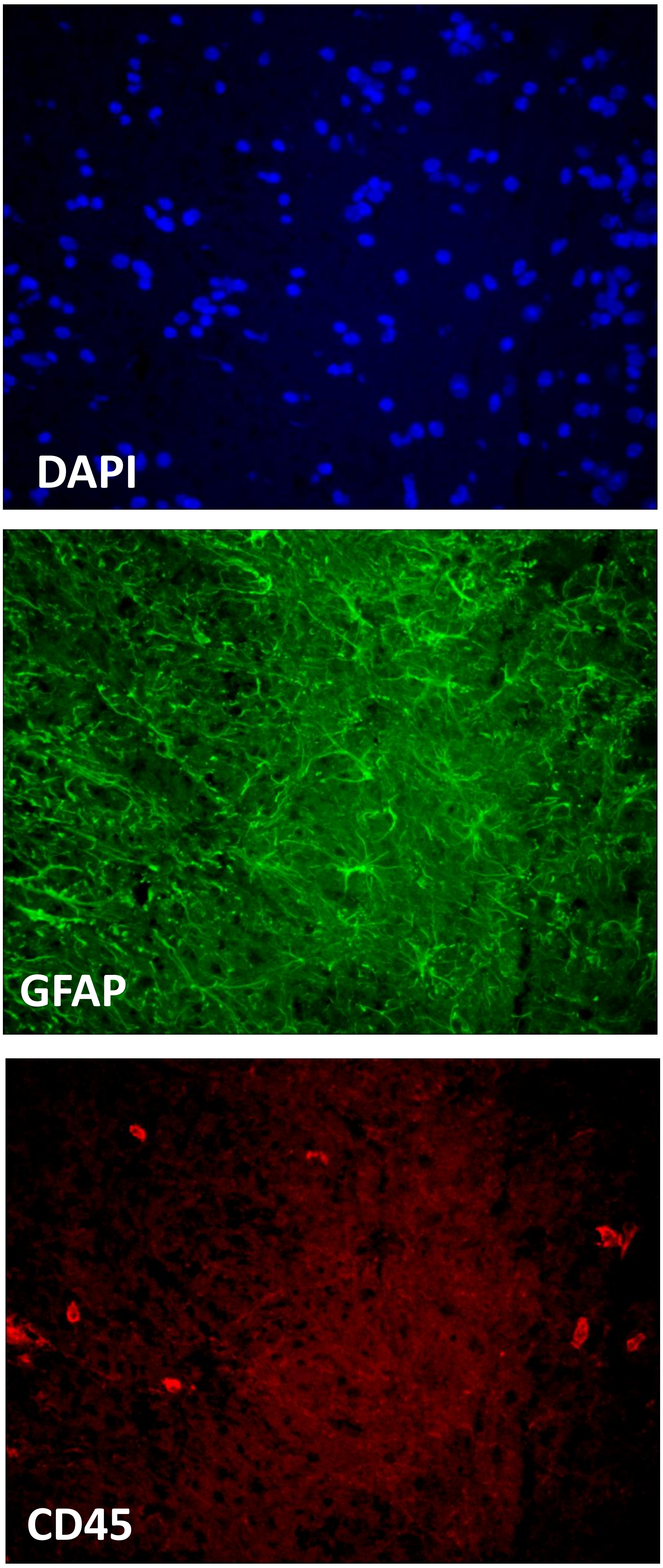

## Figure3C

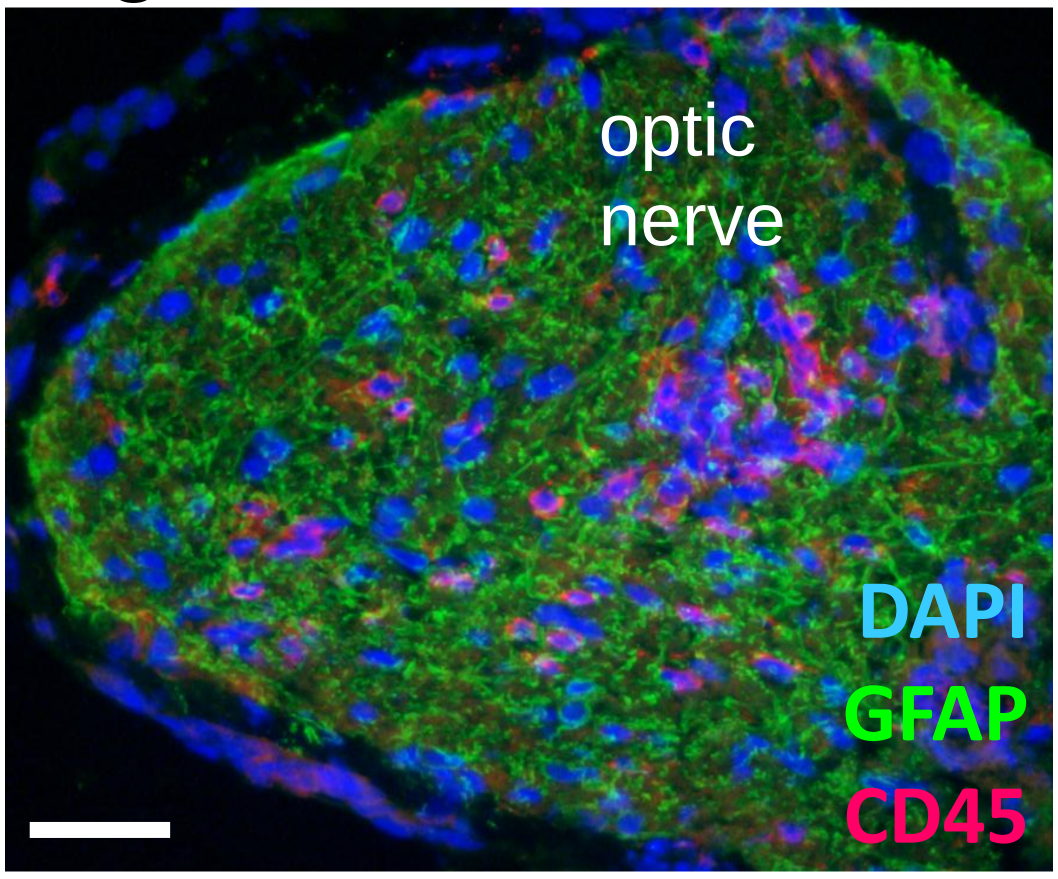


Corresponding separate channel images

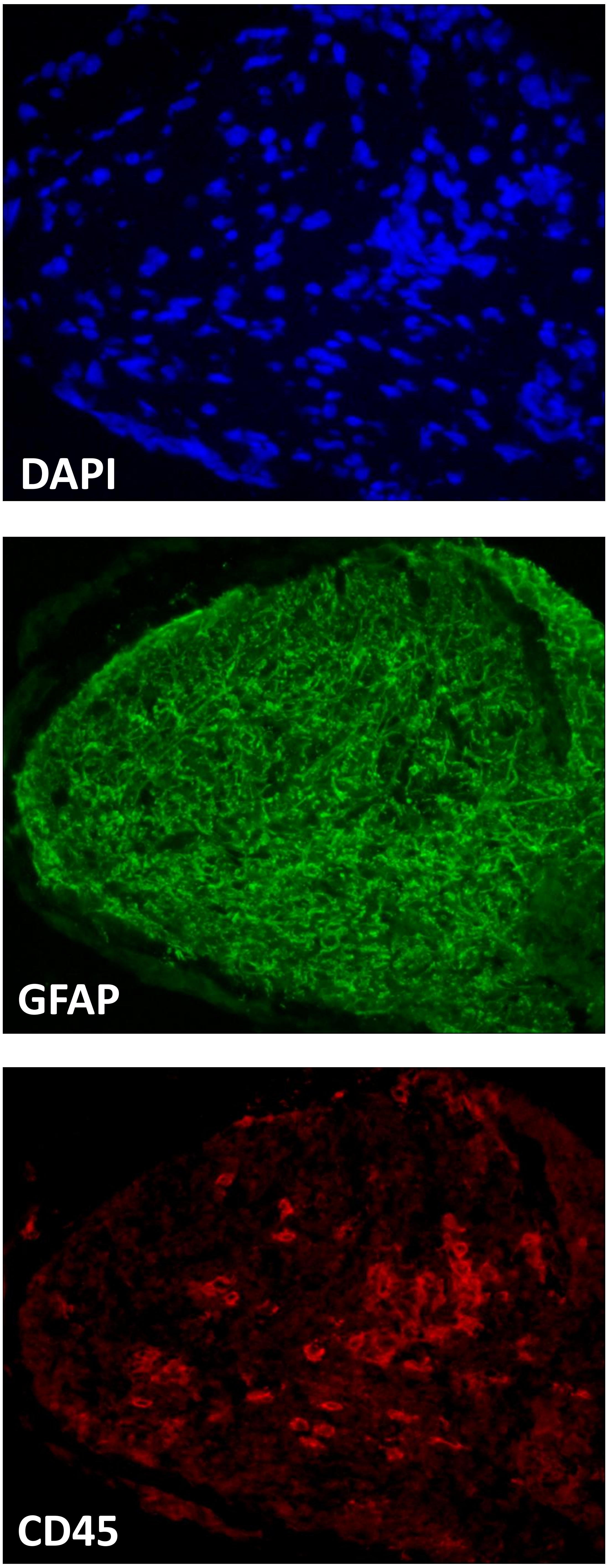

**Figure 3F**

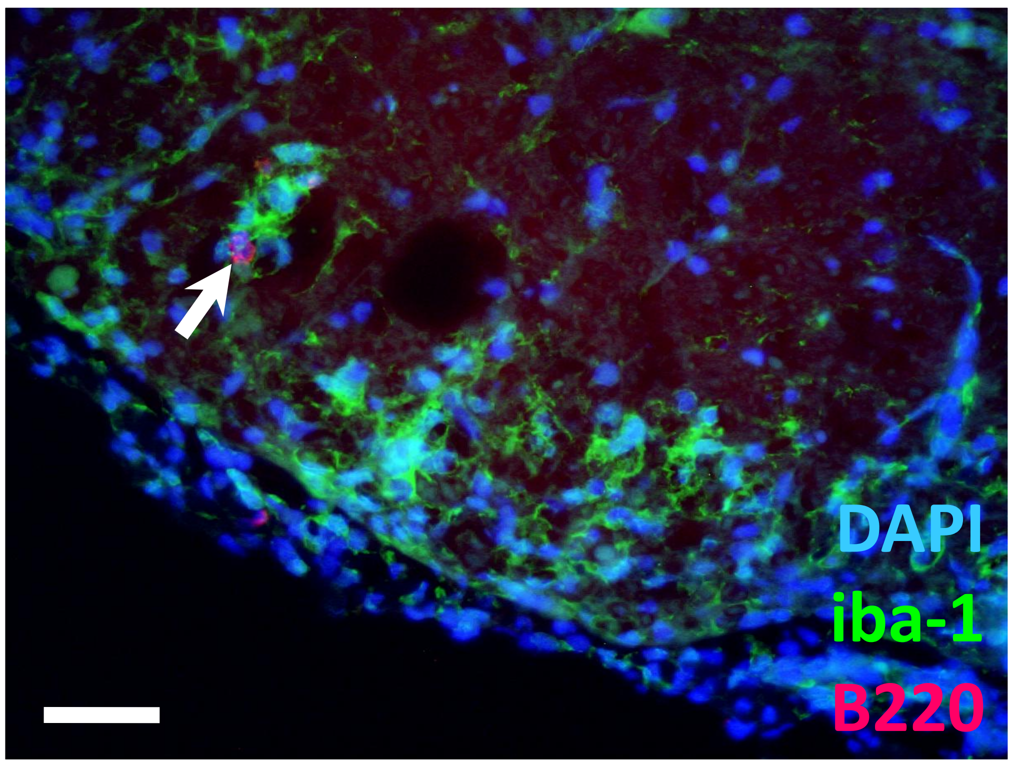


Corresponding separate channel images

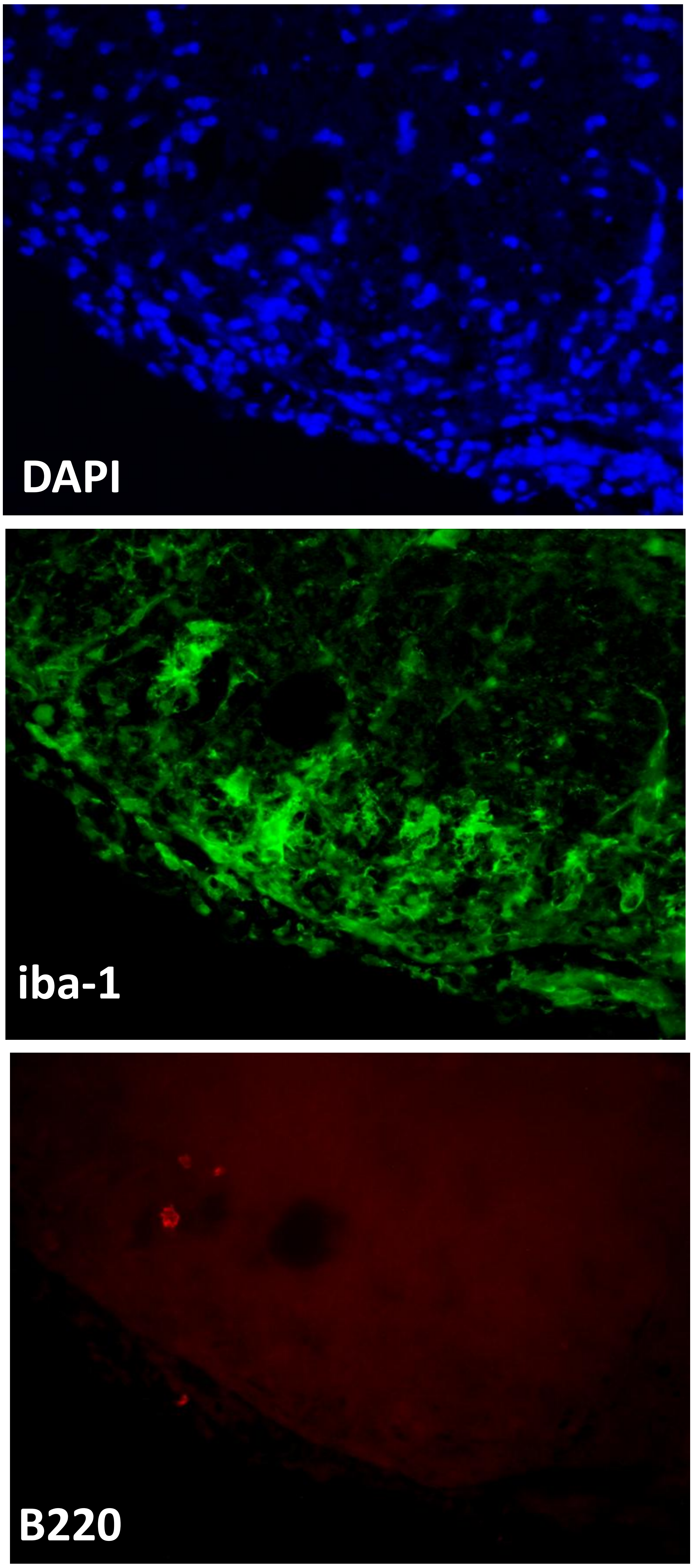

**Figure 3G**

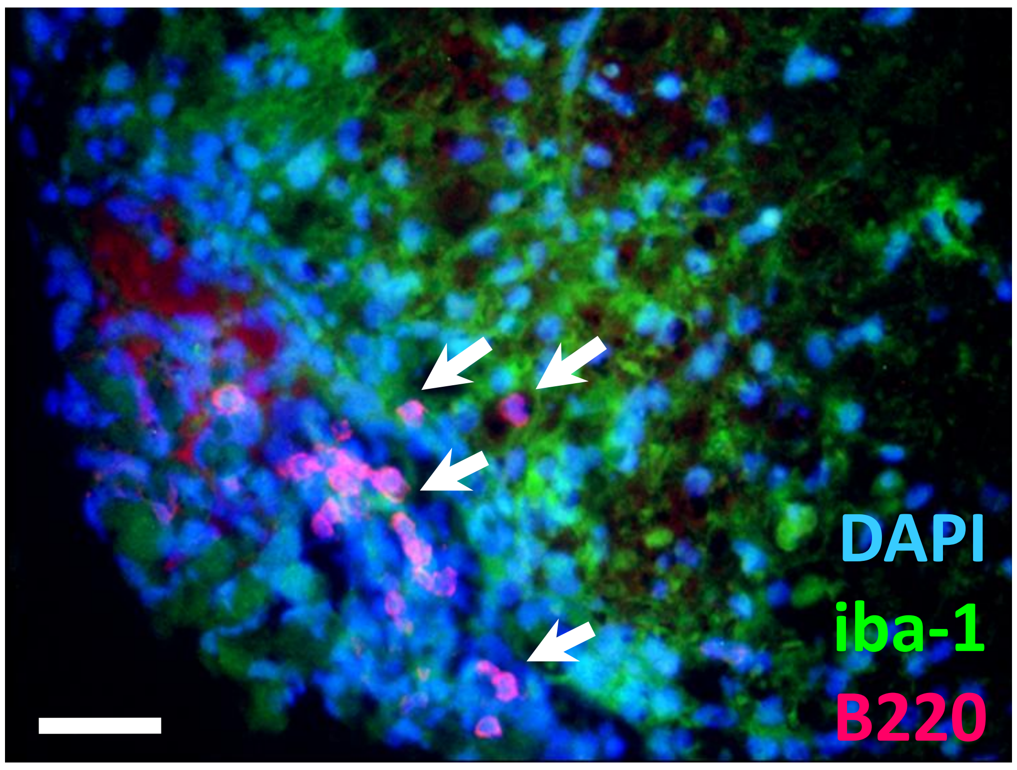


Corresponding separate channel images

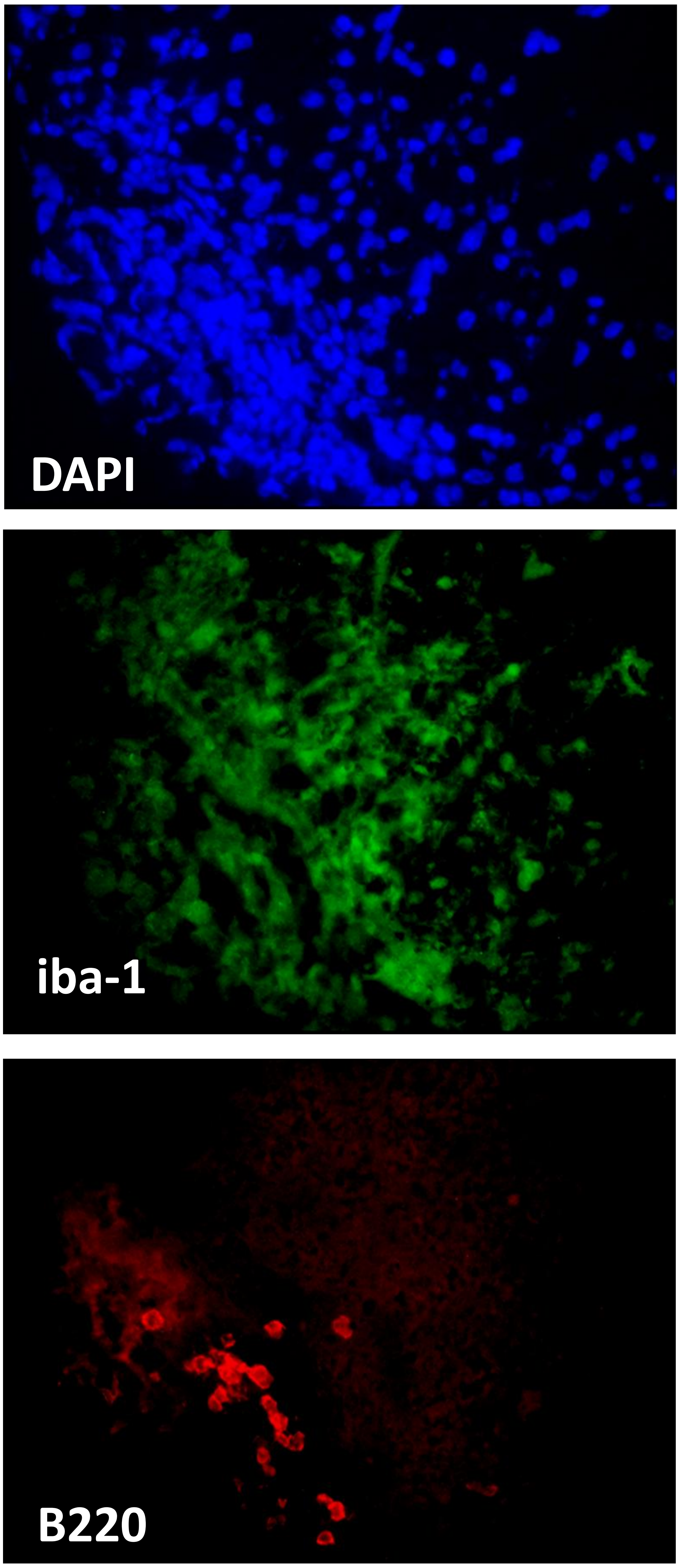

# Figure 3 I-K

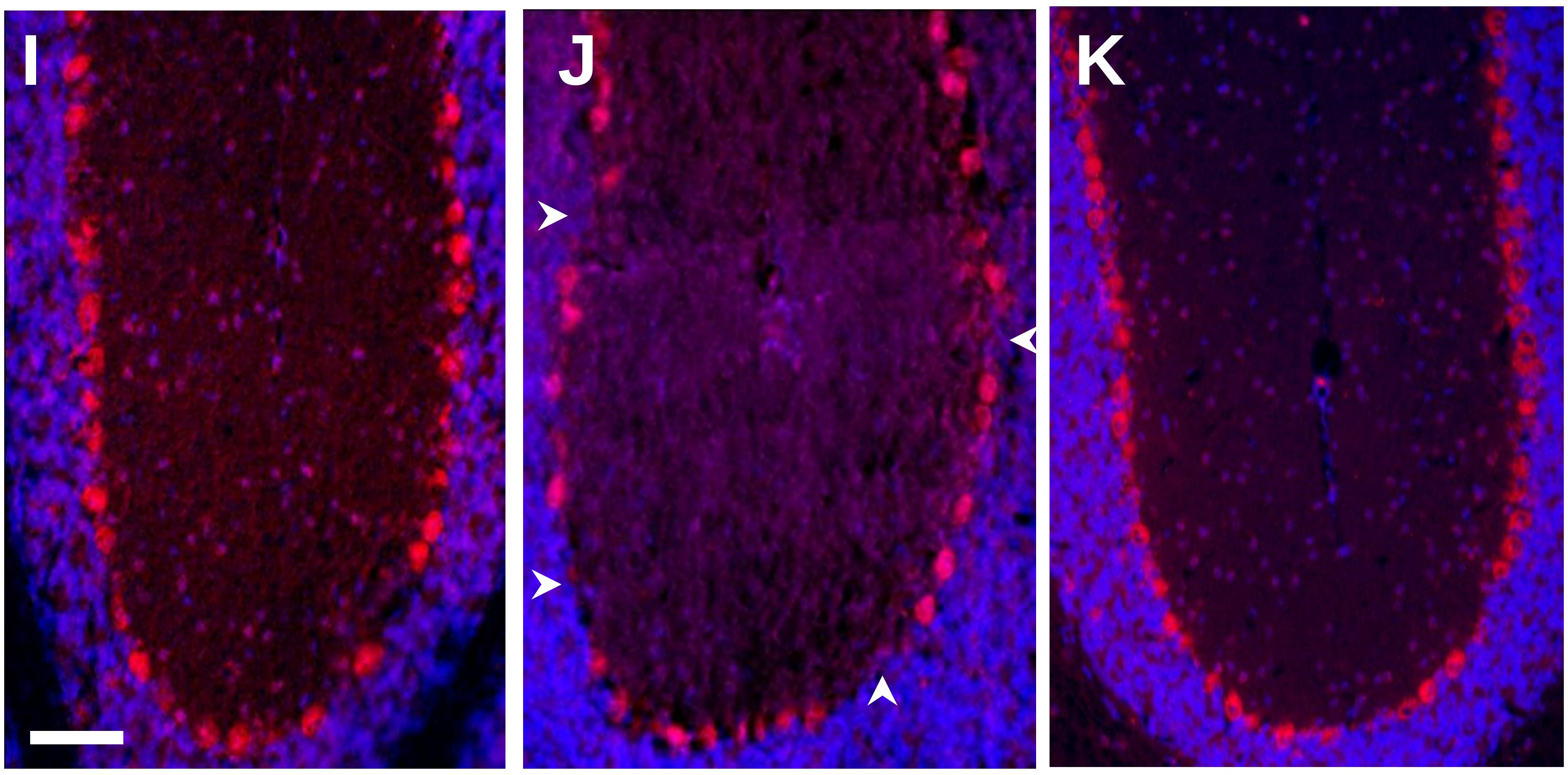


DAPI / Neurotrace in cerebellum

Corresponding separate channel images

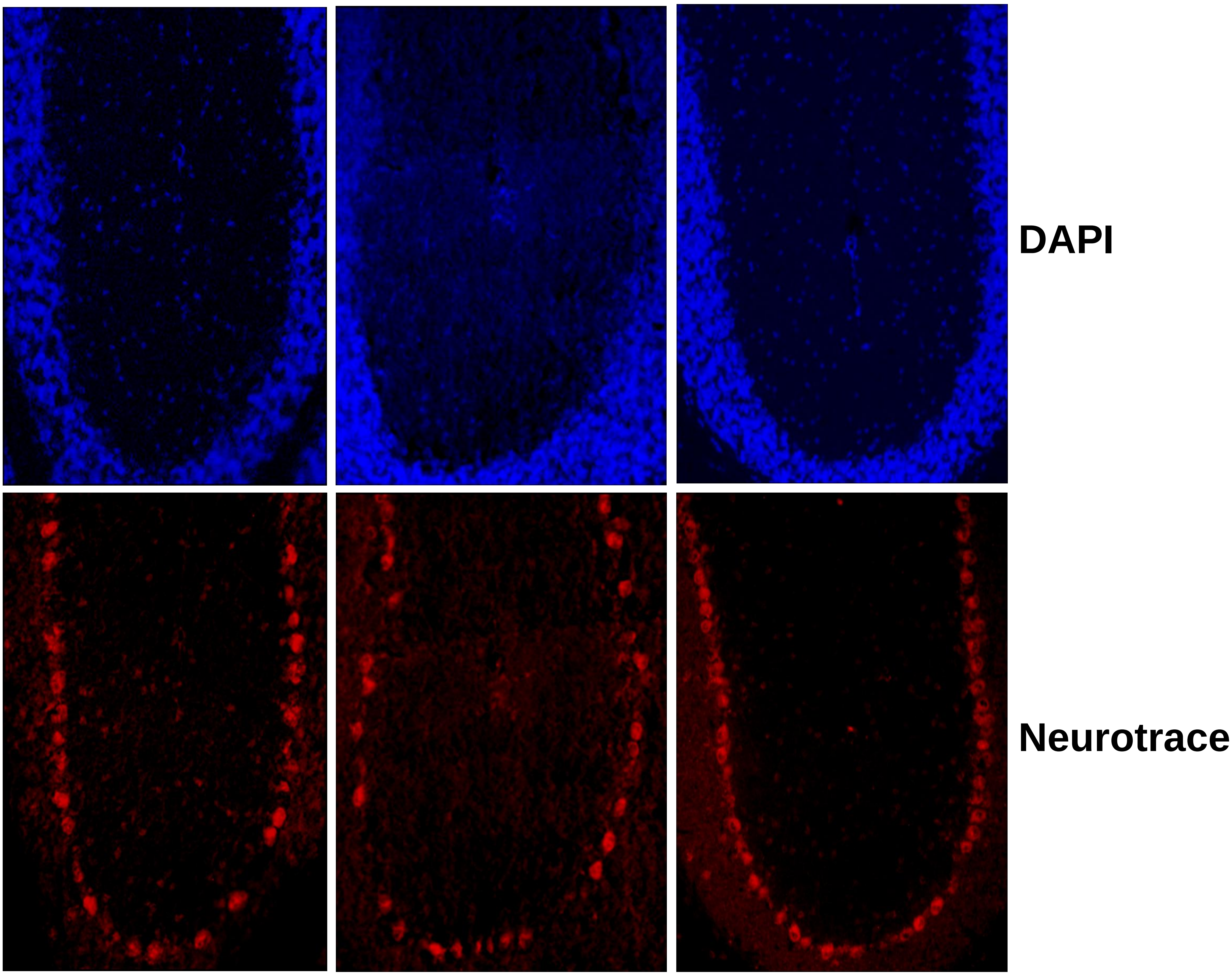

# Figure 3 L-N

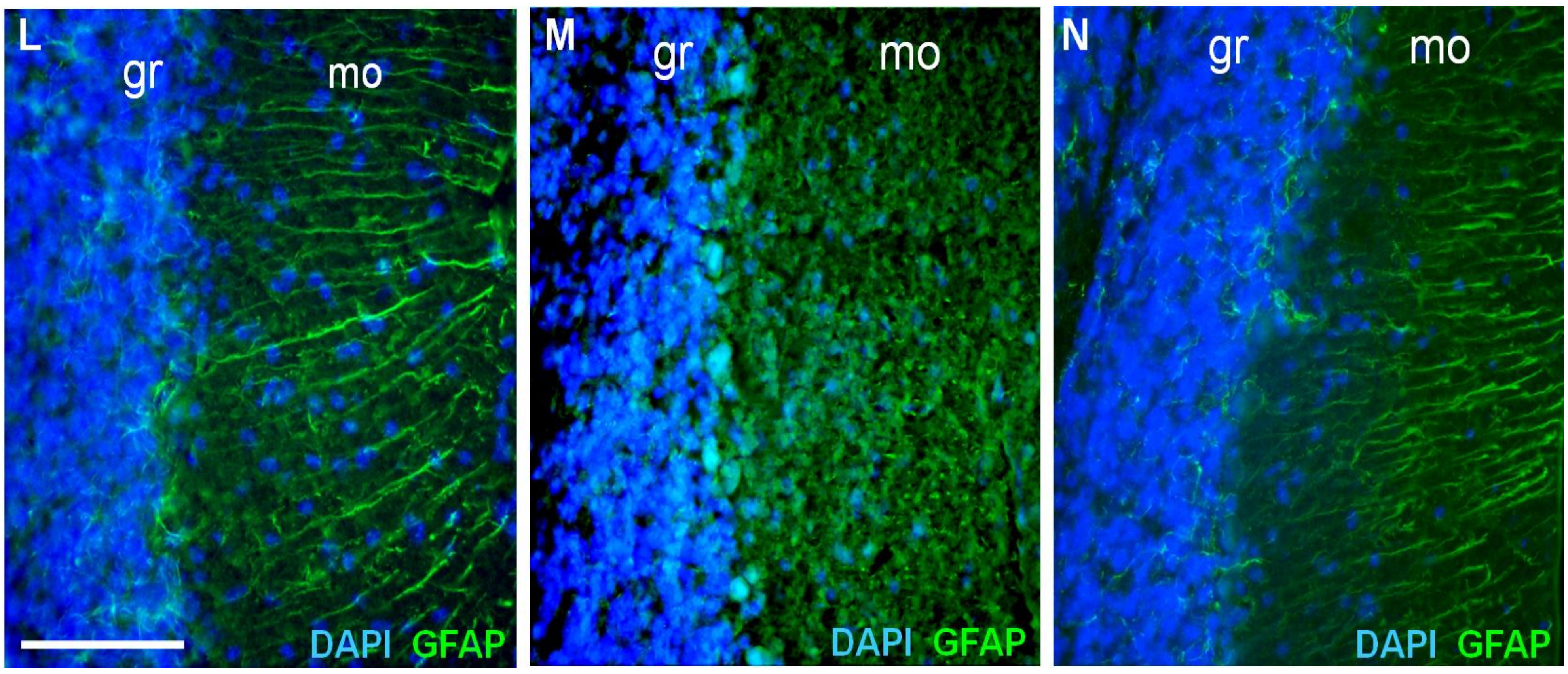


Corresponding separate channel images

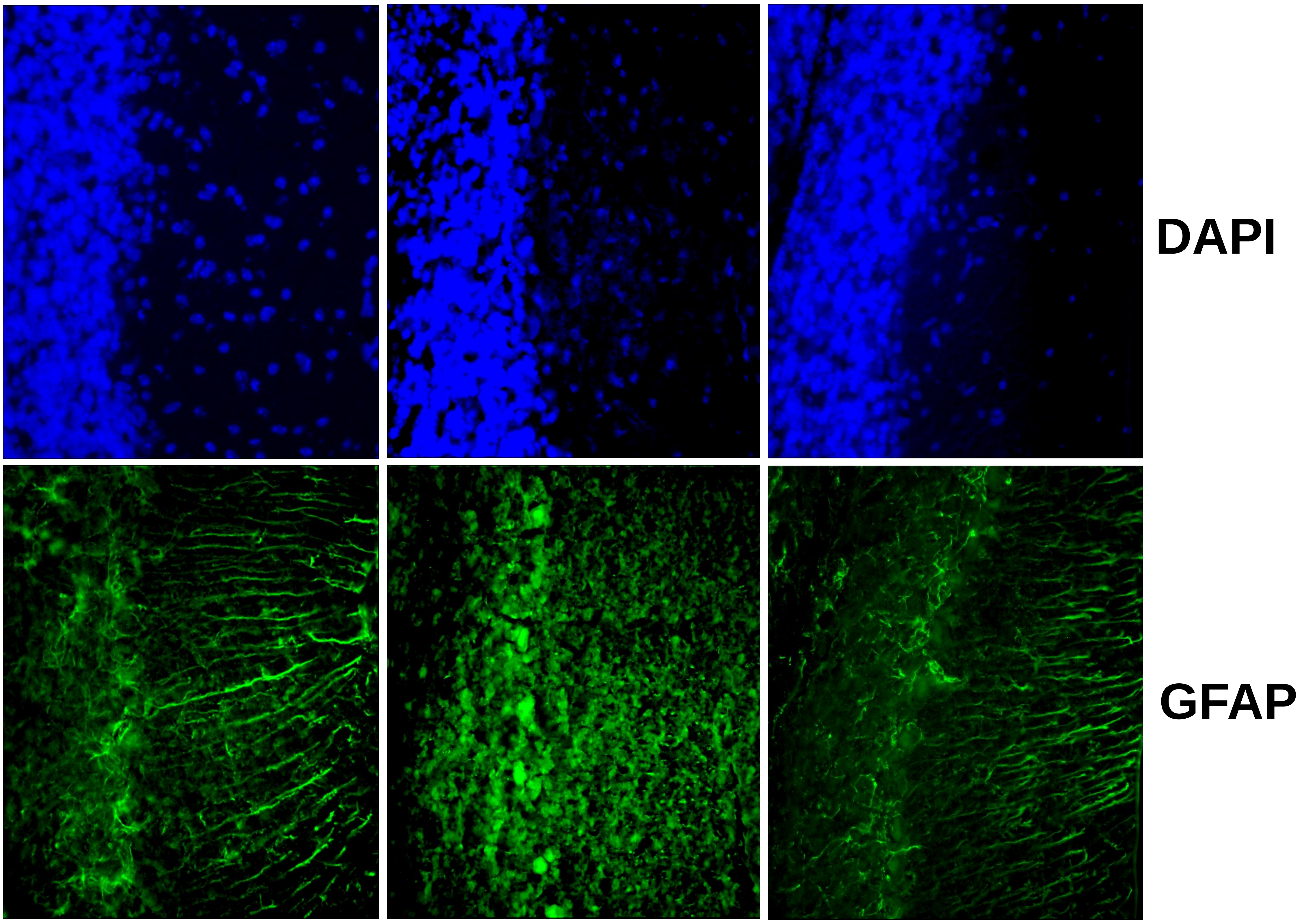

# Uncropped Western blot images and cropping steps

## unedited Western Blots for Figure 7 A

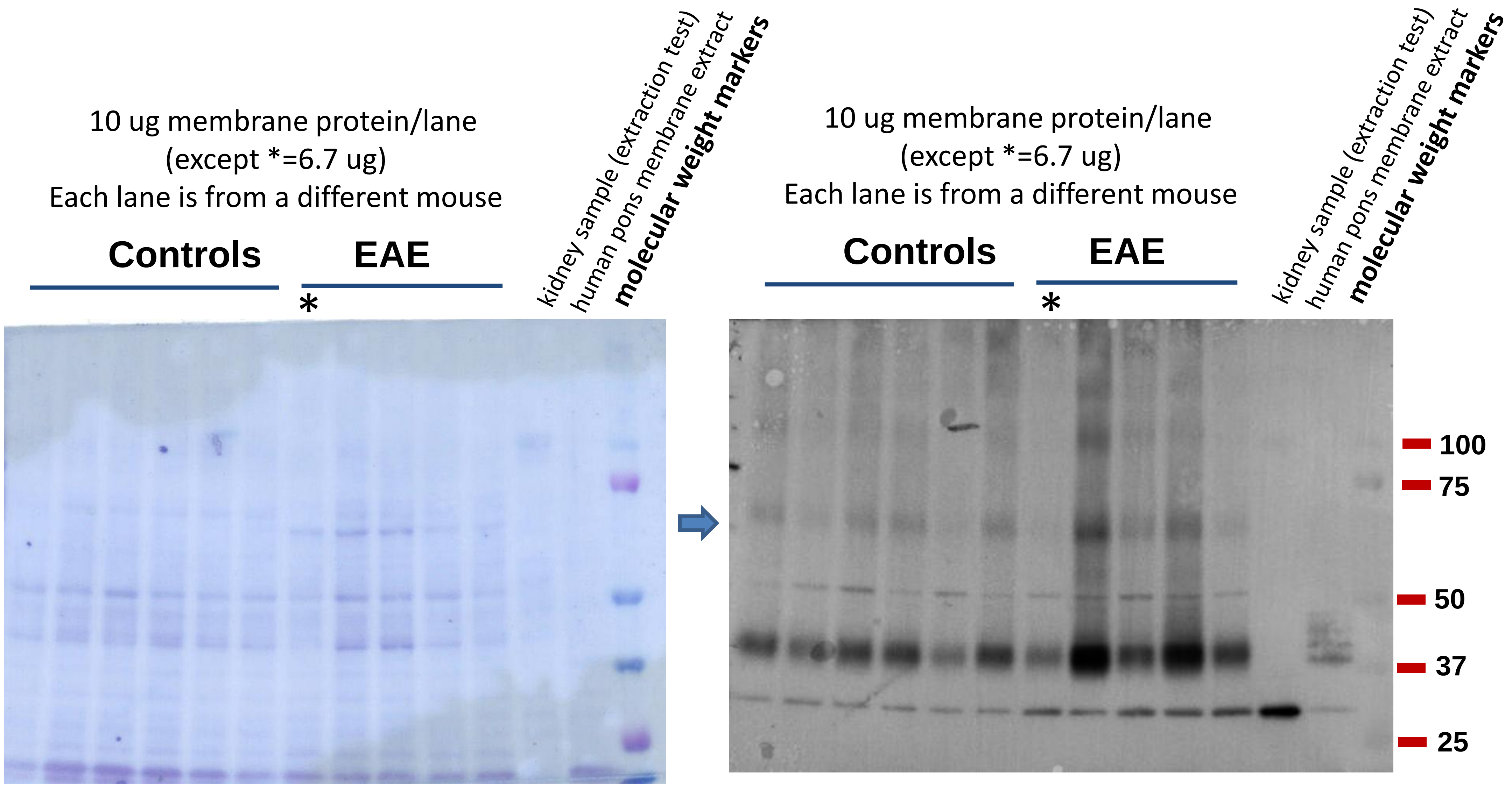


DB71 protein stain on PVDF

Brightfield + WB anti-Cter

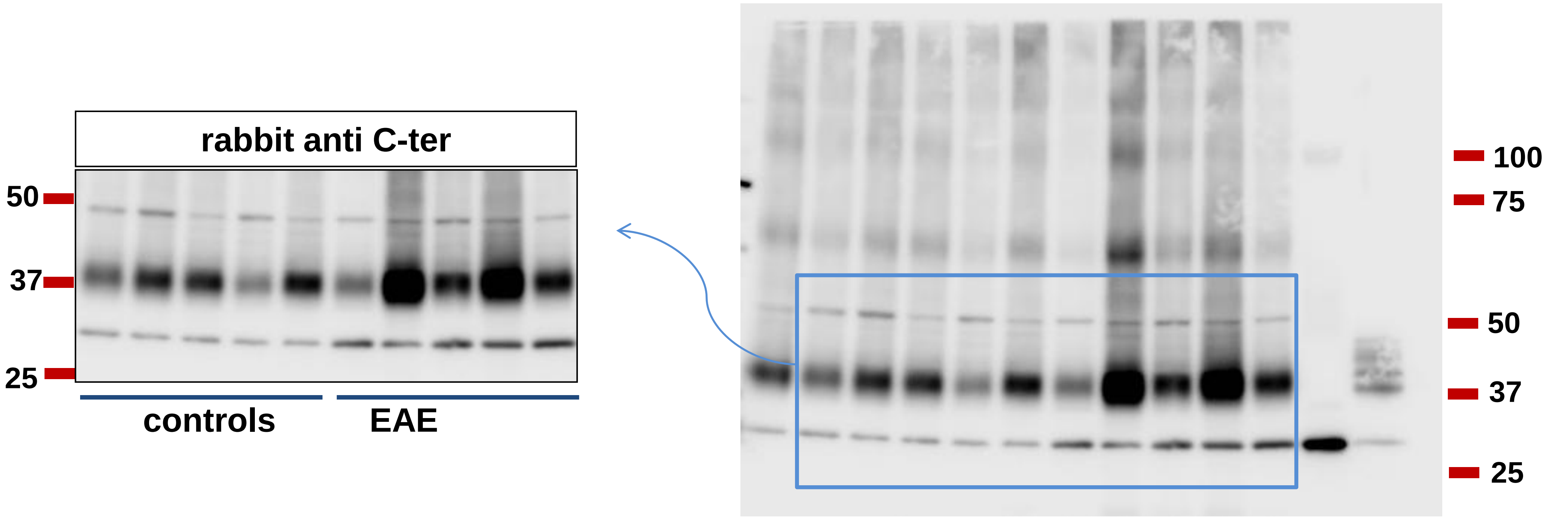


WB anti-Cter (overnight antibody incubation)

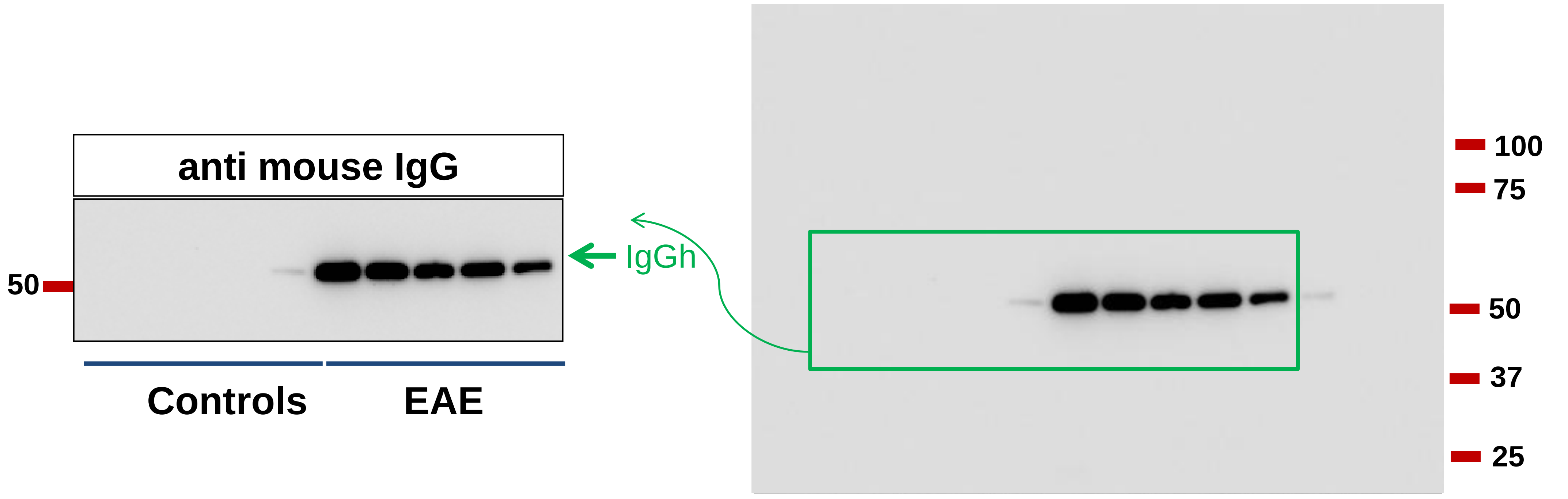


WB anti-mouse IgG

unedited Western Blot
for Figure 7 B

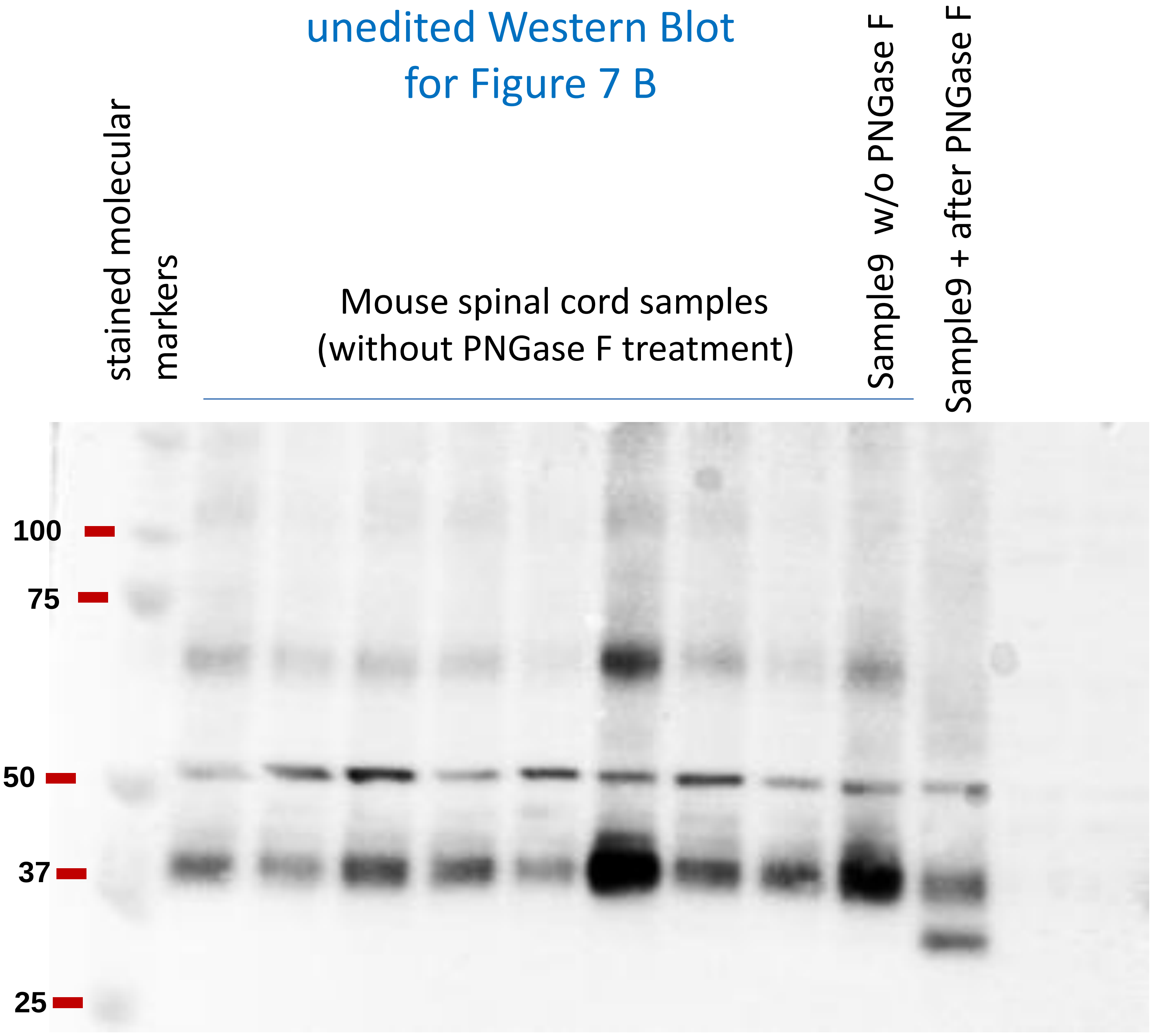


Brightfield + WB anti-Cter

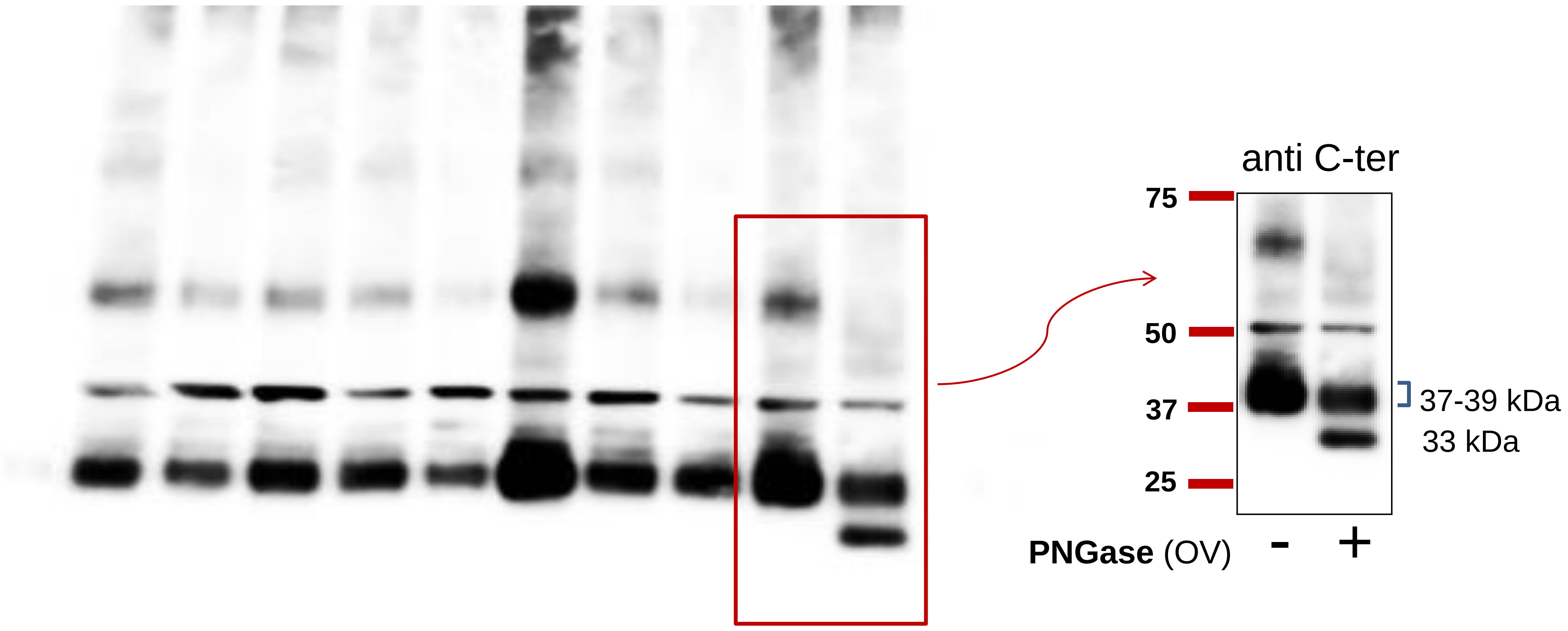


WB anti-Cter (1h primary antibody incubation)

# unedited Western Blots
# for Figure 7C

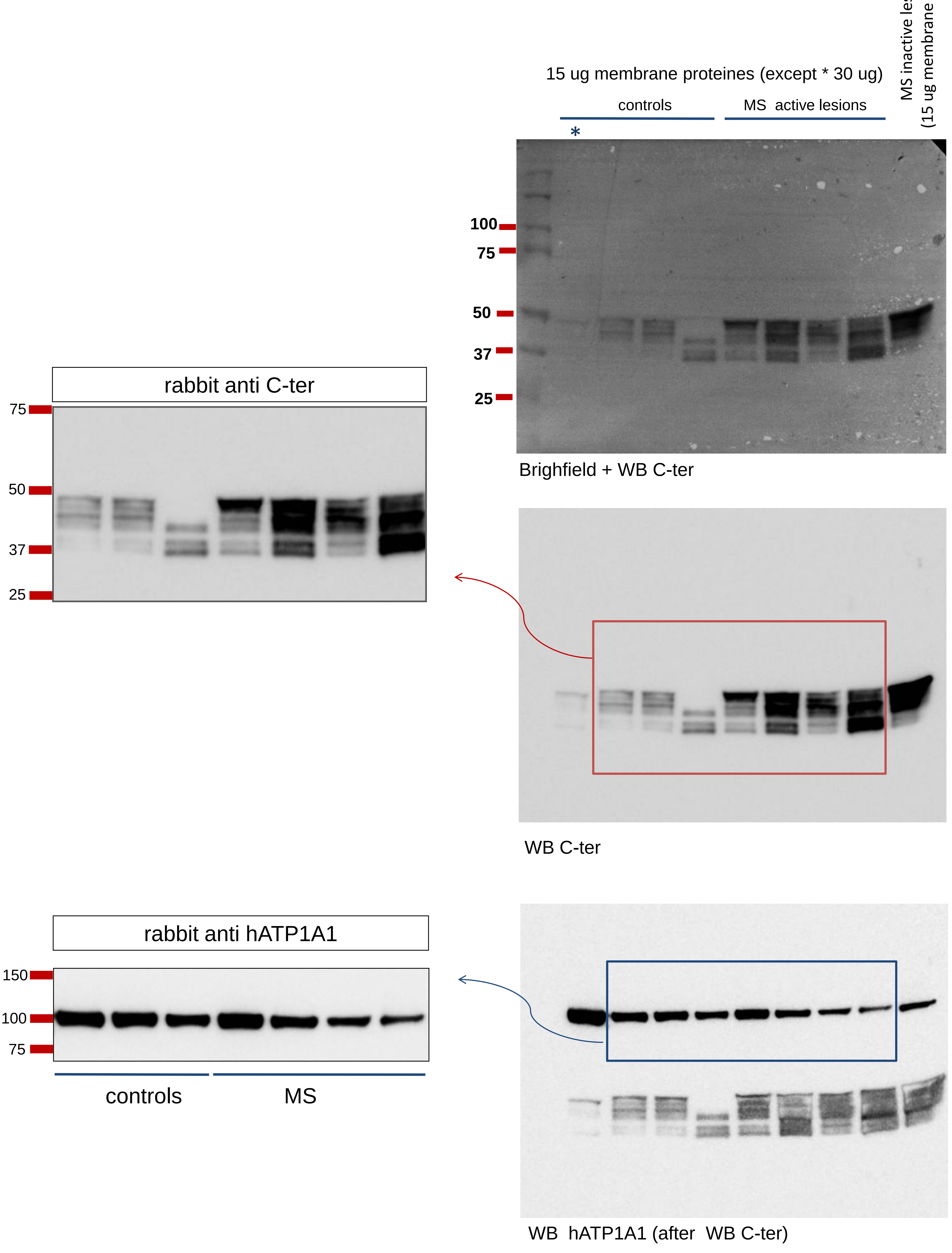

# unedited Western Blot
# for Figure 7D (Cter)

| | | | | | | | | | |
|---|---|---|---|---|---|---|---|---|---|
| | | | without PNGase | After PNGase F | without PNGase | After PNGase F | without PNGase | After PNGase F | After PNGase F |
| lane 1 | lane 2 | lane 3 | lane 4 | lane 5 | lane 6* | lane 7 | lane 8 | lane 9 | lane 10 |
| Marker | hCon+DTT | hCon-DTT | hCon+DTT | hCon+DTT | **infl**-DTT | **infl**-DTT | **infl**+DTT | **infl**+DTT | **infl**+DTT |

* lane 6: DTT spillover from neighboring lane 5

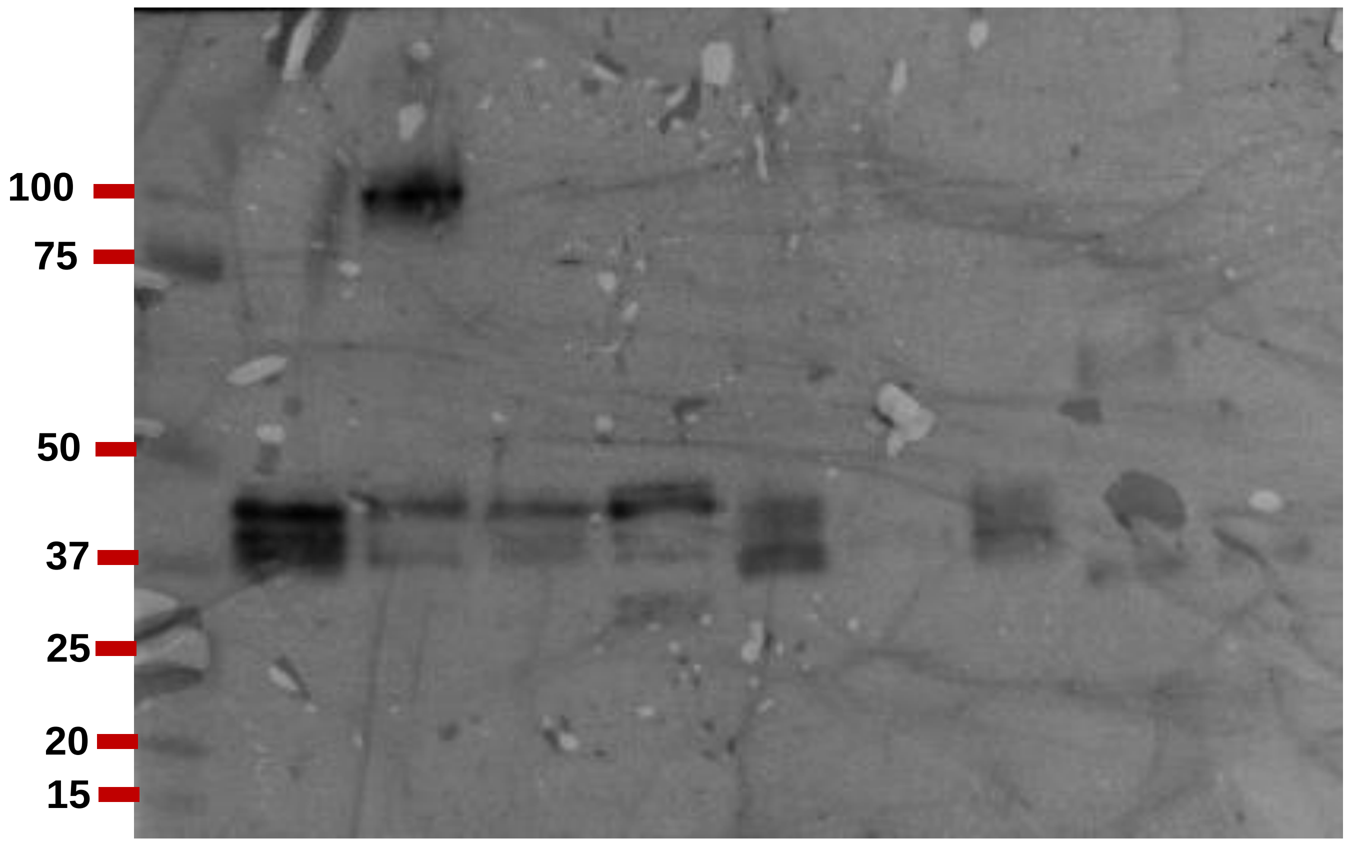


Brightfield + WB Cter (chemiluminescence)

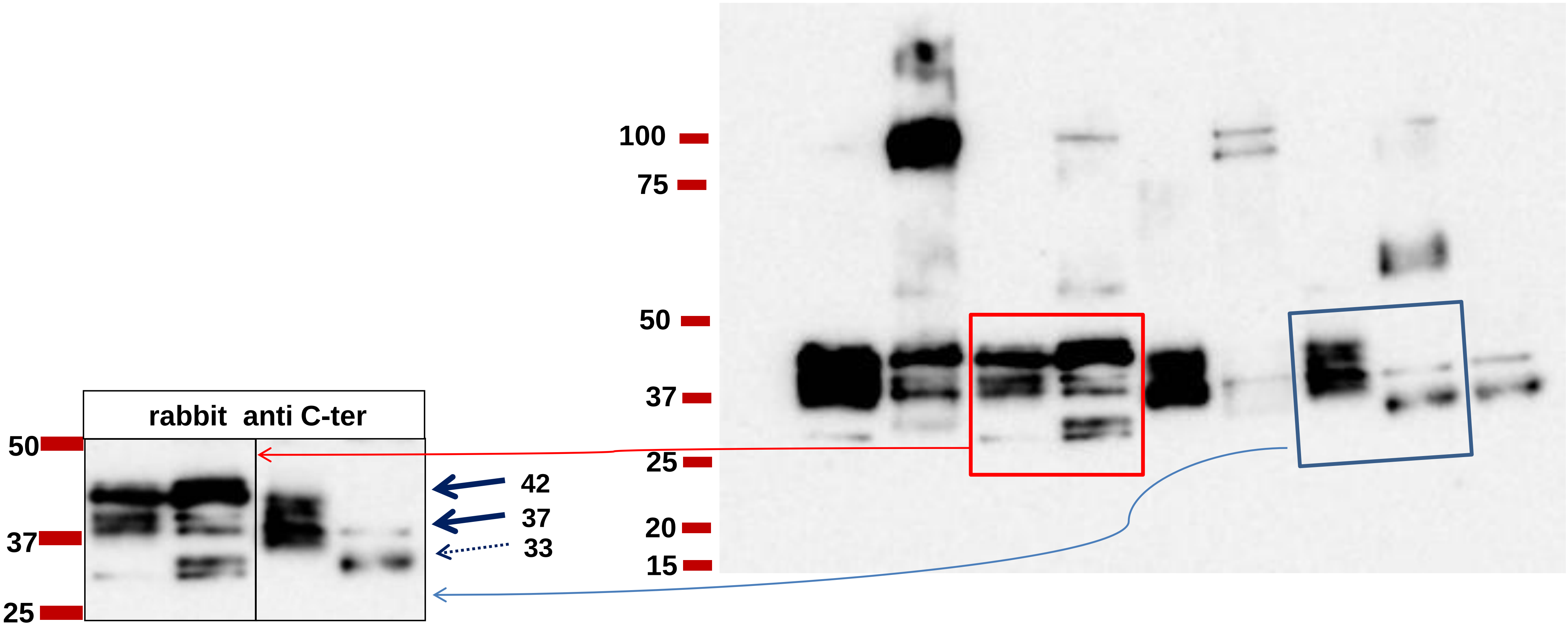


WB anti-Cter (chemiluminescence)

## unedited Western Blots for Figure 7D

| | | | | | | | | | |
|---|---|---|---|---|---|---|---|---|---|
| | | | without PNGase | After PNGase F | without PNGase | After PNGase F | without PNGase | After PNGase F | After PNGase F |
| lane 1 | lane 2 | lane 3 | lane 4 | lane 5 | lane 6* | lane 7 | lane 8 | lane 9 | lane 10 |
| Marker | hCon+DTT | hCon-DTT | hCon+DTT | hCon+DTT | infl-DTT | infl-DTT | infl+DTT | infl+DTT | infl+DTT |

* lane 6: DTT spillover from neighboring lane 5

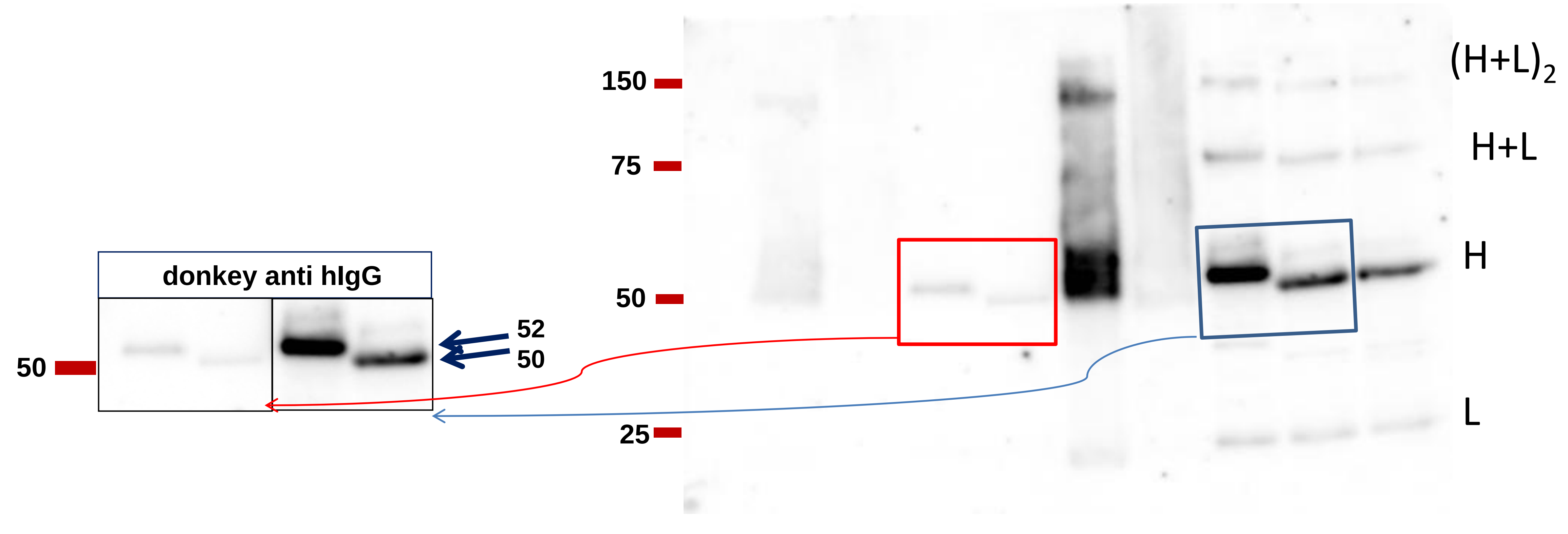


WB human IgG

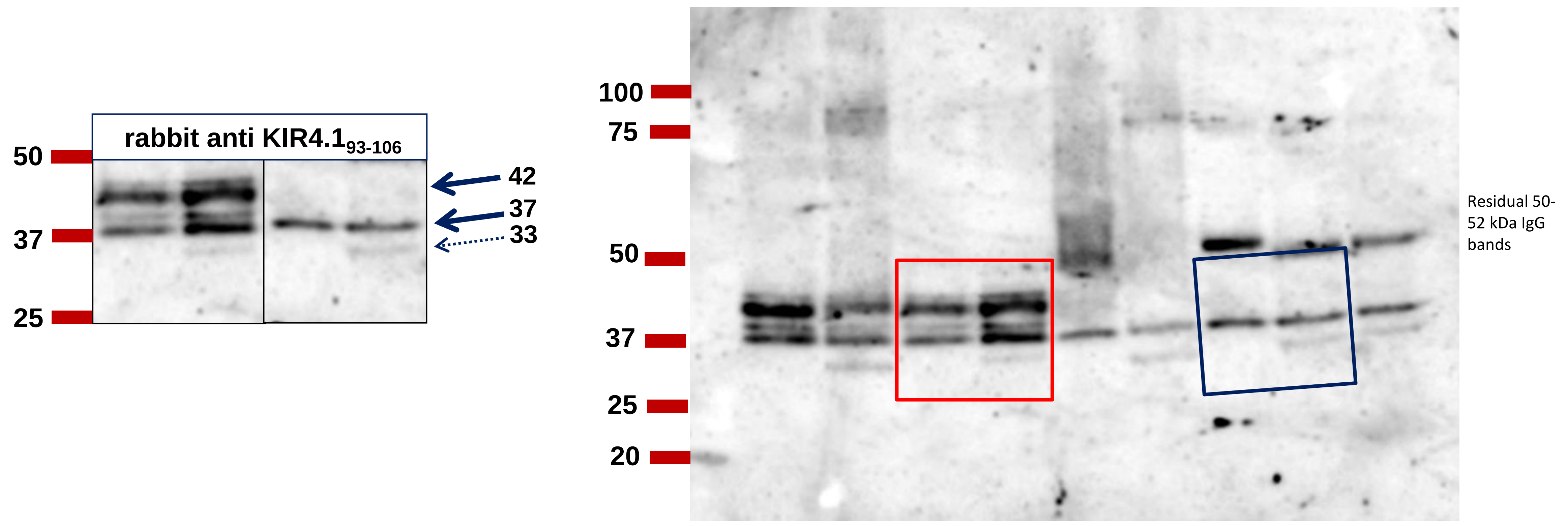


WB KIR4.1 $_{93\text{-}106}$ (after WB anti-human IgG)

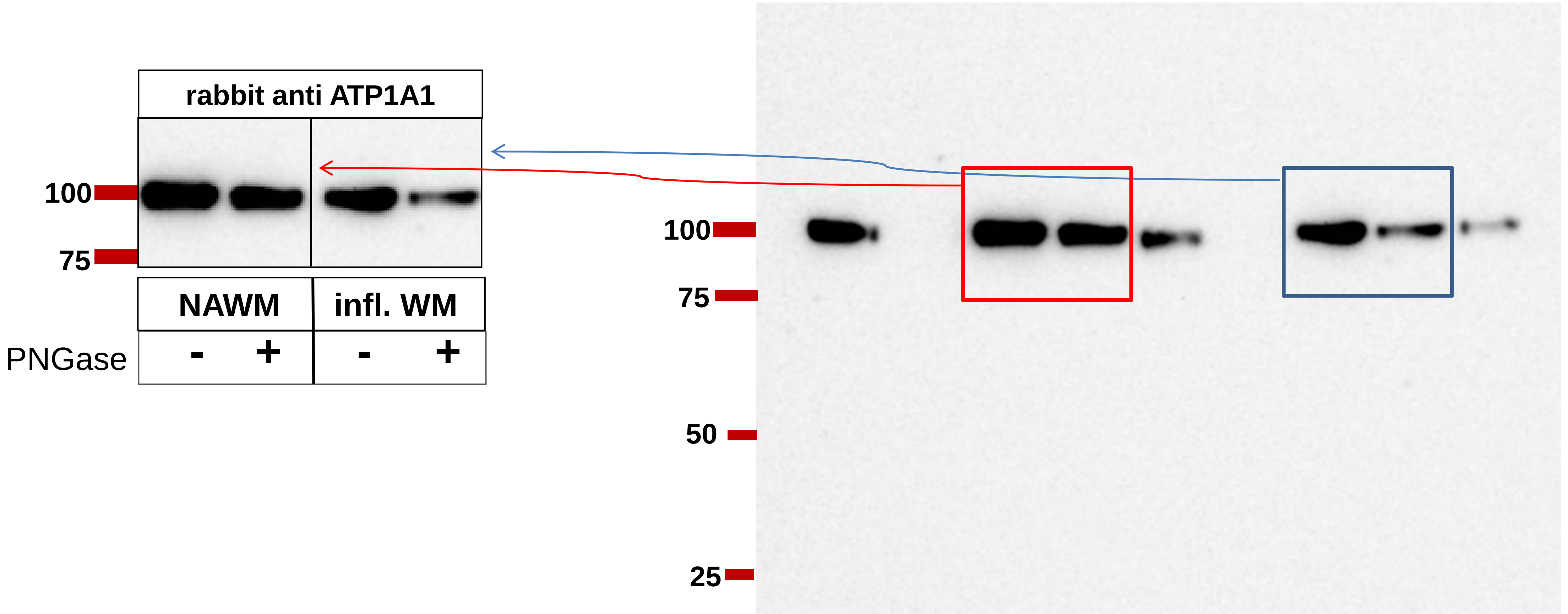


WB human ATP1A1 (denatured & reduced form only)

## unedited Western Blot
## for Figure 7E  (mouse anti e1)

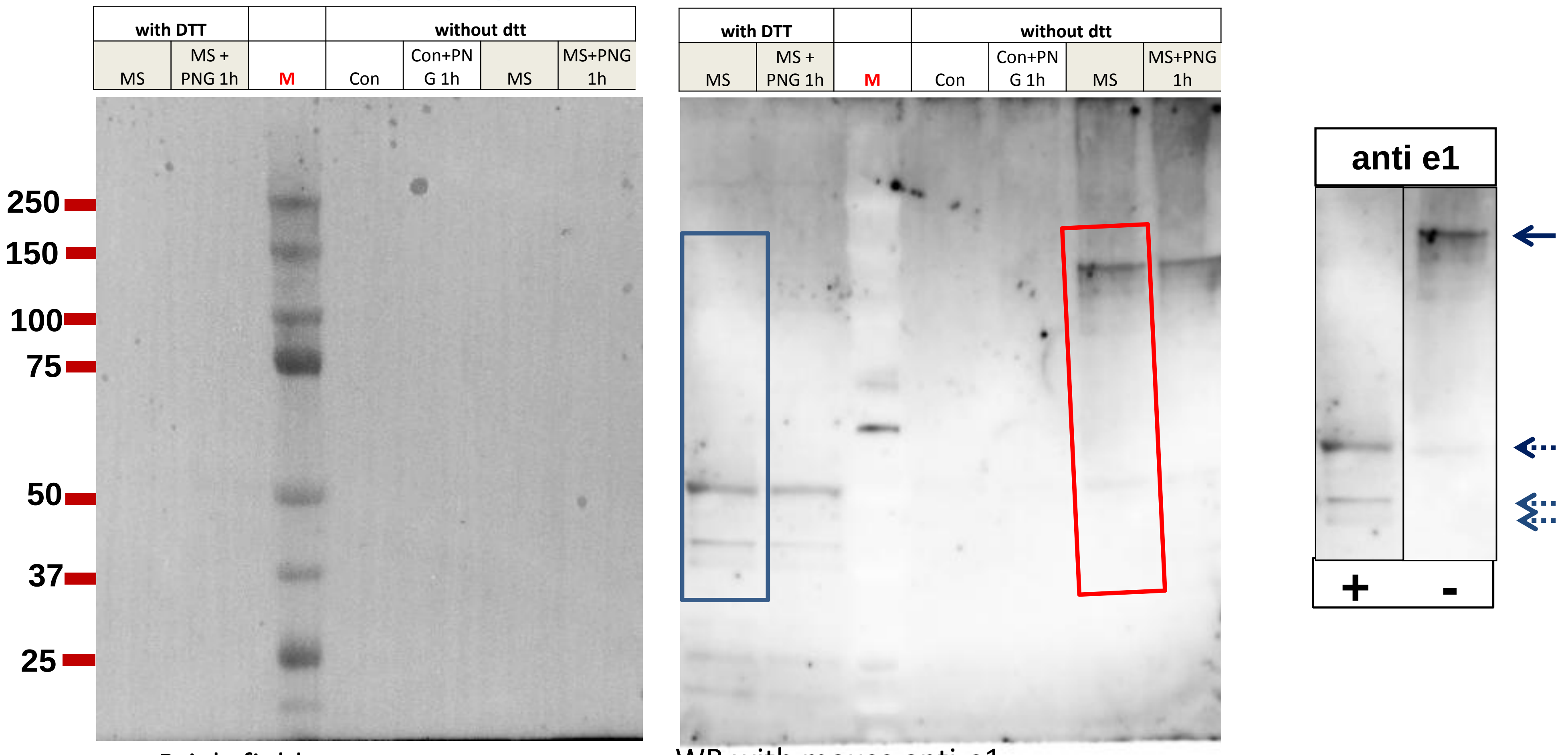


Brightfield

WB with mouse anti-e1

## unedited Western Blot
## for Figure 7E (C-ter antibody)

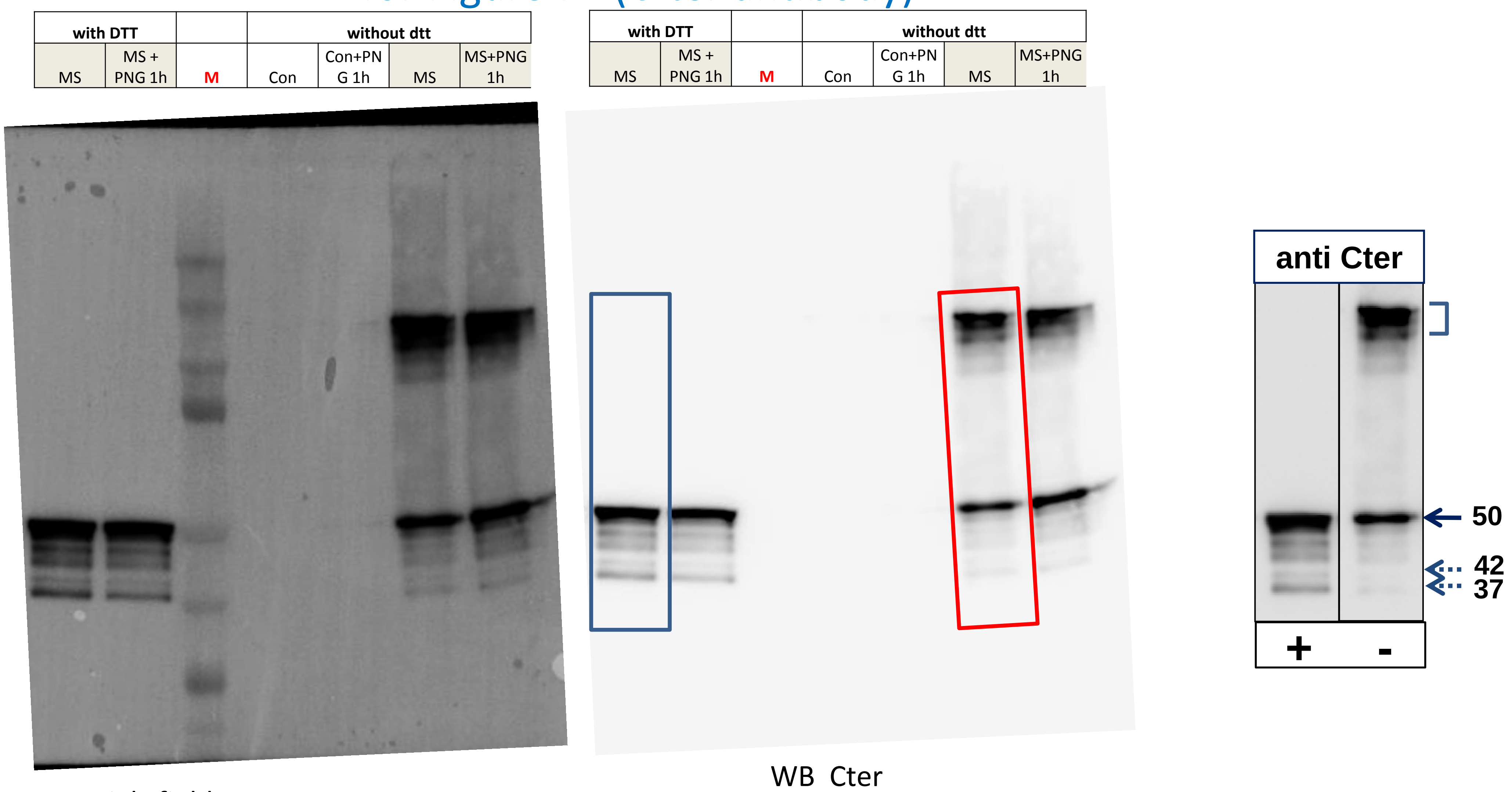


Brightfield  + WB  C-ter

WB  Cter

## unedited Western Blot
## for Figure 7E ( human IgG)

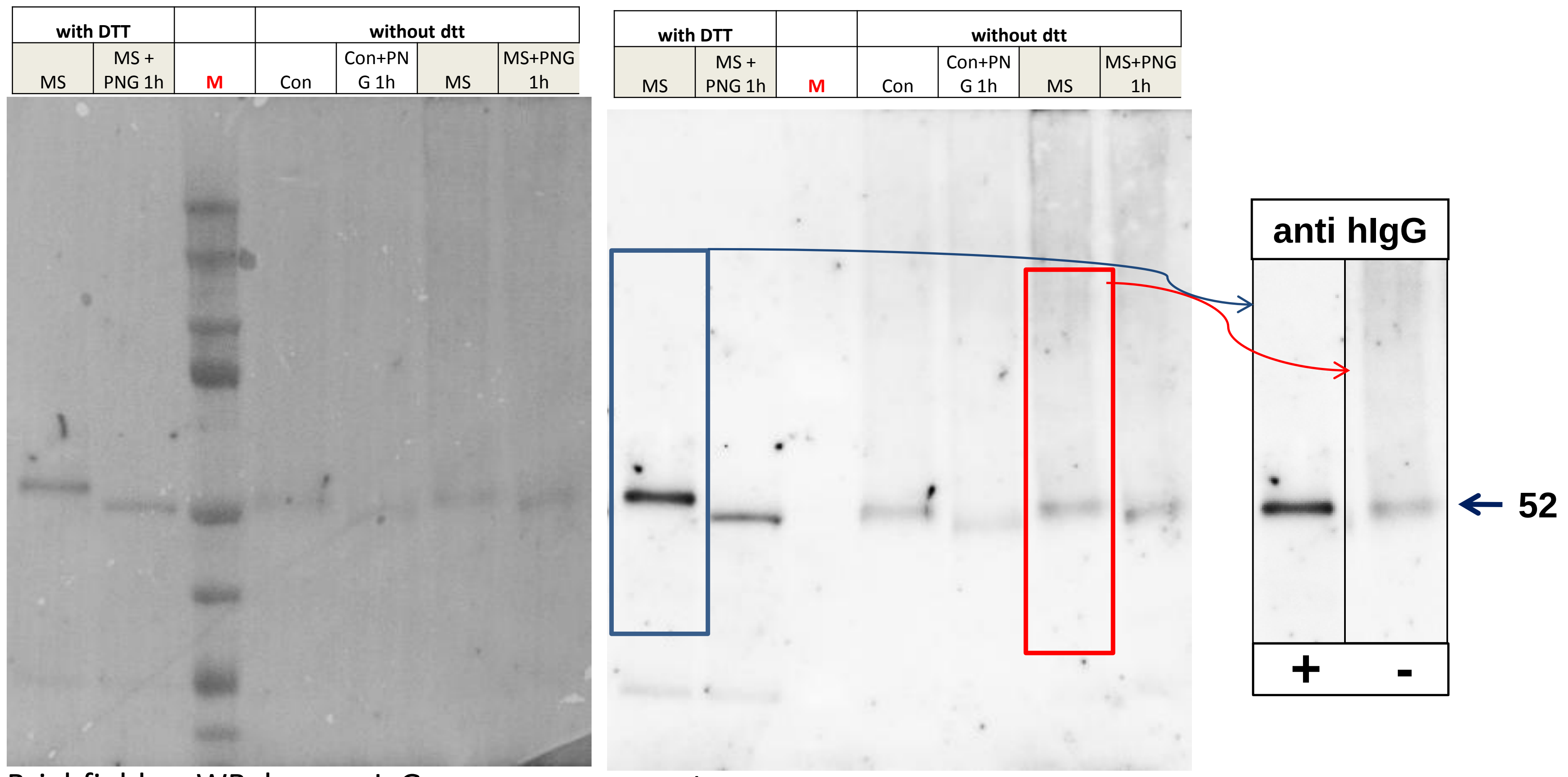


Brighfield  + WB  human IgG

WB  human IgG
