# Supplementary material for: Aglycosylated extracellular loop of inwardly rectifying potassium channel 4.1 (KCNJ10) provides a target for autoimmune neuroinflammation
Source: Brain Commun. 2023 Feb 22;5(2):fcad044. doi: 10.1093/braincomms/fcad044 (PMC9994600; doi:10.1093/braincomms/fcad044)
Supplement: fcad044_Supplementary_Data [file fcad044_supplementary_data.zip › Supplementary_data_files.pdf]

Supplementary : Kir4.1 e1 domain as autoimmune target

Arnaud B. Nicot[1,2], Jean Harb[1,2], Alexandra Garcia[1,2], Flora Guillot[1,2], Hoa-Le Mai [1,2], Camille V. Mathé[1], Jérémy Morille[1], Amélie Vallino[1], Emilie Dugast[1,2], Sita P. Shah[1], Fabienne Lefrère[3,4], Mélinda Moyon[3,4], Sandrine Wiertlewski[1,3,4], Ludmilla Le Berre[1,2], Karine Renaudin[1,2], Jean-Paul Soulillou[1], Vincent van Pesch[5], Sophie Brouard[1,2†], Laureline Berthelot[1†], David-Axel Laplaud[1-4]

Supplementary Figures 1 to 5

Supplementary Tables 1 to 5

Extended Methods

Supplementary Figures

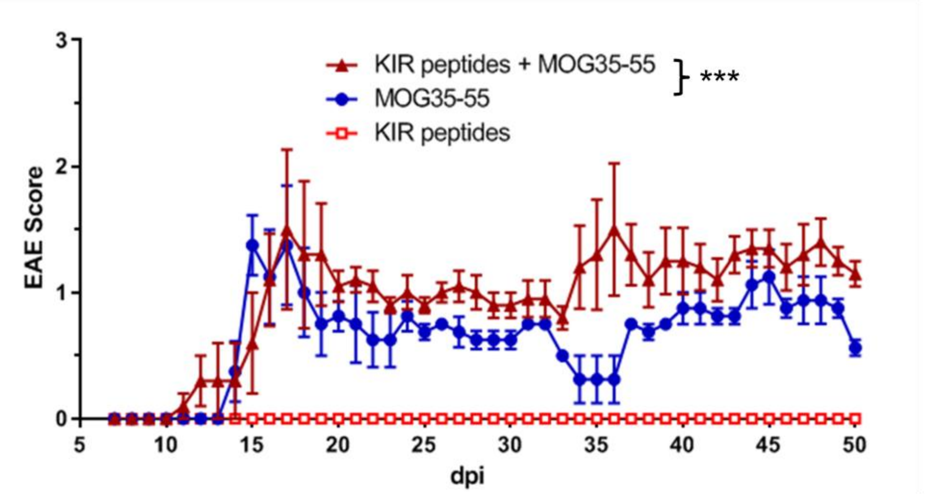

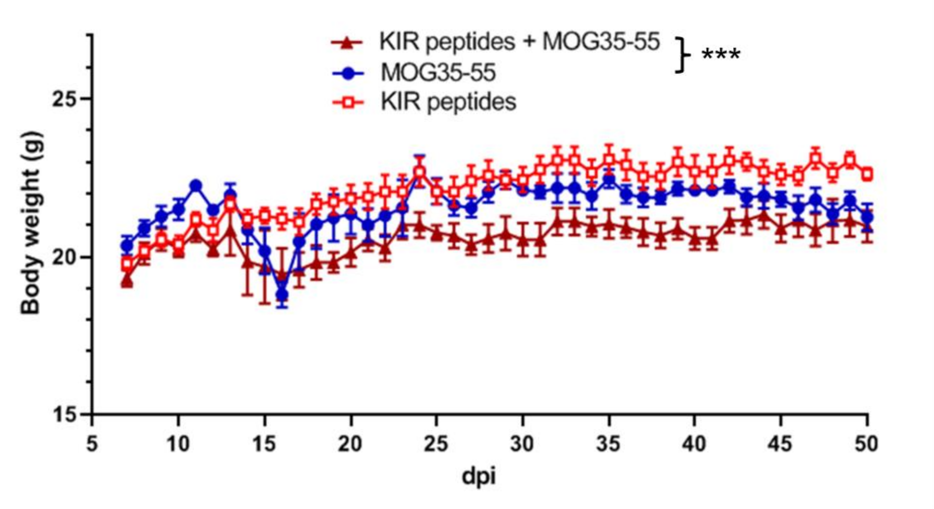

Supplementary Figure 1. Co-immunization with Kir4.1-specific peptides aggravates MOG_{35-55}- induced EAE. Top: EAE clinical score; **Bottom:** Corresponding weight (mean$\pm$SEM). A single immunisation with Kir4.1 peptides (including e1 peptide) does not induce neurological signs (n=5 mice) though it aggravates MOG_{35-55}-induced relapsing-remitting EAE. **dpi,** day post-immunization. Non-parametric repeated measures Friedman test was used. EAE score: group effect, F= 74.7, $P<0.0001$; overall difference between MOG35-55 (n=4) and Kir4.1 (KIR) peptides + MOG35-55 (n=5) groups, Dunn post hoc test: ***, $p<0.001$. Body weight: group effect, F= 73.8, $P<0.0001$; overal difference between MOG (n=4) and KIR +MOG35-55 (n=5) groups, post hoc Dunn test: ***, $p<0.001$. ***Induction of active MOG-EAE*:** five C57BL/6J female mice (eight weeks old, Enzo/Janvier Labs, France) were immunized once s.c. at the base of the tail into the lower flanks with a low dose (50 µg) of MOG35-55 peptide in phosphate buffer saline (PBS) emulsified with an equal part of complete Freund's adjuvant (CFA) supplemented with Mycobacterium tuberculosis H37Ra at 6 mg/ml (Difco Laboratories, Detroit, MI) injected s.c. into the flanks, followed by two injections of 300 ng pertussis toxin i.p at immunization and 48 h later. This protocol induced mild relapsing-remitting EAE in C57B6 mice as previously reported[1]. (Co-) immunization with KIR4.1-specific peptides: the encephalitogenic potential of Kir4.1-specific peptides was tested by immunizing B6 mice with one pool of seven Kir4.1 specific peptides including $e1_{83-120}$ (100 ug each, selected in the regions presenting the least homology with other channels) in 100 µl emulsion per mice) alone or in combination with 50 µg $MOG_{35-55.}$ Peptides (purity >85%, Genecust Europe, France) were kept frozen at -80°C until use. Investigators have been blinded to the sample group allocation during clinical score recording.

1. Berard JL, Wolak K, Fournier S, David S. Characterization of relapsing-remitting and chronic forms of experimental autoimmune encephalomyelitis in C57BL/6 mice. *Glia* **58**, 434-445 (2010).

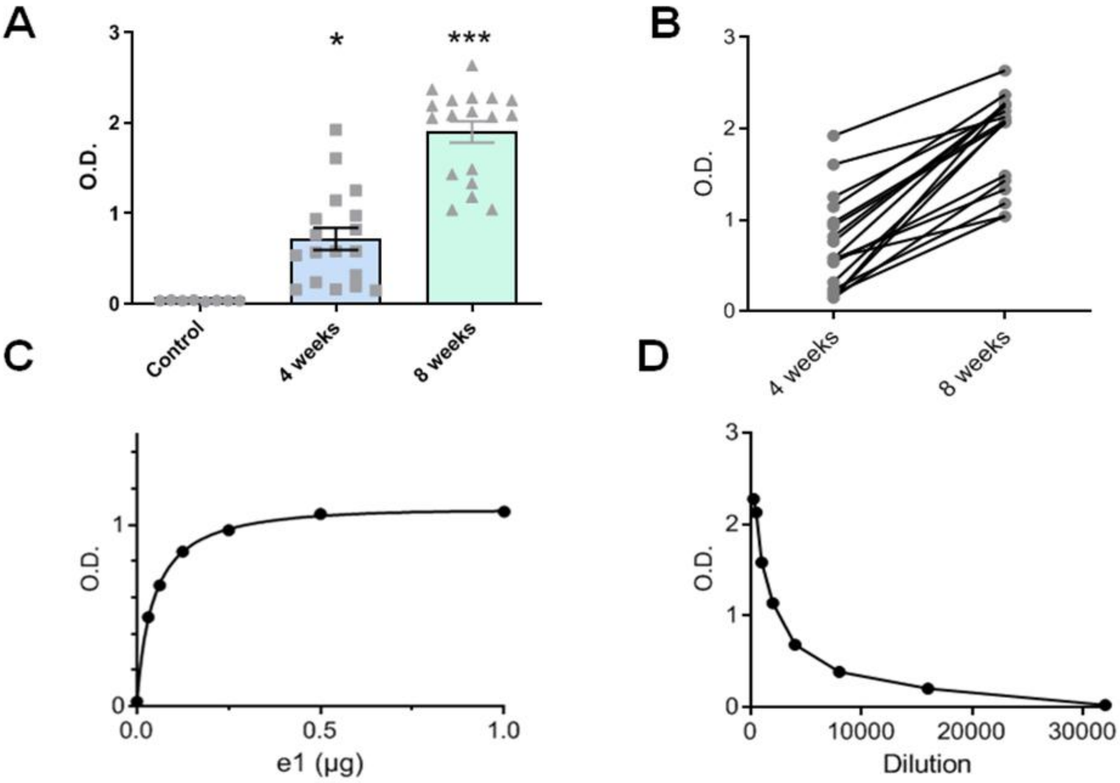

Supplementary Figure 2. Mice immunized with e1 peptide develop high serum anti-e1 IgG levels. (**A**) Anti-e1 IgG from sera (1/500) of PBS/IFA immunized mice (controls, *n*=8) vs. e1-immunized mice (4 and 8 weeks post-immunization, *n*=18 each, mean $\pm$ SEM). Kruskal-Wallis non-parametric test, Dunn's post hoc test *vs* Control: *, $p<0.05$; ***, $p<0.0001$. (**B**) Corresponding values at 4 *vs.* 8 weeks. (**C**) Effect of increasing e1 peptide coating on anti-e1 IgG detection with serum pooled from five e1-immunized mice at 8 weeks (tested at 1/2000), representative curve from simplicates. (**D**) Effect of decreasing serum concentration on anti-e1 detection using the same serum pool (background level<0.05), representative curve mafrom simplicates. O.D., Optical density.

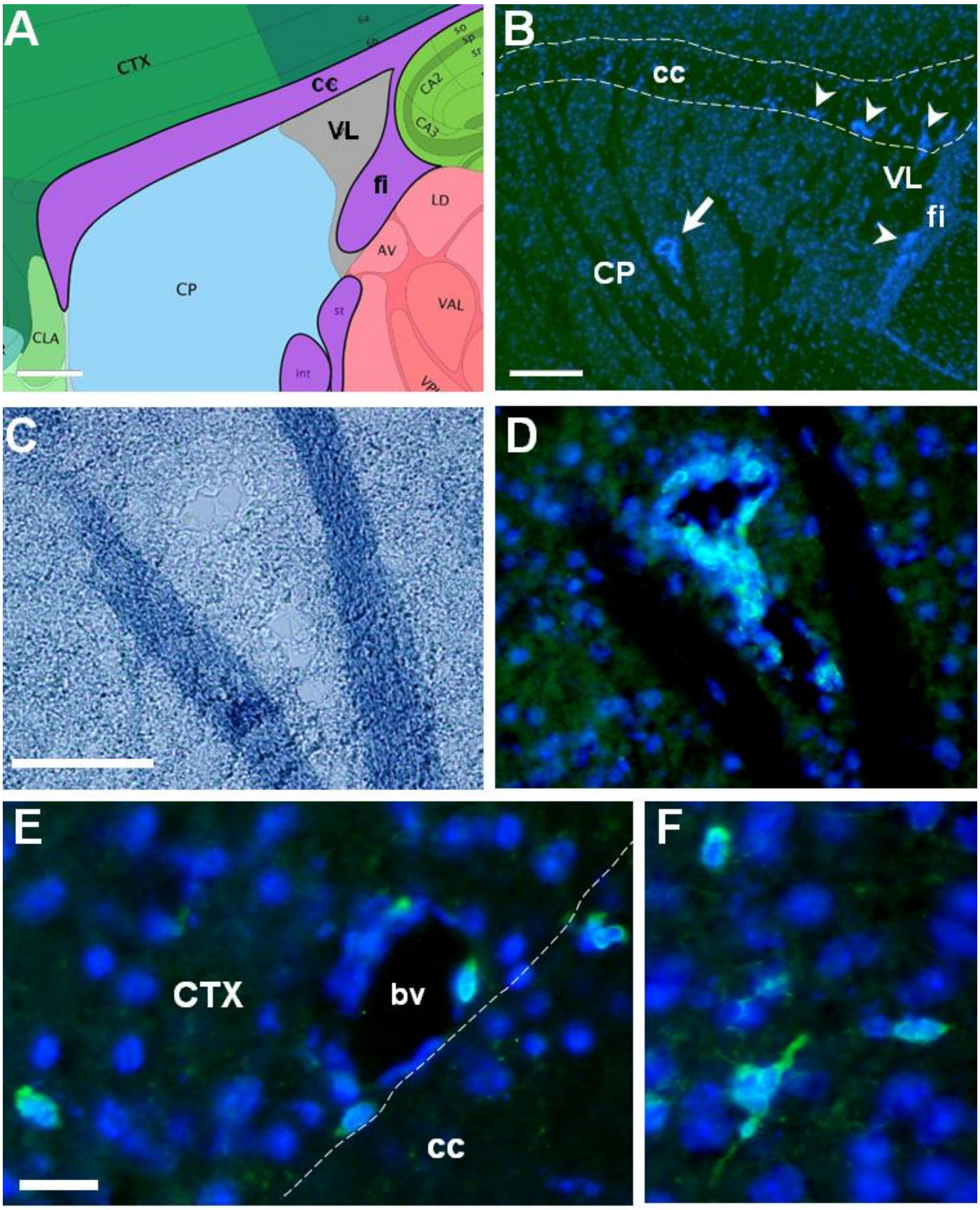

Supplementary Figure 3. Limited immune cell infiltration in the forebrain gray matter of symptomatic e1-immunized mice. (**A**) Schematic view of a forebrain sagital section used for b. Reference Atlas image 20, P56, sagital; Image credit: Allen Institute. Scale bar, 400 µm. (**B**) Corresponding field of DAPI blue stain from a symptomatic e1-immunized mouse, showing patches of cell infiltrates in white matter tracts (arrowheads) or of perivascular cells in the striatal gray matter (arrow). Scale bars a-b, 400 µm. Sudan black myelin stain (**C**) and corresponding field (**D**) with DAPI stain (blue) and CD3 immunostaining (green) showing perivascular cell infiltrate in the striatal graymatter. Some B cells invade the bordering thalamus and stria medularis (arrows). Scale bar c-d, 80 µm. (**E, F**) DAPI stain with CD3 immunostaining (green) in the somatomotor (**E**) or occipital (**F**) cortex from a symptomatic e1-immunized mouse. Scale bar for E and F, 40 µm. Legends: CP, Caudate Putamen; CTX, cortex; bv, blood vessel; cc, corpus callosum; fi, fimbria; VL, lateral ventricle.

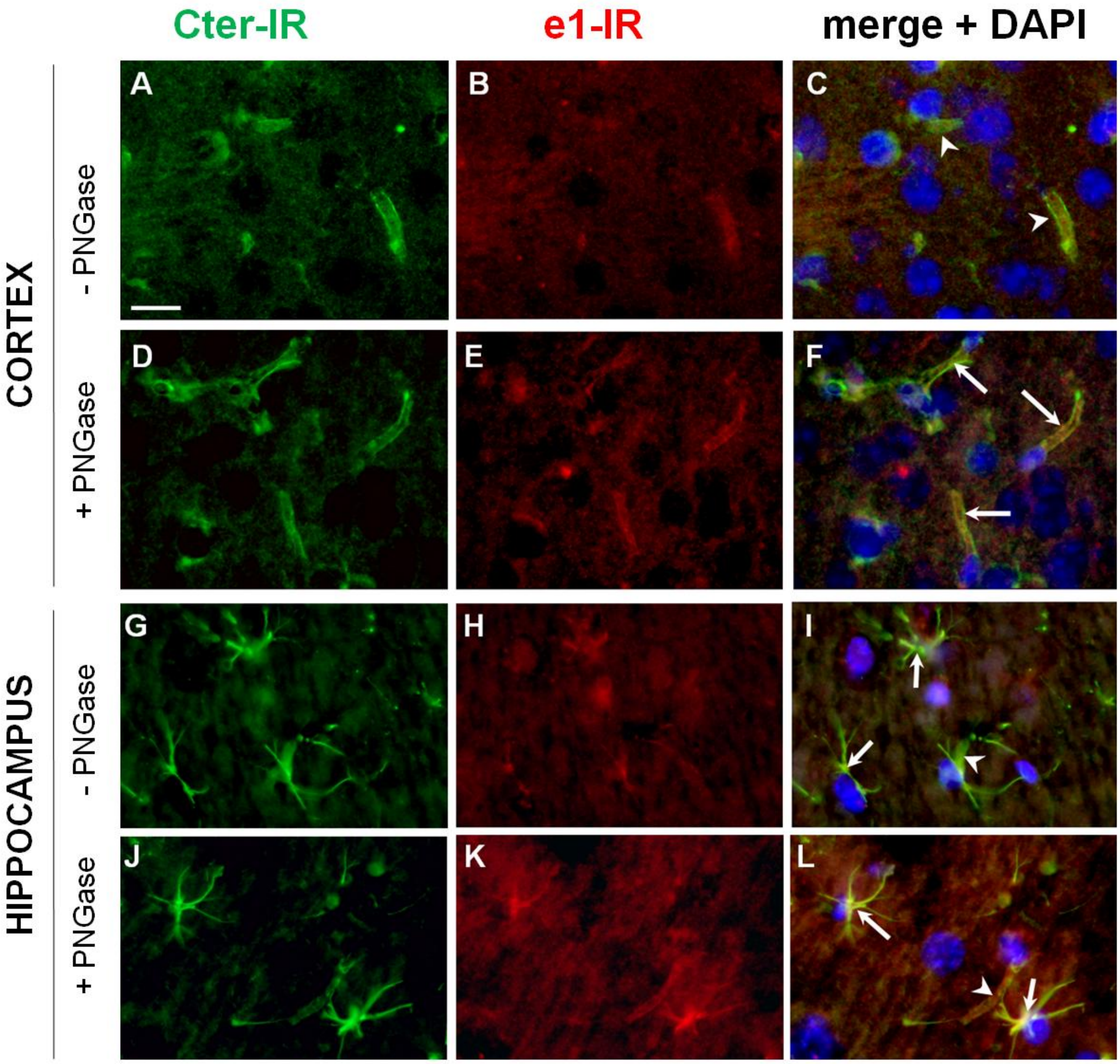

Supplementary Figure 4. PNGase treatment increases astrocytic endfeet anti-e1 immunoreactivity (IR) in normal mouse brain. Representative mouse cortical (**A to F**) or hippocampal (**G to L**) sections without (**A to C, G to I**) or with (**D to F, J to L**) PNGase treatment. Without PNGase treatment, the anti-Cter labels the blood vessel astrocytic endfeet (arrowheads) and some hippocampal stellate astrocytes (arrows). After PNGase treatment, e1 labeling increases and co-localizes with Cter staining. First column: immunoreactivity with anti-Cter Kir4.1 antibody (green). Second column: immunoreactivity with anti-e1 mouse serum (red). Third column: Corresponding merge fields with DAPI stain. When the primary rabbit or mouse antibody was omitted, no green or red signal was seen at the exposure time used (not shown). Scale bar for A to L, 10 µm.

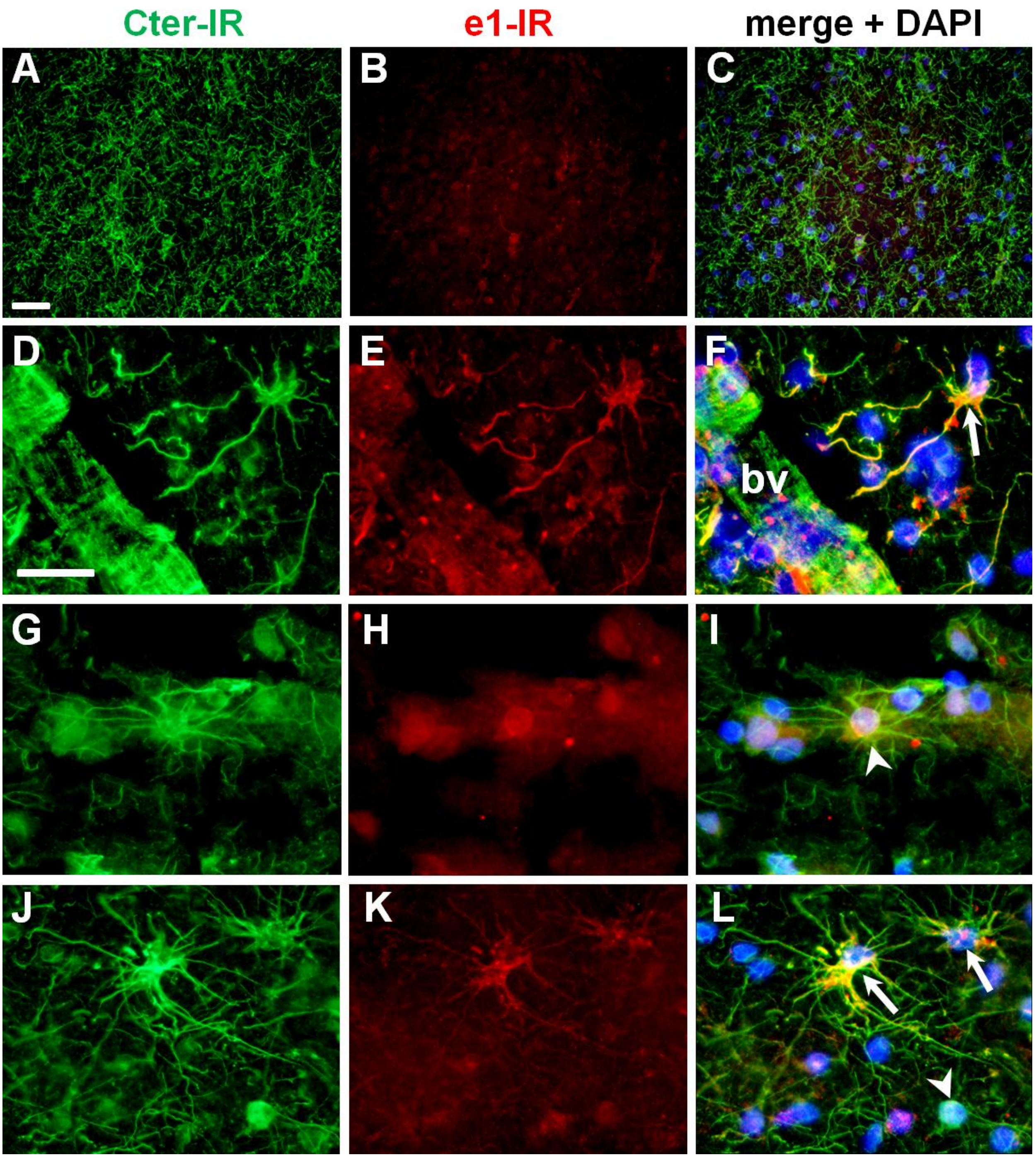

Supplementary Figure 5. Kir4.1-immunoreactivity (IR) on human brain using rabbit anti-Cter antibody and mouse anti-e1 serum. (**A to C**) Subcortical NAWM at low magnification showing Cter Kir4.1-IR glial fibers but barely detectable e1(aa 83-120) immunoreactivity in scarce glial cells/fibers. Scale bar A-C, 40 µm. (**D to L**) Examples at high magnification of the co-stainings obtained from a control brain. Scale bar for D to L, 40 µm. **First column,** Cter immunofluorescence (green); **Second column,** e1-immunofluorescence (red); **Third column,** merged signals with DAPI stain and with enhanced red channel contrast to better visualize co-localization. (**D to F**) Perivascular astrocyte (arrow) apposing its endfoot to a blood vessel (bv). (**G to I)** labeled oligodendria (arrowhead). (**J to L)** labeling of two white matter astrocytes (arrows) and one oligodendrocyte-like cell (arrowhead in L). In control human brain, the e1-immunoreactivity indeed appears mainly present around the nucleus and proximal processes, while Cter Kir4.1 immunoreactivity is also well detected all along glial fibers.

Supplementary Tables

Supplementary Table 1: Profile of the 15 identified HR patients at time of blood sampling.

Clinical profile of patients with high anti-e1 reactivity ("HR"), neurological disorders presented at CIS and MRI-guided lesion localization were retrieved from the Hospital databanks.

	Age	Sexe	Type	relapse at blood sampling	disease duration	EDSS	Treatment	MRI localization	Epilepsy during childhood	
HR1	73	F	PP	No	5	6	none	weak: periventricular, pons/brainstem, spinal cord	No	
HR2	22	M	RR	No	8	0	Avonex	periventricular	No	
HR3	45	F	RR	No	8	1	none	left pseudo-tumoral lesion	No	
HR4	62	F	RR	No	19	2.5	none	not determined	No	
HR5	33	F	RR	No	6	1.5	Copaxone	periventricular	No	
HR6	30	M	RR	No	9	0	none	periventricular (lateral and IV ventricles)	No	
HR7	64	F	SP	No	11	9	none	atypical and weak at spinal cord C2-T1 + one periventricular lesion (LV)	No	
HR8	40	F	RR	Yes	2	1	Copaxone	numerous lesions, Balo's type, pseudo-tumoral	No	
HR9	39	F	RR	No	15	1	none	cervical spinal cord + periventricular	Yes	
				**neurological signs**						**MS conversion**
HR10	52	F	CIS	vertigo, diplopia				brainstem	No	RR
HR11	32	M	CIS	optic neuropathy				periventricular and juxtacortical	No	RR
HR12	36	F	CIS	diplopia				brainstem, spinal cord	No	RR
HR13	27	F	CIS	vertigo, diplopia, Right hypersensitivity				periventricular and one cerebellar lesion	No	RR
HR14	36	F	CIS	optic neuropathy				periventricular and juxtacortical	No	RR
HR15	38	M	CIS	Left hypersensitivity				periventricular and juxtacortical	No	RR

Supplementary Table 2. Antibodies used for immunohistofluorescence

Target	Marker	Host species	Concentration	Provider	Reference
CD3	Pan T-cell	Rat	1:500	BD Biosciences	555273
CD45	Immune cells	Rat	1:200	BD Biosciences	550539
B220/CD45R	B cell lineage	Rat	1/200	BD Biosciences	553084
mouse IgG	Immunoglobulins & B cell lineage	Donkey	1:1500 (AF594-labeled Fab'2)	Jackson ImmunoResearch	715-586-150
Iba1	Microglia/Macrophages	Rabbit	1:2000	Proteintech Europe	10904-1-AP
F4/80	Microglia/Macrophages	Rat	1/1000	BioRad/AbD Serotec	Ab6040
Ly6G	Granulocytes	Rat	1/200	BD Biosciences	551459
GFAP	Astrocytes and CNS parenchyma border	Rabbit	1/1000	Dako	Z0334
$Kir4.1_{356\text{-}375}$ Cter	Glia	Rabbit	1/800	Alomone	APC-035
Kir4.1 ($e1_{83\text{-}120}$)	Glia	Mouse	1/400	serum (2 months of immunization)	this article
rabbit IgG	secondary	Donkey	1:1500 (AF488-labeled Fab'2)	Jackson ImmunoResearch	711-546-152
rat IgG	secondary	Donkey	1:1500 (AF594-labeled Fab'2)	Jackson ImmunoResearch	712-586-153

Supplementary Table 3 Characteristics of patients from whom snap-frozen autopsy CNS tissues were used for immunofluorescence or Western Blot studies.

Patient	Age	Sex	Disease duration (years)	Disease type	EDSS	Death-sampling interval (h)	Cause of death
MS 1	45	M	12	MS (SP)	8.5	12	Lung cancer
MS 2	66	F	10	MS (PP)	7.5	6	Pulmonary infection
MS 3	54	F	23	MS (PP)	9	8	Pulmonary infection
MS 4	59	F	19	MS (SP)	9	8	Pulmonary infection
Con	55	M	20	Control	-	12	lymphoma (brain not affected)
INF	34	M	0.5	Acute lethal encephalitis	-	18	Encephalitis/cranial hypertension

Supplementary Table 4: Subcortical sections used for Kir4.1 immunofluorescence.

Sample	WM lesion type	CD68 staining	Demyelination	Block
Con	NAWM	weak/ponctuate	No	myelin
MS2	NAWM	weak/ponctuate	No	PDLF1
MS3	NAWM	weak/ponctuate	No	C7P3
MS3	NAWM	weak/ponctuate	No	C7P4
MS1	WM Active lesion	++	Yes	SB4
MS2	WM Active lesion	++	Yes	LB
MS3	WM Active lesion	++	Yes	C3B2
MS4	WM Active lesion	++	Yes	CM14
INF	Perivascular & WM immune cell infiltration	++	limited	SB1-1

Supplementary Table 5. Brain samples used for WB study

Sample	Region	Inflammation	Demyelination	Block
NAWM1	Subcortical NAWM	No	No	PDLF1
NAWM2	Subcortical NAWM	No	No	C7P3
NAWM3	Subcortical NAWM	No	No	C7P4
MS1	Active subcortical WM lesion	Yes	Yes	SB4
MS2	Active subcortical WM lesion	Yes	Yes	LB
MS3	Active subcortical WM lesion	Yes	Yes	C3B2
MS4	Active subcortical WM lesion	Yes	Yes	CM14
Con	Subcortical NAWM	No	No	myelin
INF	Inflamed Subcortical WM	Yes	limited	SB1-1

Extended Methods

Murine model of Kir4.1(e1)-induced autoimmune encephalomyelitis

Eight to ten weeks-old C57BL6/J female mice (Janvier/Enzo Labs) were immunized with 100 µg of e1 peptide (sequence 83-120 of Kir4.1, purity >85%, Covalab) emulsified with an equal volume of Incomplete Freund Adjuvant (IFA, Difco Laboratories) *s.c.* into the lower flanks (100 µl divided equally) and followed by two more weekly injections of 50 and 25 µg e1 in IFA emulsion upper into the flanks. For control animals, the peptide solution was replaced with saline. One month after the first e1 boot, all mice were challenged *s.c.* with 100 µl of complete Freund's adjuvant (CFA, with 6 mg/mL heat killed dried M. tuberculosis, strain H37Ra) emulsioned with PBS, and injected immediately and 48 h after with 300 ng *i.p.* of pertussis toxin (PTX, Calbiochem). Mice were scored daily for typical experimental autoimmune encephalomyelistis (EAE) signs according to the standard scale: 0, no detectable sign; 1, complete limp tail; 2, limp tail with unilateral hindlimb paralysis; 3, bilateral hindlimb paralysis; 4, bilateral hindlimb paralysis and forelimb weakness (end point). An additional score +1 was added to the typical EAE score when the following clinical signs of atypical EAE were observed: kyphosis/hunch appearance with stiffness/spasticity, head tilting, one-side shifted locomotion and/or freezing.

At the end of the experiment, five mice that have shown atypical and typical EAE, two non symptomatic mice and three PBS/CFA controls were anesthetized with thiopental and intracardiacally perfused with cold PBS followed by 4% paraformadehyde in 0.1 M Phosphate buffer pH 7.4. The dissected spinal cords and brains were post-fixed and sucrose-protected for three days at 4°C. Spinal cords embedded in Tissue-Tek® OCT and brains were frozen in crushed dry ice and stored at -80°C until cutting. In order to measure anti-e1seric IgG, blood sampling was performed at the sinus (200 µl) at one and two months after the first immunization. All animals were acclimated 10 days to animal facility before use. Animals were housed 4 to 5 per cage. Once the animals were sick, easier access to water and softened food was given every morning in the bottom of the cage in a 100 mL plastic weighing boat.

Mouse T cell proliferation assay and ELISPOT

Female C57BL/6 mice were immunized with three weekly s.c. injection of e1 (n = 5) or PBS (n = 5, controls) in IFA and were sacrificed two weeks after the last injection. Blood was collected by cardiac puncture and sera were kept at -20°C until anti-e1 IgG assay. Spleens were harvested and digested with collagenase D for 20 min at 37°C (Roche). To evaluate T cell proliferation in response to e1 peptide, dissociated splenocytes were labeled with cell proliferation dye (CPD)eFluor670 (Thermofisher) according to the manufacturer's instruction and seeded at $2x10^5$ cells/well in a 96-well U-bottom plate and cultured for 3 days in the presence of e1 (20 µM) or vehicle (DMSO) only. Cells were then harvested, washed, resuspended at 10^6 cells/ml in PBS, stained with fixable viability dye (FVD) eFluor450 (Thermofisher) for 30 min, and washed twice with PBS containing 2% BSA. Cells were then stained with FITC-conjugated anti-CD3 (clone 145-2C11) and PE-Cy7-conjugated anti-CD4 mAbs (clone RM4-5) for 15 min, washed twice, and resuspended in PBS containing 2% BSA for acquisition. About 20,000 events were acquired for each sample using an LSRII flow cytometer (BD Biosciences) and data were analyzed with the FlowJo software (v10, TreeStar). After eliminating doublets (FSC-H/FSC-A), cell debris (FSC/SSC), and dead cells (FVD+), CD3+CD4+ cells were gated and the percentages of CPD-low cells/total CD3+CD4+ cells were reported.

Amplified e1-specific memory T cells were assessed for IFN-γ release using the mouse IFN-γ ELISPOT Set (BD Biosciences) according to the manufacturer's instructions. Briefly, a 96-well ELISPOT plate was coated overnight with the anti-mouse IFN-γ capture antibody, washed, and blocked for 2h with complete medium. Dissociated splenocytes were incubated for 18h at 37°C in duplicate at $2x10^5$ cells/well in complete medium with e1 (20 µM) or vehicle (DMSO) only. After incubation with a biotinylated anti-mouse IFN-γ antibody followed by streptavidin-HRP, the wells were washed with PBST, and incubated with 3-Amino-9-EthylCarbazole substrate for 5 min. After washing and overnight air-drying, the plate was analyzed using an AID-EliSpot reader (Autoimmun Diagnostika GmbH) with the AID EliSpot reader software V7.0.

Histoneuropathology in e1-immunized mice

Histological evaluation was done with 14 µm thick sagital sections of the brain and coronal sections of the cervical, thoracic and lumbar spinal cord. Sections were stained with DAPI for nuclear identification and Black

Sudan to evidence demyelination foci of inflammation in four to six sections (140 µm apart) per CNS region (forebrain/cerebellum or spinal cord) per mouse. Histological evaluation for inflammation and demyelination was done “blinded”. For immunofluorescence, sections were subjected to heat antigen retrieval in 10 mM Na-Citrate buffer (pH 6.0) at 90°C, preincubated in PBS with 25% Normal Donkey Serum (NDS) and 0.03% Triton-X100 for 20 min at room temperature, followed by overnight incubation at 4°C with primary rat or rabbit antibodies to immune cell markers and GFAP (Supplementary Table 2) in PBS, 2.5% NDS and 0.03% Triton-X100. After three washes in PBS, sections were incubated for 45 min with donkey AF594- or AF488-coupled F(ab')2 anti-mouse, anti-rat or anti-rabbit IgG (1:1500) in PBS, 2.5% NDS and 0.03% Triton-X100. NeuroTrace™ 530/615 red fluorescent Nissl stain (ThermoFisher) was used to examine potential alterations in the Purkinje layer. Sections were stained with DAPI and coverslipped with anti-fading mounting medium (Mowiol/DABCO) and analyzed on a fluorescent microscope using appropriate filters (Nikon). Representative pictures were taken at x20 or x40 objective using a digital camera (Eclipse DXM1200) connected to image-acquisition software (ACT-1, Nikon). Mouse atlas reference images were obtained from the Allen Institute website at www.alleninstitute.org.

Mouse T cell proliferation assay and ELISPOT

Female C57BL/6 mice were immunized with three weekly s.c. injection of e1 (n=5) or PBS (n=5, controls) in IFA and were sacrificed two weeks after the last injection. Blood was collected by cardiac puncture and sera were kept at -20°C for measurement of anti-e1 IgG levels. Spleens were harvested and digested with collagenase D for 20 min at 37°C (Roche) After splenocyte dissociation, living cells were counted using Malassez counting chamber and 0.1% Erythrosin B exclusion. To evaluate T cell proliferation in response to e1 peptide, splenocytes were labeled with cell proliferation dye (CPD) eFluor670 (Thermofisher) according to the manufacturer's instruction and seeded at $2x10^5$ cells/well in a 96-well U-bottom plate and cultured for 3 days in the presence of e1 (20 µM) or vehicle (DMSO) only. After 3 days of culture, cells were harvested, washed, resuspended at 10^6 cells/ml in PBS, stained with fixable viability dye (FVD) eFluor450 (Thermofisher, Cat N°65-0863) for 30 min, and washed twice with PBS containing 2% BSA. Cells were then stained with FITC-conjugated anti-CD3 (clone 145-2C11, Cat N° 553062) and PE-Cy7-conjugated anti-CD4 mAbs (clone RM4-5, Cat N° 552755) (both from BD Biosciences) for 15 min, washed twice, and resuspended in PBS containing 2% BSA for acquisition. About 20,000 events were acquired for each sample using an LSR II flow cytometer (BD Biosciences) and data were analyzed with the FlowJo software (v10, TreeStar). After eliminating doublets (FSC-H/FSC-A), cell debris (FSC/SSC), and dead cells (FVD+), CD3+CD4+ cells were gated and the percentages of CPD-low cells/total CD3+CD4+ cells were reported.

Amplified e1-specific memory T cells were assessed for IFN-γ release using the mouse IFN-γ ELISPOT Set (BD Biosciences, Cat N° 551083) according to the manufacturer's instructions. Briefly, a 96-well ELISPOT plate (Millipore, #S2EM004M99) was coated overnight with the anti-mouse IFN-γ capture antibody, washed, and blocked for 2h with complete medium. Splenocytes were suspended at 10^6 cells/ml in complete medium, seeded in duplicate at $2x10^5$ cells/well with e1 (20 µM) or vehicle (DMSO) only, and incubated for 18h at 37°C. After incubation with a biotinylated anti-mouse IFN-γ antibody followed by streptavidin-horse radish peroxidase, the wells were washed with PBS containing 0.05% Tween 20, and incubated with 3-amino-9-ethylcarbazole (AEC) substrate for 5 min. The reaction was stopped by washing with deionized water. After overnight air-drying, the plate was analyzed using an AID-EliSpot reader (Autoimmun Diagnostika GmbH) with the AID EliSpot reader software V7.0.

Anti-e1 IgG ELISA

Ninety-six well plates (NuncMaxiSorp™, Thermofisher) were coated overnight at 4°C with 50 µL of e1 peptide diluted at 10 µg/ml in 25 mM borate buffer pH 9. After washings in PBS Tween-20 (PBST) and blocking with 5% bovine serum albumin (BSA, Sigma-Aldrich) in PBST for two hours at 37°C, mouse (1:500-1:2000) or human (1:2000) sera were incubated in PBST and 0.1% BSA for two hours at 37°C. After three washes in PBST, the secondary antibody coupled to HRP (1:2000; Jackson ImmunoResearch) was incubated in PBST for one hour at 37°C. Fifty µl of TMB substrate (Sigma Aldrich) was added and reaction was stopped by adding 50 µl H_2SO_4 0.5 M. Optical density was read at 450 nm using Spark10M multimode microplate reader (Tecan). For human sample processing, sera were analyzed in duplicate with 2-3 plates per series. In each plate, eight serial dilutions of a pooled serum internal control from e1-immunized mice and eight serial dilutions of a human serum internal

control (pooled from three multiple sclerosis samples) served as positive day-to-day and plate-to-plate controls. Human specimens were considered High Responders (HR) when the optical density exceeded the cutoff value, set at 2.5 SDs above the mean of the healthy volonteers.

Kir4.1 immunofluorescence on mouse sections

Cryostat sections prepared from 4% paraformadehyde-perfused animals were subjected to heat induced epitope retrieval (HIER) in 10 mM Na-citrate buffer (pH 6.0) at 90°C, preincubated in PBS with 25% donkey serum and 0.03% Triton-X100 for 20 min at room temperature, followed by overnight incubation at 4°C with anti-e1 serum or rabbit anti-Kir4.1$_{356\text{-}375}$ Cter antibody (Alomone APC-035) in PBS, 2.5% donkey serum and 0.03% Triton-X100. After three PBS washes, sections were incubated for 45 min with donkey AF594-coupled F(ab')2 anti-mouse or AF488-coupled F(ab')2 anti-rabbit IgGs (1:1500) in PBS 2.5% NDS and 0.03% Triton-X100. Sections were incubated with DAPI at 1 µg/ml for 5 min, rinsed, and coverslipped with anti-fading mounting medium. To control the specificity of immunofluorescence, adjacent sections were immunostained in the absence of primary antibodies. Sections were analyzed on the fluorescent microscope and pictures were taken at x20 or x40 objective. Peptide-*N*-glycosidase F (PNGase; P0704S, New England BioLabs), which cleaves N-linked oligosaccharides in the context of N-X-S/T, was used to evaluate the effect of N-linked glycosylation on the anti-e1 reactivities. For this, HIER-treated sections were incubated with 5 U/µl of PNGase F in 10 mM PBS, 10 mM EDTA at pH 7.6 under Parafilm in a humidified chamber at 37 °C overnight, before being processed for KIR4.1 immunohistofluorescence.

Kir4.1 immunofluorescence on human tissue sections

Twelve µm fresh frozen sections were prepared on Superfrost Plus slides. Tissues containing subcortical WM and cortical gray matter from four multiple sclerosis patients, from one inflammatory lethal encephalitis and from a normal brain **(Supplementary Table 3)** were used from selected blocks containing inflamed subcortical WM or NAWM**(Supplementary Table 4).** Lesions were classified as 'active' (e.g. demyelination and homogeneous inflammatory infiltrate), 'chronically active' (e.g. demyelination and inflammatory infiltrate at the edges), or NAWM (e.g. no demyelination) without inflammatory infiltrates and meningeal infiltrates. Acetone-fixed sections were preincubated in PBS with 25% donkey serum and 0.03% Triton-X100 for 20 min at room temperature, followed by overnight incubation at 4°C with mouse anti-e1 serum and rabbit anti-Kir4.1$_{356\text{-}375}$ Cter antibody in PBS and 2.5% donkey serum and 0.03% Triton-X100. After three PBS washes, sections were incubated for 45 min with donkey AF594- anti-mouse or AF488- anti-rabbit IgGs at 1:1500 in PBS, 2.5% donkey serum and 0.03% Triton-X100. After the immunostaining procedure, sections were incubated with DAPI at 1 µg/ml for 5 min and with 0.1% Black Soudan in 70% ethanol to stain the white matter and hide lipofuscin-driven autofluorescence. Sections covered with anti-fade mounting medium were analyzed on the fluorescent microscope and pictures were taken at x40 objective using the digital camera at fixed exposure time. For fluorescence quantification of Kir4.1-immunoreactivity, three fields of subcortical WM per sample from control or active multiple sclerosis lesion sections were acquired and the average gray level of immunofluorescence was measured with ImageJ software. Sections from four human renal fresh-frozen biopsies performed for transplantation procedures were also used for Kir4.1 immunofluorescence: control cortical pre-implant biopsies from two donors and cortical kidney biopsies with chronic inflammation from two patients with interstitial fibrosis/tubular atrophy.

Cell cultures and tunicamycin treatment

The human U-251 astrocytoma cell line (U-251MG, RRID:CVCL 0021) was cultured in 10% fetal bovine serum/DMEM high glucose medium with penicillin-streptomycin (100 U/ml) and passaged when confluent. Cells were seeded on Nunc Lab-TekII Chamber Slide™ and experiments were carried out when cells had become 50-80% confluent. Cells were treated overnight with 1µg/ml tunicamycin (Sigma) or vehicle before being fixed 10 min with 2% paraformaldehyde in PBS at 4°C and further processed for immunohistofluorescence with rabbit anti-Kir4.1$_{356\text{-}375}$ Cter antibody (Alomone labs #APC-035), mouse or human HR anti-e1 serum followed by the donkey AF594-coupled F(ab')2 anti-mouse or anti-human IgGs and AF488-coupled F(ab')2 anti-rabbit IgG (1:1500) in PBS, 2.5% NDS and 0.03% Triton-X100. For competition experiments (e1-preadsorbed sera), mouse anti-e1 serum (1:100) or HR serum (1:50) were preincubated 36h at 4°C with e1 peptide at 20 µg/ml in PBS supplemented with 2% bovine serum albumin; the sera were then centrifuged (14,000g) during 30 min at 4°C to

remove antibody complexes before being applying to tissue sections at desired concentration. Slides were coverslipped with Prolong Gold DAPI mounting medium (Thermo Scientific) before taking pictures at x40 objective under the fluorescent microscope at fixed exposure time.

Western Blot

Crude membrane preparations were lysed in RIPA buffer with Protease Cocktail inhibitor (Sigma) and protein concentrations were determined using the BCA protein quantification kit (Pierce). Some samples (15 µg protein) were incubated at 37°C without or with 2 µl of PNGase F (5 U/µl) following instructions of the provider. Lysates were heated in LDS buffer (ThermoFisher) at 70°C for 10 min in the presence or absence of 1 mM DTT. A total of 10-15 µg protein was loaded in each lane of 4–12% NuPage BisTris gels (ThermoFisher) with a lane for the dual color molecular marker (BioRad). Electrophoresis was performed for 60 min at 200 V. The proteins were electrotransferred to a PolyVinylidene DiFluoride membrane (ThermoFisher) for 60-100 min at 40 V. Membranes were probed with the following antibodies revealed using the SuperSignal Pico (or Femto if specified) West reagents (Pierce): rabbit anti-Cter (356-375) or anti-e1(93-106) of Kir4.1 (#APC-035 and #APC-165 respectively, 1:2500-5000, Alomone labs[2,3]); mouse anti-e1 serum (1:800) or human HR serum (1:200); rabbit anti-human ATP1A1 (1:5000, Proteintech 55187-1-AP[4]) and corresponding peroxidase-conjugated donkey anti-mouse IgG, anti-human IgG or anti-rabbit IgG (JIR, 1:20,000). Bands were quantified when needed using Image J.

2. Cui Y*, et al.* Astroglial Kir4.1 in the lateral habenula drives neuronal bursts in depression. *Nature* **554**, 323-327 (2018).
3. Thuringer D*, et al.* Modulation of the inwardly rectifying potassium channel Kir4.1 by the pro-invasive miR-5096 in glioblastoma cells. *Oncotarget* **8**, 37681-37693 (2017).
4. Plossl K*, et al.* Identification of the retinoschisin-binding site on the retinal Na/K-ATPase. *PLoS One* **14**, e0216320 (2019).

Immunofluorescence experiments with transfected CHO cells

Chinese Hamster Ovary cells (CHO-K1, RRID:CVCL_0214) were cultured in DMEM high glucose with penicillin-streptomycin (100 U/ml) and 10% heat-inactivated fetal bovine serum (FBS), and passaged when confluent. Cells were transiently transfected using Lipofectamine 2000 and the expression vector pcDNA3.1 containing the human Kir4.1 sequence under control of the human cytomegalovirus promoter (gift from Dr J. Devaux, IGF CNRS-Université de Montpellier, France). N104Q Kir4.1 mutant plasmid was generated with Quikchange II XL site-directed mutation kit (Agilent) and Kir4.1 sequences were checked by Eurofins Genomics (France). After 36-48h transfection, the cells were incubated 5 min at 37°C in PBS and 5 mM EDTA, dissociated in PBS, 5mM EDTA and 10% heat-inactivated fetal bovine serum, centrifuged and fixed with 2% formaldehyde in PBS for 10 minutes. After centrifugation and redissociation in PES buffer (PBS, 2mM EDTA, 2% FBS) cells were distributed in a 96-well plate (3-4×10^5/well) and incubated overnight at 4°C with 100µl PES buffer containing 0.002% Triton-X100, rabbit C-ter Kir4.1 antibody (1:2000) and mouse anti-e1 serum (1:400) or no primary antibody (background control). After three washes, cells were incubated 45 min at room temperature with 100µl PES/Triton-X100 buffer containing donkey AF488-coupled anti-rabbit IgG (1:800) and AF594-coupled anti-mouse IgG (1:800). Subsequently, the cells were washed three times before to cytospining in 100µl PES buffer with Shandon Cytocentrifuge (Thermo Scientific) 3 min at 400g on Superfrost plus slides and coverslipped with Prolong Gold DAPI mounting medium before analysis under the fluorescent microscope. For each group (non transfected, WT Kir4.1 transfected, N104Q Kir4.1 transfected), slides were coded and DAPI+ cells (1700, 2136 and 1569, in total respectively) were analyzed by counting positive cells for Cter- and e1-immunofluorescence with the minimal fixed exposure time, set to avoid detection of lower fluorescent signals in the non-transfected group.

Experimental animal group allocations. All animals were acclimated 7-10 days to the animal facility before being randomly allocated to experimental group (4 to 5 per cage) and each cage was given a letter (alterning allocation to a specific group) by an individual not associated with the recording of the animals. The experimenter was blinded to experimental group allocation until the end of the protocol. The order in which the animals in the different experimental groups were assessed changed every day.

Statistics/Graphics Sofwware. GraphPad Prism 8.0 was used. No statistical method was used to predetermine sample size. Sample size was chosen based on prior experience for each experiment, to yield adequate power to detect specific effects. Sample size for each group is indicated for each figure.
